# Nothing wrong about change: the adequate choice of the dependent variable and design in prediction of cognitive training success

**DOI:** 10.1186/s12874-020-01176-8

**Published:** 2020-12-07

**Authors:** André Mattes, Mandy Roheger

**Affiliations:** 1 Department of Individual Differences and Psychological Assessment, University of Cologne, Pohligstraße 1, 50969 Cologne, Germany; 2 Department of Neurology, University Medicine Greifswald, Walther-Rathenau Str. 49, 17489 Greifswald, Germany

**Keywords:** Prognostic research, Simulation study, Methodology, Regression analysis, Cognitive decline, Cognitive training

## Abstract

**Background:**

Even though investigating predictors of intervention success (e.g Cognitive Training, CT) is gaining more and more interest in the light of an individualized medicine, results on specific predictors of intervention success in the overall field are mixed and inconsistent due to different and sometimes inappropriate statistical methods used. Therefore, the present paper gives a guidance on the appropriate use of multiple regression analyses to identify predictors of CT and similar non-pharmacological interventions.

**Methods:**

We simulated data based on a predefined true model and ran a series of different analyses to evaluate their performance in retrieving the true model coefficients. The true model consisted of a 2 (between: experimental vs. control group) × 2 (within: pre- vs. post-treatment) design with two continuous predictors, one of which predicted the success in the intervention group and the other did not. In analyzing the data, we considered four commonly used dependent variables (post-test score, absolute change score, relative change score, residual score), five regression models, eight sample sizes, and four levels of reliability.

**Results:**

Our results indicated that a regression model including the investigated predictor, Group (experimental vs. control), pre-test score, and the interaction between the investigated predictor and the Group as predictors, and the absolute change score as the dependent variable seemed most convenient for the given experimental design. Although the pre-test score should be included as a predictor in the regression model for reasons of statistical power, its coefficient should not be interpreted because even if there is no true relationship, a negative and statistically significant regression coefficient commonly emerges.

**Conclusion:**

Employing simulation methods, theoretical reasoning, and mathematical derivations, we were able to derive recommendations regarding the analysis of data in one of the most prevalent experimental designs in research on CT and external predictors of CT success. These insights can contribute to the application of considered data analyses in future studies and facilitate cumulative knowledge gain.

**Supplementary Information:**

The online version contains supplementary material available at 10.1186/s12874-020-01176-8.

## Background

In medical and psychological research, researchers and clinicians often study the effects of certain pharmacological and nonpharmacological interventions. One focus in the field of neuropsychology so far is the effects of non-pharmacological interventions, especially cognitive training interventions to delay or even prevent the onset of cognitive decline. Cognitive training (CT) interventions are defined as a standardized set of exercise [1], which involves repeated practice and is designed to reflect particular cognitive functions, such as memory, attention, or executive functions [2, 3]. CT is not only effective in improving and maintaining cognitive abilities in patients with neurological diseases such as Alzheimer’s [4] or Parkinson’s disease [5], but also in healthy older adults as an attempt to prevent cognitive impairment in the aging process [6]. Yet, in the course of the increasing importance of personalized medical approaches, the question: “Who benefits most from CTs” is gaining more and more attention. Defining prognostic factors for performance changes after nonpharmacological interventions is of high importance in order to define subgroups of participants who may benefit from a specific treatment [7, 8], and for the design of new and more effective training programs [9, 10]. For example, many studies have investigated the impact on sociodemographic variables such as age [11], sex [12], and education [13] as predictors of CT success. Yet, results on prognostic factors for changes in performance after nonpharmacological trainings so far are highly heterogeneous and in some cases contradictory. A study of Matysiak et al. (2019) investigated for example prognostic factors for changes in performance after a working memory training for healthy older adults. With the help of multi-level analysis they could show that older adults with initially lower working memory capacity (lower scores at study entry in the investigated domain) improved less and reached lower levels of performance [14]. This was explained with an approach called the magnification account, which predicts that cognitively efficient people also show the most gain in nonpharmacological interventions [15]. In contrast to that, a study by Zinke et al. (2014), also investigating predictors of working memory training success, revealed that participants with initially lower baseline performance were related to higher gains after training [16], using stepwise regression analyses for their calculation. Yet, to explain this result, a different explanatory account was used: the compensation account, which states that interventions will yield the largest gain in the least cognitively efficient people [15]. But how is it possible that two studies, which studied a similar topic (predictors of working memory training success) reveal such contradictory results? To answer this question, a systematic review on prognostic factors of memory training success in healthy older adults was conducted that could show that the results vary not only as a function of the type of statistical calculation used to determine prognostic factors, but also of the type of dependent variables used in the calculations [17]: post-test scores, change scores, relative change scores, and residual change scores. Post-test scores are hereby defined as performance after training/intervention, change scores refer to post-pre training scores, relative change scores are norm-referenced change scores, and residual change scores are defined as change scores adjusted for baseline variance. Moreover, the systematic review could show that different prognostic studies used different independent variables and variations of these as their prediction models: e.g. some studies did include “group” (Experimental vs. Control Group) as a binary predictor in their regression analyses, whereas some studies only calculated predictors within the experimental group. In some regression models, interactions between group variables and possible predictors were assessed, whereas in other studies these interactions were missing in the regression models. In addition to that, some studies calculated regression models that integrated neuropsychological performance at study entry as a possible predictor. A special role of neuropsychological performance at study entry was identified, leading to the two already mentioned explanatory accounts: magnification vs. compensation. However, a current paper of Smolén et al. (2018) could show that most evidence for the compensation account of nonpharmacological training interventions is unreliable due to methodological errors in the original studies [18]. As systematical error related to the choice of the dependent variable in a prognostic model and the special role of neuropsychological performance at study entry can theoretically be translated to all research fields which use multiple regressions to determine prognostic factors for changes after interventions, the present paper wants to establish a framework for the appropriate use of multiple regression analysis in the context of prognostic research, here with a special focus on CT interventions.

Therefore, in the present paper, with the use of simulation methods, we systematically investigate not only which multiple regression model is best suited to answer the question of “who benefits?” by calculating different regression models with different independent variables as possible predictors (Aim 1), but also take a look at the impact of these four different dependent variables in a multiple regression paradigm to determine which of these variables is the most suited one to investigate performance changes after intervention (Aim 2). Furthermore, we investigate the best sample size in relation to the amount of predictors used in these multiple regression model (Aim 3) and evaluate the influence of the reliability of instruments to measure predictors and outcomes (Aim 4). In a final step, we highlight the special role of the pre-test score as a predictor in the multiple regression analysis to shed further light on the discussion in context of the magnification and compensation account (Aim 5). We used CT as a specific example to illustrate the simulation process. However, our results can apply to many fields, which employ the simulated and discussed experimental design.

## Method

### Simulations

**Fig. 1:**
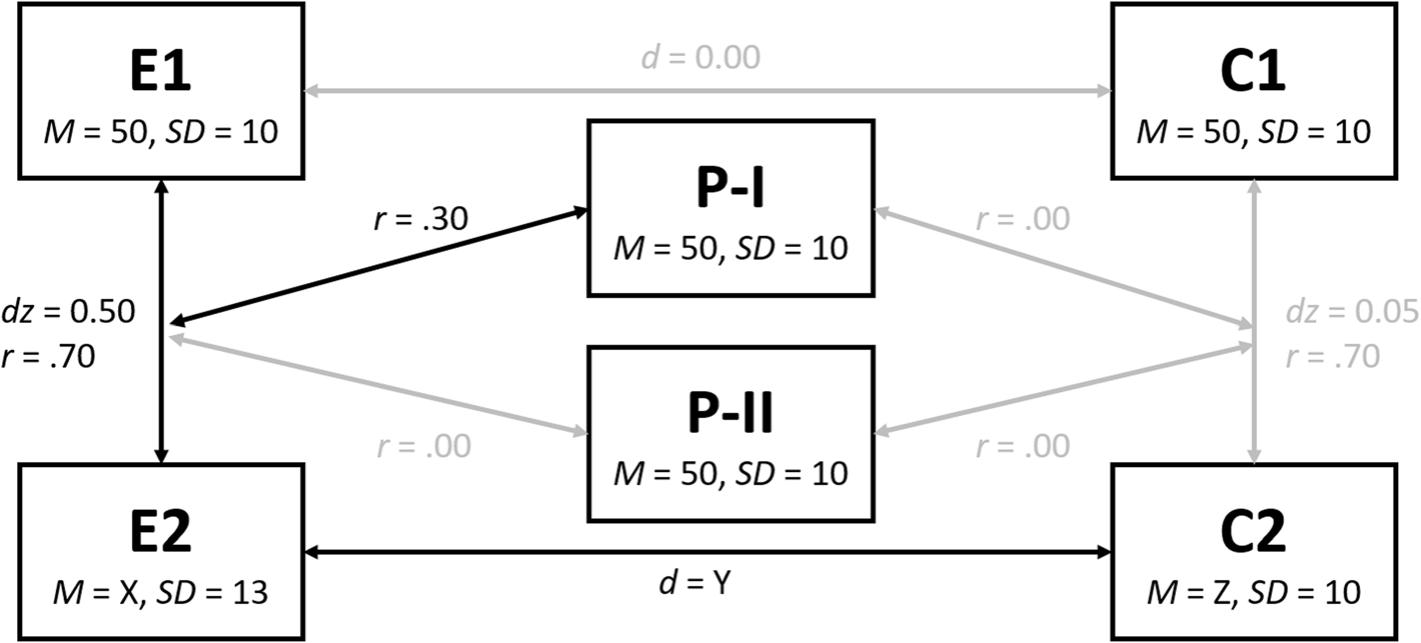
Overview of the simulated data. The mean X of E2 was computed depending on the level of reliability such that the desired effect size *dz* = 0.50 emerged given the mean and standard deviation of E1, the standard deviation of E2 and the correlation between E1 and E2. The same applies to the mean Z of C2. Accordingly, the effect size Y of *d* was variable across the levels of reliability. *Note.* Depicted arrows do not indicate causality or any direction of influence

We simulated data from a simple model which is often found in experimental designs reported in the literature, for instance of CT (e.g. [19], see Fig. 1). The model consists of a 2 (group: experimental vs. control) × 2 (time: pre-treatment vs. post-treatment) design, in which the group represents a between-subjects factor and the time represents a within-subjects factor. Additionally, a continuous predictor was included in the design which predicts the success of the treatment in the experimental group (e.g. age which has been identified as a predictor of CT success [11]). We also included a continuous predictor in our simulations which was not related to the success of the treatment (e.g. education [13, 20]).

We simulated the data in two steps. First, we randomly generated data derived from the true model as described below (see Model Specifications). Second, we added noise to these data given that measurements are never exact and measurement instruments always show a measurement error. We assumed that the noise is normally distributed and that the expected value of the noise is zero. These assumptions are based on the Classical Test Theory [21]. The extent of the noise thus depends on the standard deviation (*SD*) of the noise distribution, which is directly related to the reliability of the measurement instruments. Therefore, for our basic simulations, we determined the noise *SD* by setting the reliability for all measures to .80, reflecting good reliability [22, 23]. In a further step, we systematically varied the reliability of the measures and generated additional data assuming a reliability of .60 (acceptable reliability), .70 (moderate reliability), and .90 (excellent reliability).

Furthermore, we varied the sample size in our simulations: We ran simulations with a sample size of *n* = 50, 100, 150, 200, 250, 300, 400 and 500 participants, to investigate the impact of sample size on the detection of a desired effect.

For each sample size, we generated *n* = 1000 data sets as described above. We provide the simulated data and the R code here: www.osf.io/p54j3

### Model specifications

We determined a true model that we used to generate sample data. The model was as follows (see Fig. 1 for a summary): At time 1, i.e. before the treatment, both the experimental group (E1) and the control group (C1) had the same mean and standard deviation on the measure that we simulated (e.g. the score on a cognitive test). We used the norms of the T-scale as the values for the pre-treatment condition, i.e. *M*_E1/C1_ = 50 and *SD*_E1/C1_ = 10. At time 2, i.e. after the treatment, the mean in the experimental group (E2) was higher than at time 1 with a medium effect size of *dz*_E1-E2_ = 0.50, reflecting a successful treatment. Furthermore, we set the *SD*_E2_ to 13, i.e. a bit higher than at time 1, reflecting the common finding that the variance is larger in groups that were submitted to a treatment compared to groups that were not given an intervention. The *SD* of the control group (C2), however, was set to the same value as at time 1, i.e. *SD*_C2_ = 10. To account for the common observations that a given measure also increases in the control group from time 1 to time 2 despite the lack of treatment, we set the effect size *dz*_C1-C2_ to 0.05, reflecting a negligible increase.

Furthermore, we simulated two predictors (e.g. age (P-I) and education (P-II) in years, as frequently used as predictors in CT studies). Both predictors (P-I and P-II) had a mean of 50 and a standard deviation of 10. Importantly, P-I was correlated with the increase in the experimental group, *r*(P-I, ΔE1-E2) = .30, reflecting a medium effect. However, P-I was not correlated with the change from time 1 to time 2 in the control group, *r*(P-I, ΔC1-C2) = .00. The second predictor was not related to any change from time 1 to time 2, *r*(P-II, ΔC1-C2) = .00 and *r*(P-II, ΔE1-E2) = .00. We included this predictor in the simulations to examine whether the statistical models we tested (see Analyses) were able to discriminate between predictors that have a true effect and predictors that do not.

Note that the observed effect sizes (*dz* and *r*) also depend on the reliability [23]. In general, the higher the reliability is, the larger the observed effect sizes are given a constant true effect size. To account for this, we kept the true effect size constant. To this extent, we computed the true effect sizes in a scenario with medium effect sizes, i.e. *r* = .30 and *dz* = 0.50, and a good reliability, i.e. *r*_tt_ = .80. These true effect sizes were subsequently used as a basis for the true model for which we generated data as described above and on which we imposed different levels of noise reflecting the respective reliability. Accordingly, the observed effect sizes vary as a function of reliability while the true effect sizes remain constant, as can be assumed in a real-world setting.

### Analyses

After generating *n* = 1000 data sets for each sample size from the true model and imposing noise reflecting the respective reliability for all measures (E1, E2, C1, C2, P-I, P-II), we ran five different regression analyses on each individual data set (Aim 1, see Table 1). The different regression models differed in terms of the predictors included in the model (Aim 1). In Model 1, the dependent variable was predicted by the external predictors which might be associated with the treatment success, i.e. P-I and P-II. In Model 2, the score measured at time 1, i.e. the pre-test score, was added. Model 2 thus consisted of P-I, P-II and the pre-test score (i.e. E1 and C1) as the predictors of the dependent variable. In Model 3, we additionally added the treatment Group as a binary predictor (dummy-coded: 0 = control group, 1 = experimental group). Even though the treatment Group as a binary predictor is fundamental when calculating training success, we did not include it in Models 1 and 2 as not integrating this predictor is commonly observed in recent prediction research. Therefore, we want to show how not integrating the Group variable in the regression can influence the results and lead to misleading interpretations. In Model 4, the dependent variable was predicted by P-I, P-II, the pre-test score, Group and the interaction between P-I and Group, and P-II and Group. Finally, in Model 5, we removed the pre-test score from the model, such that Model 5 contained the predictors P-I, P-II, Group and the interaction between P-I and Group, and P-II and Group (see Table 1 for an overview). All continuous predictors (i.e. P-I, P-II, and pre-test score) were centered prior to entering them in the regression model to allow for a better interpretability. In Models 1 to 3, usually the regression coefficients for the predictors P-I and P-II are interpreted to investigate the prediction of CT success. In Models 4 and 5, the regression coefficients for the interaction term between the Group and the predictors P-I and P-II are of interest.

**Table 1:** Illustration of the predictors included in the regression models

	P	Time 1	Group	P x Group
Model 1	X			
Model 2	X	X		
Model 3	X	X	X	
Model 4	X	X	X	X
Model 5	X		X	X

*Note:* P = external predictors potentially associated with the treatment success (P-I, P-II); Time 1 = measurement score before the treatment (E1, C1); Group = treatment group (experimental vs. control); P x Group = Interaction between external predictors and treatment group

In addition to varying the predictors in the regression model, we also varied the dependent variable in order to investigate the consequences of the different measures used in the literature to quantify treatment success (Aim 2). Specifically, we used the following measures as dependent variables: (1) the measure at time 2 (E2, C2), i.e. the post-test score, (2) the absolute change from time 1 to time 2 (E2 minus E1, C2 minus C1), (3) the relative change from time 1 to time 2 (E2 minus E1, divided by E1; C2 minus C1, divided by C1), and (4) the residuals of the post-test score (E2, C2) after controlling for the pre-test score (E1, C1). We not only ran the regression analyses for the observed data, but also for the true data. This allowed us to compute a bias by subtracting the true regression coefficients from the observed regression coefficients (see below). Furthermore, we varied the sample size in our simulations: We ran simulations with a sample size of *n* = 100, 150, 200, 250, 300, 400 and 500 participants, to investigate the impact of sample size on the detection of a desired effect (Aim 3). We also varied the reliability for all measures with reliabilities of .60, .70, .80, and .90, to examine how the results are affected by measurement accuracy (Aim 4). Importantly, we fully crossed the set of predictors and the dependent variables, i.e. we computed each regression model for each dependent variable. This resulted in 20 regression analyses for each of the 1000 individual data sets generated for each of the eight sample sizes and each for the four levels of reliability.

We then aggregated the regression coefficients for each predictor by computing the mean of the coefficients for each set of predictors, each dependent variable, each level of reliability, and each sample size. Furthermore, we computed the standard deviation of these coefficients which is an estimate for the standard error (*SE*) of the regression coefficient, i.e. the precision with which the regression coefficient was estimated.

To evaluate the success of each model and each dependent variable of detecting a true effect while simultaneously controlling for the alpha error and to also highlight the specific role the performance of participants at study entry as a predictor (Aim 5), we proceeded as follows: for each set of predictors, each dependent variable, each level of reliability, and each sample size, we counted the number of times a given predictor yielded a significant relationship with the dependent variable (i.e. *p* < .05) and divided it by the total number of analyses (i.e. 1000). The resulting value *P* thus represents the proportion of significant effects of the given predictor in all analyses. If there is no true relationship between the given predictor and the dependent variable, *P* indicates the alpha error, i.e. the probability of finding an effect even though no true effect exists. If, however, there is a true relationship between the given predictor and the dependent variable, *P* indicates the power, i.e. the probability of detecting an effect when the true effect exists.

Furthermore, we computed the bias, i.e. the difference between the true regression coefficient and the observed regression coefficient. To compare the bias across different regression coefficients and different models with different units of the dependent variable (raw units for the post-score, the residuals and the absolute change; relative scores for the relative change), we studentized them. The unit of the studentized biases is “standard deviations”. To studentize a variable, its values are divided by its standard deviation. However, in our simulations, the standard deviation of the regression coefficient estimates is in fact the standard error of the estimates. Dividing by this *SE* would results in a larger studentized bias for large sample sizes given the smaller *SE* for large sample sizes. Accordingly, in our case, the regression coefficient estimates need to be divided by the product of their *SD* (i.e. their *SE*) and the square root of the sample size. This product is the actual *SD* of the estimates. In total, we ran 1,280,000 regression analyses (five models of four dependent variables and eight sample sizes, four reliability levels in 1000 datasets, for each data the true and the observed data).

## Results

### Aim 1: the choice of an adequate multiple regression model including all relevant predictors

The choice of the adequate regression model, i.e. the answer to the question which predictors should be included in the model, can be derived theoretically. First, it is obvious that the external predictor **P-I** needs to be included in the regression model since its prognostic performance is to be evaluated. Second, we need to account for the treatment that is applied to the experimental group, but not the control group. To this extend, we also need to include the binary predictor **Group** in the regression model.

Importantly, however, the external predictor P-I can only predict the outcome in the experimental group, but not in the control group, given that the control group is unaffected by the treatment and no systematic variations in the outcome variable (Aim 2) should be observed in this group. This relationship has to be modelled explicitly which is achieved by including the interaction of P-I and Group **P-I** ***×*** **Group** in the regression model. If this interaction term is not included in the regression model, a true relationship between P-I and the outcome variable might be overseen because it only exists in the experimental group but not in the control group. Jointly, this might lead to an insignificant main effect of P-I. Note, for instance, that the power to detect a significant effect of P-I in the Models 1 to 3 is much lower than the power to detect a significant effect of the P-I × Group interaction in the Models 4 and 5 (Table 2). Alternatively, a significant main effect of P-I in a regression model which does not include the interaction term P-I × Group cannot be interpreted as the ability of P-I to predict the intervention success because such an intervention success can only be observed in the experimental group. In this case, the significant main effect might just reflect a general relationship between P-I and the outcome variable (depending on which criterion is used, see Aim 2) which does not reflect the ability of P-I to predict the intervention success. To examine this, it is crucial to include the interaction term P-I × Group in the regression model.

**Table 2:** Results of simulations for **reliability of .80**, and sample size of ***n*** **= 200**

Coefficient	Model 1			Model 2			Model 3			Model 4			Model 5		
	***M***	***SE***	***P***	***M***	***SE***	***P***	***M***	***SE***	***P***	***M***	***SE***	***P***	***M***	***SE***	***P***
**Post-test score**															
Intercept	53.29	1.03	1.00	53.29	1.03	1.00	50.49	1.24	1.00	50.48	1.24	1.00	50.47	1.36	1.00
P-I	0.18	0.09	0.57	0.15	0.06	0.65	0.15	0.06	0.71	0.00	0.07	0.02	0.01	0.10	0.02
P-II	−0.00	0.08	0.04	0.00	0.06	0.05	0.00	0.06	0.05	−0.00	0.08	0.03	−0.01	0.10	0.03
Pre-test score				0.80	0.07	1.00	0.80	0.07	1.00	0.79	0.06	1.00			
Group							5.61	1.77	0.96	5.61	1.78	0.96	5.64	2.09	0.87
P-I x Group										0.30	0.11	0.71	0.36	0.17	0.59
P-II x Group										0.01	0.12	0.04	0.01	0.16	0.05
**Absolute change score**															
Intercept	3.26	0.89	0.99	3.26	0.89	0.99	0.46	1.13	0.19	0.45	1.12	0.20	0.46	1.13	0.18
P-I	0.14	0.06	0.57	0.15	0.06	0.65	0.15	0.06	0.71	0.00	0.07	0.02	0.00	0.08	0.02
P-II	0.00	0.06	0.05	0.00	0.06	0.05	0.00	0.06	0.05	−0.00	0.08	0.03	0.00	0.08	0.04
Pre-test score				−0.20	0.07	0.85	−0.20	0.07	0.87	−0.21	0.06	0.89			
Group							5.61	1.77	0.96	5.61	1.78	0.96	5.60	1.80	0.96
P-I x Group										0.30	0.11	0.71	0.28	0.12	0.63
P-II x Group										0.01	0.12	0.04	0.00	0.12	0.04
**Relative change score**															
Intercept	7.74	1.94	1.00	7.74	1.94	1.00	2.35	2.42	0.32	2.35	2.41	0.33	2.36	2.44	0.30
P-I	0.28	0.14	0.50	0.31	0.13	0.62	0.31	0.12	0.67	0.00	0.16	0.02	0.00	0.18	0.02
P-II	0.01	0.14	0.05	0.01	0.13	0.05	0.01	0.13	0.05	−0.00	0.16	0.03	0.00	0.18	0.04
Pre-test score				−0.61	0.18	0.97	−0.62	0.17	0.98	−0.63	0.17	0.99			
Group							10.77	3.80	0.93	10.77	3.81	0.93	10.75	3.91	0.92
P-I x Group										0.61	0.24	0.66	0.57	0.26	0.55
P-II x Group										0.01	0.25	0.04	0.01	0.26	0.04
**Residual score**															
Intercept	0.00	0.00	0.00	0.00	0.00	0.00	−2.80	0.89	0.90	−2.81	0.88	0.90	−2.79	0.88	0.90
P-I	0.15	0.06	0.65	0.15	0.06	0.65	0.15	0.06	0.71	0.00	0.07	0.02	0.00	0.07	0.02
P-II	0.00	0.06	0.05	0.00	0.06	0.05	0.00	0.06	0.05	−0.00	0.08	0.03	0.00	0.08	0.03
Pre-test score				−0.01	0.01	0.00	−0.01	0.02	0.00	−0.01	0.03	0.00			
Group							5.61	1.77	0.96	5.61	1.78	0.96	5.58	1.77	0.96
P-I x Group										0.30	0.11	0.71	0.29	0.11	0.71
P-II x Group										0.01	0.12	0.04	0.00	0.11	0.04

*Note.* The investigated regression models are displayed in the columns and the investigated dependent variables are displayed in the rows. The results of all other reliabilities and sample sizes (with reliability scores .60, .70, .80, and .90, and sample sizes of n = 50, 100, 150, 200, 250, 300, 400, 500) are displayed in the Supplementary Material Tables 1–32

Finally, we recommend also including the pre-test scores as a predictor in the regression model. This controls for differences in the variable of interest that were present prior to the intervention, similar to a covariate in an analysis of covariance. Our simulations show that models including the pre-test score as a predictor yield a better power to unveil a significant P-I main effect or P-I × Group interaction effect than models that do not include the pre-test score as a predictor. For example, Table 2 shows that for a reliability of *r*_tt_ = .80 and a sample size of *n* = 200, Model 5 without the pre-test score as a predictor yields a power of 0.63 to detect a significant P-I × Group interaction effect for the absolute change. Model 4, which does include the pre-test score as a predictor, yields a much higher power of 0.71. A similar pattern is found for the other criteria. An exception to this observation are models that use the residual score as the criterion because the residual scores are defined as the post-test score after controlling for the pre-test score. Consequently, the pre-test score can never significantly predict the residual test score and including or excluding the pre-test score in the model does not impact the regression coefficients of the other predictors. In the section on **Aim 5**, we discuss the special role of the pre-test scores as a predictor in the regression models in more detail.

**Fig. 2:**
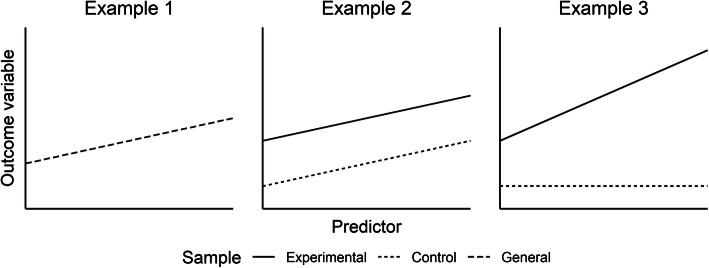
Illustration of different regression models. The Figure illustrates the relationship between a continuous predictor and an outcome variable depending on whether the regression model only comprises the continuous predictor (Example 1), the continuous predictor and the binary group variable (Example 2), or the continuous predictor, the binary group variable and their interaction term (Example 3). The solid line indicates the relationship in the experimental group. The dotted line indicates the relationship in the control group, and the dashed line represents the relationship regardless of the group assignment

Apart from the power to detect the desired effect, the interpretation of the regression coefficients also varies between the different regression models. In the Models 1 to 3, the coefficients of the continuous predictors indicate how the outcome variable changes when the corresponding predictor increases by one unit. For example, in Model 2 for the absolute change score as the criterion, an increase of one unit of P-I would lead to an increase of 0.15 units in the absolute change (Table 2). Additionally, in Model 3, the coefficient for the binary Group variable indicates the mean difference in the outcome variable between the experimental group and the control group. Since the Group variable was dummy-coded (0 = control group, 1 = experimental group), the coefficient informs about the deviation of the experimental group from the control group in terms of the outcome variable. The intercept indicates the predicted mean of the outcome variable for mean values of all continuous predictors (Models 1 to 3) and in the control group (only for Model 3). This explains why the intercept is lower in Models 1 and 2 than in Model 3. In the first two models, the Group variable is not accounted for, thus the intercept represents the overall mean in the sample. In Model 3, the Group variable is taken into account. Since the control group was modelled to have lower values on the outcome variable than the experimental group, the intercept is lower compared to the other two models (see also Fig. 2).

The interpretation is slightly different for the Models 4 and 5 which include interaction terms. Specifically, the interpretation for the continuous predictors is limited to the control group, i.e. the regression coefficients for P-I, P-II (and the pre-test score) indicate the change in the outcome variable for the control group if the predictors increase by one unit. Ideally, these should be zero (except for the pre-test score) because the predictor P-I is expected to predict the intervention success and there was no intervention in the control group. The regression coefficients for the interaction terms indicate how much more (or less) the outcome variable changes in the experimental group compared to the control group when the continuous predictor increases by one unit. Take Model 4 for the absolute change score as the criterion for example: If P-I increases by one unit, the absolute change score does not change at all in the control group (Group = 0) because the regression coefficient for P-I is 0.00. In the experimental group (Group = 1), the absolute change score would change by 0.00 + 0.30 = 0.30 units, i.e. the sum of the regression coefficient for P-I and regression coefficient for the P-I by Group interaction. Finally, the regression coefficient for the binary group variable indicates the mean difference in the outcome variable between the experimental and control group for mean values on the continuous predictors, i.e. if the predictors are zero.

Fig. 2 illustrates how the interpretation of regression coefficients changes depending on whether the regression model only comprises a continuous predictor (Example 1; Models 1 and 2), a continuous predictor and the binary group variable (Example 2; Model 3), or a continuous predictor, the binary group variable and their interaction (Example 3; Models 4 and 5). In Example 1, there is only one regression line for the entire sample, ignoring the assignment to the experimental or control group and thus weakening the power to detect the effect. In Example 2, there are two regression lines – one for the experimental group and one for the control group – that are parallel to each other and have the same slope as the regression line as in Example 1 (but different intercepts) which also weakens the power to detect the effect. Finally, in Example 3, the slopes of the regression lines differ between the experimental and the control group. Ideally, the slope of the control group is zero, indicating that the predictor cannot predict the intervention success in the control group (because there was no intervention). The slope of the experimental group should be larger in Example 3 than in Example 2, because the impact of the predictor in the experimental group can now be isolated from the impact in the control group which is why the power to detect the effect is overall higher than in the other two examples.

To conclude, we strongly favor a regression model with the following predictors: P-I, Group, pre-test score, and P-I × Group. For an overview of all calculated models for the different dependent variables, reliability scores, and sample sizes see Supplementary Material Tables 1–32.

### Aim 2: the choice of an adequate criterion variable for the regression model

As a recent systematic review on prognostic factors of performance changes after memory training in healthy older adults could show, the type of dependent variables used for prognostic factor calculations differs across different studies [17]. Post-test scores, change scores, residual scores, and relative change scores were used to measure performance changes. Yet, all these types of dependent variables have different implications as regards content and interpretation.

In a classical pre-post design, which underlies most studies on CT, the **post-test score** seems to be an established dependent variable in multiple regression analyses measuring training success. However, using the post-test score (that is performance after training/intervention) answers the question “Is x a likely cause of y” [24], but does not refer to gain. Furthermore, imagine an external predictor such as P-I emerged as a significant predictor of the post-test score in the experimental group. Would that indicate that the external predictor can predict the intervention success? Not necessarily, because the predictor might just be related to the construct captured by the post-score. In this case, one would also find that P-I is similarly related to the pre-test score in the experimental group. Furthermore, an external predictor such as P-I could be related to the post-test score in both the experimental group and the control group. Thus, finding a significant effect of P-I on the post-test score in the experimental group is necessary, but insufficient to draw the conclusion that P-I can predict the intervention success.

**Absolute change scores** (post-pre performance) answer the question “whose score is most likely to increase/decrease over time?”, therefore directly referring to intervention gain [24]. Yet, change scores are under high criticism due to the fact that subtracting pretest scores from post-test scores are in discredit to lead to fallacious conclusions, because they are systematically related to random measurement errors [25] and are sensitive to regression to the mean. However, these criticisms are unfounded under a plausible regression model, which does not integrate the dependent variable as an independent variable [26]. Also, with the advent of structural equation modeling, which permits modeling of error-free constructs, much of the criticism on change scores in the literature has decreased further [27]. Change scores are easy to interpret (changes in the individual’s level of performance [28]), may help to remove unexplained variance, and change score models are appropriate whenever pre-test scores can be assumed to remain stable over time if no treatment occurs, that is, when pre-test scores are useful baseline measures [29].

A further type of dependent variable, which may be used in studies investigating intervention success, are **relative change scores.** Relative change scores are norm-referenced, which are inherent in traditional reliability or generalizability coefficients [28]. They can be interpreted in terms of how much progress an individual in comparison to others has made. Therefore, the focus is not on changes in the individual’s performance, but on comparisons to others. Yet, our simulations demonstrated that the relative change scores are more vulnerable to the methodological artifact (described by 18) than absolute change scores. The probability of detecting a significant negative regression coefficient for the pre-test score was consistently higher for relative change scores than for absolute change scores, regardless of sample size, regression model used, or level of reliability. Keep in mind that we did not model a relationship between the pre-test score and the intervention success when simulating the data. The indication of a significant negative regression coefficient is thus an alpha-error. Similarly, the power of detecting a significant P-I × Group interaction effect was consistently higher for absolute change scores than for relative change scores, regardless of sample size, regression model used, or level of reliability. Consequently, our simulations have shown that relative change scores are inferior to absolute change scores as criteria in regression models.

**Residual scores**, which are calculated by regressing dependent variable of a construct onto an assessment measured at baseline, provide a simple change score adjusted for baseline variance [30] and are in literature often referred to as a more appropriate method of measuring change in constructs over time than post-pre change scores [31]. Yet, residual score models ask slightly different questions than the change score models: Residual score models assume that post-test scores are a linear function of pre-test scores and that this function is not necessarily 1 [29].

Our simulations showed that when including the pre-test score as a predictor in the regression model, the regression coefficients for the other predictors are identical for post-test scores, absolute change scores and residual scores. In other words, as long as the pre-test score is a predictor in the regression model, it does not matter whether post-test scores, absolute change scores or residual scores serve as the criterion because they yield the same regression coefficients for the other predictors in the model (for a more thorough discussion of this phenomenon, see Aim 5).

### Aim 3: the choice of an adequate sample size

**Fig. 3:**
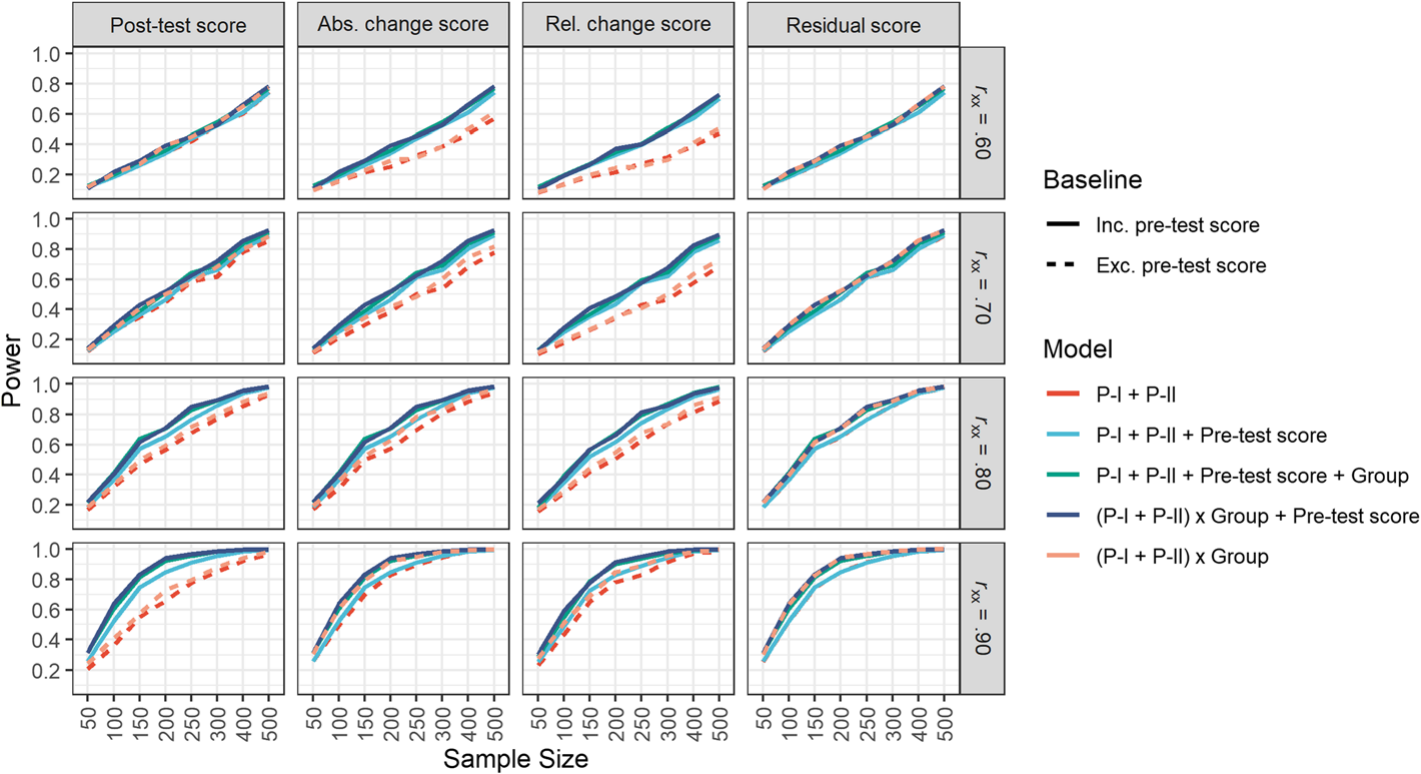
Overview of the power for the P-I or P-I x Group regression coefficient. The different dependent variables are displayed in the columns. The levels of reliabilities are displayed in the rows. The x-axis indicates the sample size. The different regression models are colour-coded as indicated in the Figure legend

We ran simulations with a sample size of *n* = 50, 100, 150, 200, 250, 300, 400 and 500 participants to investigate the impact of sample size on the detection of a desired effect of P-I or P-I x Group (if the interaction term was included in the regression model). The results for each dependent variable, each regression model and each level of reliability are displayed in Fig. 3 (for P-II and P-II x Group, see Supplementary Material Figure S1). Obviously, due to the fact that sample size and power are dependent on each other, as the sample size increases, the power increases, regardless of which dependent variable is used in the regression model. Further, as an overall trend it can be stated that the power is also dependent on the reliability; as the reliability increases, a smaller sample size is needed to achieve the same level of power.

As depicted in Fig. 3, not integrating the pre-test score in our regression model leads to the need of a higher sample size to achieve the same power as regression models which integrate the pre-test score in the calculation. This is the case for all dependent variables except one: when using the residual score as a dependent variable, there is nearly no difference in power/sample size increase between regression models that in- or exclude the pre-test score, as the pre-test score is already included in the dependent variable as a defining character of the residual score.

Overall, Fig. 3 shows that, regardless which dependent variable and which predictors (of the ones investigated here) are used in the calculation, it is important to at least use a sample size of *n* = 250 to *n* = 300 such that a power of at least .50 (independent of the reliability) is achieved. Due to the fact that often in experimental designs and/or research on new clinical patient groups the reliability of the used measures is either not known or not well established, a sample size of *n* = 250 therefore ensures an at least moderate power for the worst case that your dependent measure is not as reliable as you wish it would be.^1^ Yet, when using the change score as the dependent variable in the calculation and the reliability is rather low (.60/.70), a sample size of *n* = 300 seems even more appropriate to achieve a good power. It is important to always calculate and report reliabilities of the used instruments to ensure good scientific practice and help other researchers to better understand and evaluate your results.

### Aim 4: the role of reliability of the measurement instruments

**Fig. 4:**
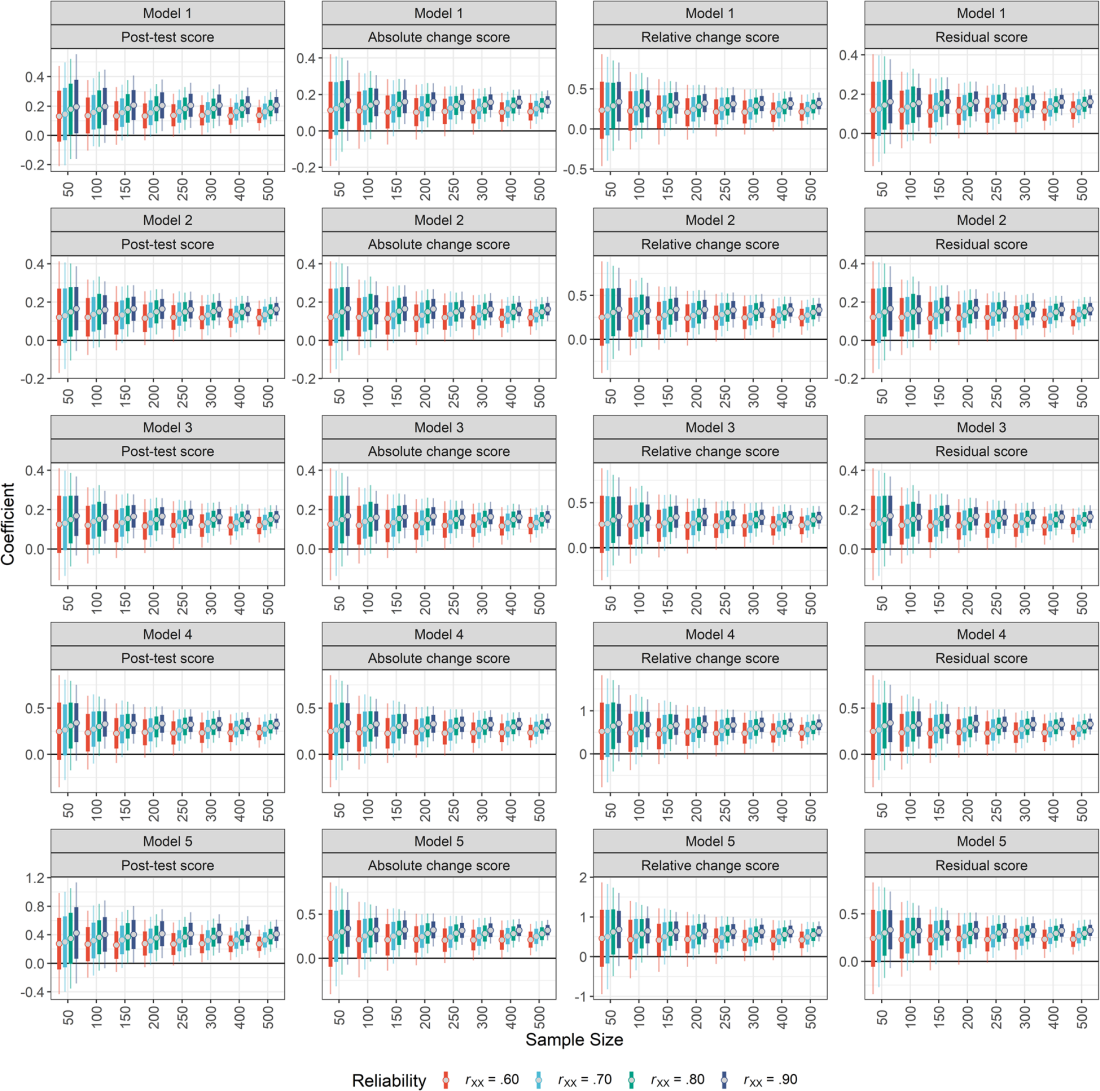
Overview of the regression coefficients of P-I or P-I x Group. The different regression models that were tested are displayed in the rows (Model 1 to 5) and the different dependent variables are displayed in the columns. In each subplot, the x-axis indicates the sample size and the y-axis the value of the regression coefficient for the predictor P-I or the P-I x Group interaction, depending on whether the respective model comprised the interaction term or not. For each sample size, the reliability is colour-coded. The dot indicates the mean of the regression coefficient distribution generated by simulating the data. The thick line covers the interval of the mean plus/minus one standard error and the thin line represents the 95% confidence interval. *Note*: Red colour indicates a reliability of .60; blue colour indicates a reliability of .70; green colour indicates a reliability of .80; purple colour indicates a reliability of .90. Model 1: P-I + P-II; Model 2: P-I + P-II + Pre-test score; Model 3: P-I + P-II + Pre-test score + Group; Model 4: (P-I + P-II) x Group + Pre-test score; Model 5: (P-I + P-II) x Group

The simulations show that, in order to achieve adequate power to detect a true effect, a relatively large sample size is required which is often difficult to achieve in scientific practice. However, the simulations also illustrate that an adequate power can not only be achieved by increasing the sample size, but also by selecting more reliable measures. While increasing the sample size mostly decreases the standard error which in turn leads to an increased power, increasing reliability also increases the estimates of the regression coefficient, i.e. the estimate and its entire confidence interval is shifted away from zero, making it more likely that a true effect is detected (see Fig. 4 for the regression coefficients of P-I or P-I x Group as a function of dependent variable, regression model, sample size and reliability, and Supplementary Material Figure S2 for P-II or P-II x Group).

Although at first sight, this observation might cause confusion, it can easily be explained by the fact that imperfectly reliable measures limit the maximum correlation that can be observed [32]. For example, assuming a true correlation of *r* = .50 between two variables that were measured with a reliability of *r*_tt_ = .60, the observed correlation will amount to *r* = .30, i.e. the true correlation multiplied by the square root of the product of both reliabilities [32]. Increasing the reliability to *r*_tt_ = .90, the observed correlation will amount to *r* = .45, approximating the true correlation of *r* = .50.

**Fig. 5:**
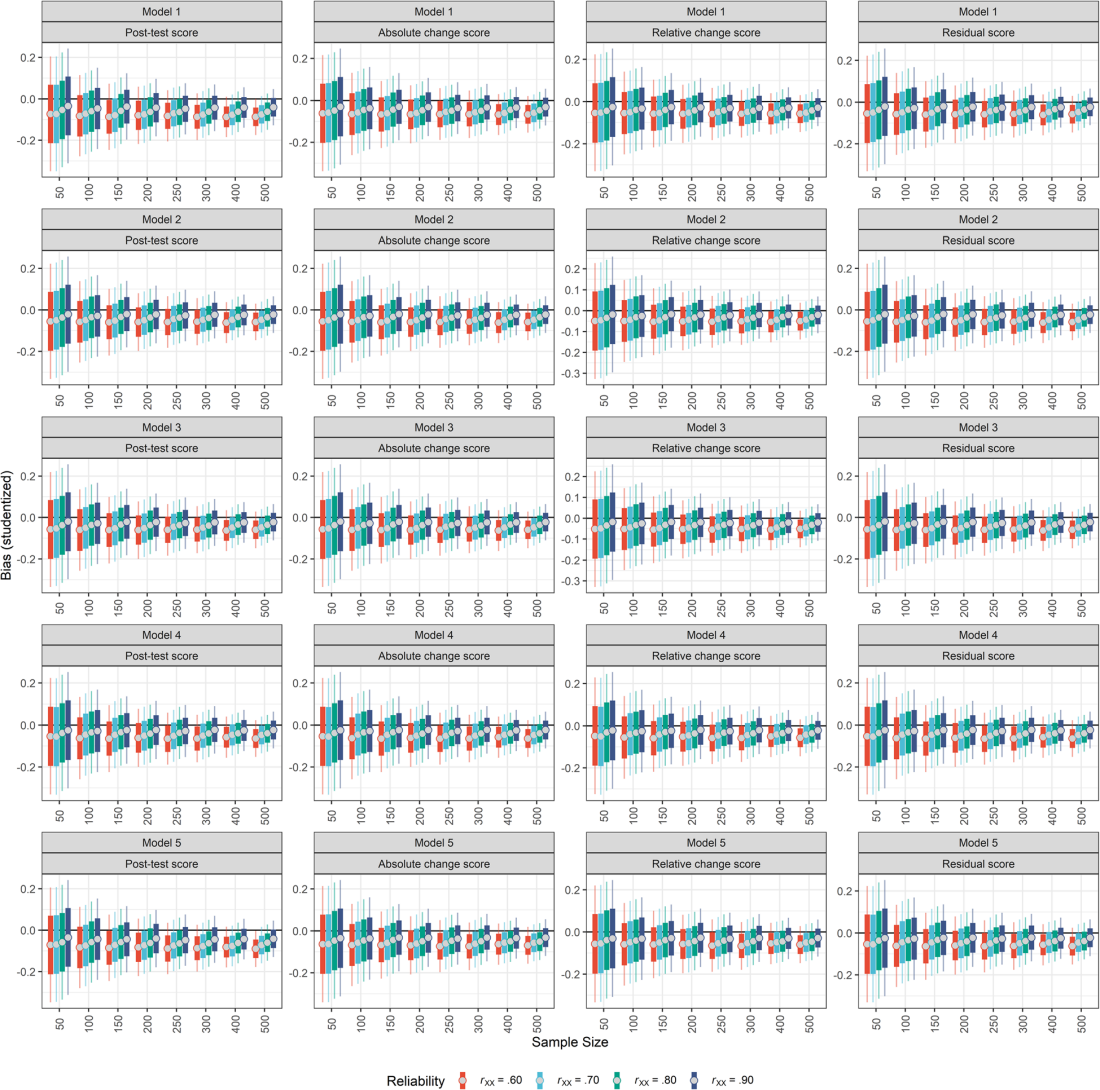
Overview of the studentized bias of the regression coefficients of P-I or P-I x Group. The different regression models that were tested are displayed in the rows (Model 1 to 5) and the different dependent variables are displayed in the columns. In each subplot, the x-axis indicates the sample size and the y-axis the studentized bias for the predictor P-I or the P-I x Group interaction, depending on whether the respective model comprised the interaction term or not. For each sample size, the reliability is colour-coded. The dot indicates the mean of the bias distribution. The thick line covers the interval of the mean plus/minus one standard error and the thin line represents the 95% confidence interval. A bias of zero would indicate that the observed regression coefficient is identical to the true regression coefficient. *Note*: Red colour indicates a reliability of .60; blue colour indicates a reliability of .70; green colour indicates a reliability of .80; purple colour indicates a reliability of .90. Model 1: P-I + P-II; Model 2: P-I + P-II + Pre-test score; Model 3: P-I + P-II + Pre-test score + Group; Model 4: (P-I + P-II) x Group + Pre-test score; Model 5: (P-I + P-II) x Group

Employing more reliable measurements in research thus not only increases the probability of detecting a true effect, but also reduces the bias, because true effects are estimated more precisely (see also Fig. 5 and Supplementary Material Figure S3). Note that reliability can not only be increased by employing more reliable measures, but also by repeating measures or by assessing a construct of interest by multiple tests instead of only one test [33, 34]. In other words, if a researcher wishes to increase the power of their study, but it is hardly possible to increase the sample size, they could increase the number of measures/measurements instead.

### Aim 5: the special role of the pre-test score as a predictor in a multiple regression

Studying Table 2 (or Tables 1–32 in the Supplementary Material), a striking observation is that whenever the pre-test score is included in a regression model, the regression coefficients for the other predictors yield the exact same results independent of the criterion, apart from the relative change because relative change is measured on another scale than the other three criteria. This suggests that whenever the pre-test score is a predictor in the model, the choice of the criterion (among post-test score, residual score, and absolute change score) is redundant.

Furthermore, the regression coefficient of the pre-test score for the post-test score and the absolute change score are a linear transformation of each other: the coefficient for the post-test score equals the coefficient of the absolute change score plus one. Note that although we did not model a negative relationship between the pre-test score and the absolute change, a negative regression coefficient emerges consistently and even reaches a high probability of reaching statistical significance for larger sample sizes, giving way to the faulty interpretation in favor of a compensation effect.

In the following, we briefly explain both observations mathematically. The regression equation for a model with the post-test score (*T*_2_) as the criterion and the centered pre-test score (*T*_1_) and any other variable *V* as predictors can be written as follows:

1 $$ {T}_2={b}_0+{b}_1{T}_1+{b}_2V $$

with *b*_0_ indicating the intercept, *b*_1_ the regression coefficient for the pre-test score, and *b*_2_ the regression coefficient for the additional predictor. Analogously, the regression equation for a model with the absolute change score (${T}_2-\left({T}_1+\overline{T_{1_{nc}}}\right)$) as the criterion and the pre-test score and another variable as predictors can be written as follows:

2 $$ {T}_2-\left({T}_1+\overline{T_{1_{nc}}}\right)={c}_0+{c}_1{T}_1+{c}_2V $$

with *c*_0_ indicating the intercept, *c*_1_ the regression coefficient for the pre-test score, and *c*_2_ the regression coefficient for the additional variable. Note that the absolute change score is computed by subtracting the non-centered pre-test score (${T}_1+\overline{T_{1_{nc}}}$) from the non-centered post-test score *T*_2_ and that the non-centered pre-test score consists of the centered pre-test score *T*_1_ plus the mean of the non-centered pre-test score ($\overline{T_{1\_ nc}}$; “nc” for “non-centered”). Resolving Eq. (2) for T2 and combining Eqs. (1) and (2) results in

3 $$ {b}_0+{b}_1{T}_1+{b}_2V={c}_0+{c}_1{T}_1+{c}_2V+{T}_1+\overline{T_{1\_ nc}} $$

which equals

4 $$ 0={c}_0-{b}_0+\overline{T_{1\_ nc}}+{T}_1\left({c}_1-{b}_1+1\right)+V\left({c}_2-{b}_2\right) $$

For this equation to be true for all values of *T*_1_ and *V*, the terms (*c*_1_ – *b*_1_ + 1) and (*c*_2_ – *b*_2_) each have to equate to zero (assuming the absence of multicollinearity, a formal prerequisite for a multiple regression analysis), giving

5 $$ {b}_1={c}_1+1 $$

and

6 $$ {b}_2={c}_2 $$

This also implies that the term ${c}_0-{b}_0+\overline{T_{1\_ nc}}$ also has to equate to zero, giving

7 $$ {b}_0={c}_0+\overline{T_{1\_ nc}} $$

First, these mathematical equations show that when the pre-test score is included as a predictor in the regression model, the regression coefficients for the other predictors are identical for the post-test score and the absolute change score as criteria (assuming that formal prerequisites for multiple regression analyses are met).

Second, the intercepts can be linearly transformed into each other. The intercept for the post-test score as the criterion equals the intercept for the absolute change score as the criterion plus the mean of the non-centered pre-test score. In case the continuous predictors are not centered prior to entering them into the regression models, the intercepts will be identical for the post-test score and the absolute change score as criteria.

Third and most importantly, the regression coefficients of the pre-test score for both criteria are a linear transformation of each other. Considering that the coefficient *b*_1_ reflects the relationship between the pre-test score and the post-test score, it can be interpreted as an estimate of the (test-retest) reliability. The coefficient *c*_1_ reflecting the relationship between the pre-test score and the absolute change score is thus always negative, because the reliability can never exceed 1 and because we have shown that *c*_1_ = *b*_1_–1. Furthermore, this relationship paradoxically implies that the relationship between the pre-test score and the change score is larger when the reliability of the measure is lower.

Smoleń et al. (2018) notes that many of the correlations between pre-test scores and absolute change scores reported in the literature to support the compensation account are suspiciously high, especially considering the theoretical limit of observable correlations given the imperfect reliability of psychological measures [18]. Here, we have demonstrated that these high correlations might in fact reflect low reliabilities of the measures used in the respective studies, which is in line with Smoleń’s mathematical demonstrations of why negative correlations between pre-test scores and absolute change scores emerge naturally [18].

## Discussion

As prognostic research and especially studies on the impact of parameters predicting the success of CT (or in general pharmacological and nonpharmacological interventions) have become of huge scientific interest over the past few years, the present paper aimed at systematically showing and discussing different types of regression models and dependent variables used, as well as the influence of reliability of measures, sample sizes, and the specific role of baseline measurements (pre-test scores) as predictors in multiple regressions to account for changes after interventions. With the help of simulation methods and mathematical derivations we could show that (Aim 1) a regression model including P-I, Group, pre-test score, and P-I × Group as predictors seems most convenient when investigating predictors of changes after interventions such as CT, as well as (Aim 2) using the absolute change scores as the dependent variable. Further, (Aim 3) studies should use at least a sample size of *n* = 250 and (Aim 4) one should take care of the reliability of used measures and their impact on the calculations. Finally, (Aim 5), although the pre-test score should be included as a predictor in the regression model for reasons of statistical power, its coefficient should not be interpreted because chances are high that even if there is no true relationship, a negative and statistically significant regression coefficient emerges.

In clinical research, especially when investigating specific patient populations, it is often difficult to recruit large sample sizes. For some patient populations or areas, a sample size of *n* = 250 is even utterly unrealistic. Yet, one has to be aware of the fact that when conducting multiple regression analyses to detect possible predictors of interventions in a relatively small sample, the power of the analysis is lacking. Therefore, it is even more important to ensure a high reliability of the used clinical tests and paradigms tested. This implies that already established tests have to be validated regarding their reliability norms when used in “new” clinical populations, in case that no test norms are available for this population. Further, reliability scores of the used tests should always be reported as they may help to inform whether the regression coefficient for the pre-test score is purely a statistical artefact or might reflect a relationship that persists beyond the statistical artefact. In the context of cumulative research evidence, it is also of high importance to report and publish studies with small sample sizes that only or mostly show non-significant prognostic effects. These studies can also contribute to cumulative research findings (e.g. in meta-analysis). This cumulative gain of knowledge is further facilitated if a joint methodological approach such as the one we suggest here is used, as this makes statistical results more comparable across separate studies.

Our simulations (and subsequent mathematical proof) also showed that unless the measures are perfectly reliable, there will always be a negative regression coefficient for the pre-test score predicting the absolute change score, even when there is no true relationship between them. In fact, the regression coefficient is the more negative, the less reliable the measures are. Thus, the negative regression coefficient should never be interpreted in favour of the compensation hypothesis. Our results support the concerns raised by Smoleń et al. (2018) regarding the validity of the evidence reported in the literature in favour of the compensation hypothesis.

In medical research, guidelines for prognostic research exist [35], which focus in detail on the design, conduction, and reporting of prognostic factor research, hereby differentiating between prognostic factor studies (a single prognostic factor that aims to predict a future outcome) and prognostic model studies (defined as a set of multiple prognostic factors to predict a future outcome). Yet, until now, there was no clear recommendation on the specific statistical methods which should be used when calculating multiple regressions to investigate these predictors in the realm of CT. Our present paper also emphasizes the need for the choice of the adequate dependent variable for prognostic research on different continuous outcomes after specific interventions and gives recommendations regarding the choice of the adequate regression model that should be used, as well as adequate sample size, reliability of outcome measures, and integration of baseline measurements. Therefore, when conducting prognostic research, a clear statistical rational should be provided. Furthermore, the present recommendations as well as the already existing medical guidelines on prognostic research should be adapted also for studies conducted in other fields (e.g. neuropsychology) to ensure a good practice and reporting of prognostic studies and results.

### Limitations

We are aware that the results of simulations strongly depend on the input to the simulations. In our case, we explicitly modelled an effect of the external predictor on the absolute change score in the experimental group. This decision was based on profound theoretical considerations. While it may not be surprising that the result of simulations favoured the inclusion of the interaction between P-I and the Group, and the absolute change score as the criterion, the simulations demonstrated the consequences of applying a range of statistical models (different combinations of predictors and criteria) to data that were generated by a different true model. Furthermore, we hope to have conveyed why we believe the true model we chose was the most reasonable of the models we considered in our simulations.

## Conclusion and recommendations

We systematically investigated the impact of different regression models, dependent variables, sample sizes and levels of reliability on the conclusions drawn from the respective analyses. Extensive simulations allowed us to derive well-considered recommendations for future analysis of data in one of the most common experimental designs in research on CT and prediction of CT success. Furthermore, we mathematically showed that the choice of dependent variable is redundant if the pre-test score is a predictor in the regression model, but that the corresponding regression coefficient should not be interpreted, preventing unjustified conclusions.

For future prognostic studies on predictors of changes after an intervention, we thus recommend the following analysis pipeline: Prior to data collection, determine the required sample size by considering the effect sizes you expect (e.g. based on previous findings) and the reliability of the measures you employ. Compute the absolute change scores and enter them as the criterion in a regression model. Include the pre-test scores, the group variable, the external predictor variables which you want to investigate, and the interactions between the external predictor variables and the group variable as predictors in the regression model. If you find a significant interaction effect, perform a post-hoc analysis. If the external predictor variable is able to predict the intervention success, it should only be related to the outcome variable in the experimental group, but not in the control group. Do not interpret the regression coefficient of the pre-test score, since it will always be negative (if your pre-test and post-test scores correlate positively). Keep in mind that less reliable pre- and post-test scores will produce a larger (negative) regression coefficient, regardless of whether there is a true pre-test score effect on the change score or not. Apart from reporting the sample size, also report the reliability of the employed measures as it has a considerable impact on the probability of detecting a true effect and should thus be made accessible to your readers.

## Supplementary Information

**Additional file 1.**

## Data Availability

The datasets generated and analysed during the current study are available in the Open Science Framework (OSF) repository: www.osf.io/p54j3

## Abbreviations

CT: Cognitive Training
SD: Standard Deviation
SE: Standard Error

## Footnotes

1 Note that as a good scientific practice of course it is important to ensure that all used tests and dependent measures have a moderate to high reliability established for the participant or patient group you investigate. See also “Aim 4: the role of reliability of the measurement instruments”.
