# Supplementary material for: Nothing wrong about change: the adequate choice of the dependent variable and design in prediction of cognitive training success
Source: BMC Med Res Methodol. 2020 Dec 7;20:296. doi: 10.1186/s12874-020-01176-8 (PMC7720538; doi:10.1186/s12874-020-01176-8)
Supplement: Supplementary file 1 — Additional file 1. [file 12874_2020_1176_MOESM1_ESM.docx]

**Supplementary Material:**

Nothing wrong about change: The correct choice of the dependent variable and design in prediction of intervention success

André Mattes^a*^ & Mandy Roheger^b*^

*^a^ Department of Individual Differences and Psychological Assessment, University of Cologne, Pohligstraße 1, 50969 Cologne, Germany; andre.mattes@uni-koeln.de*

*^b^ Department of Neurology, University Medicine Greifswald, Walther-Rathenau Str. 49, 17489 Greifswald, Germany; mandy.roheger@med.uni-greifswald.de*

**Shared First Authorship*

Table 1: Results of all investigated simulations ordered according to their used model and dependent variable, for a **reliability of .60**, and a sample size of ***n* = 50**

| **Coefficient** | **Model 1** | | |  | **Model 2** | | |  | **Model 3** | | |  | **Model 4** | | |  | **Model 5** | | |
| --- | --- | --- | --- | --- | --- | --- | --- | --- | --- | --- | --- | --- | --- | --- | --- | --- | --- | --- | --- |
|  | ***M*** | ***SE*** | ***P*** |  | ***M*** | ***SE*** | ***P*** |  | ***M*** | ***SE*** | ***P*** |  | ***M*** | ***SE*** | ***P*** |  | ***M*** | ***SE*** | ***P*** |
| **Post-test score** |  |  |  |  |  |  |  |  |  |  |  |  |  |  |  |  |  |  |  |
| Intercept | 52.75 | 1.94 | 1.00 |  | 52.75 | 1.94 | 1.00 |  | 50.40 | 2.41 | 1.00 |  | 50.43 | 2.41 | 1.00 |  | 50.48 | 2.56 | 1.00 |
| P-I | 0.13 | 0.17 | 0.11 |  | 0.12 | 0.15 | 0.12 |  | 0.13 | 0.15 | 0.12 |  | 0.00 | 0.18 | 0.02 |  | -0.00 | 0.21 | 0.02 |
| P-II | 0.01 | 0.18 | 0.05 |  | 0.01 | 0.15 | 0.04 |  | 0.01 | 0.15 | 0.04 |  | 0.00 | 0.19 | 0.02 |  | 0.00 | 0.22 | 0.03 |
| Pre-test score |  |  |  |  | 0.60 | 0.15 | 0.97 |  | 0.60 | 0.15 | 0.97 |  | 0.59 | 0.16 | 0.96 |  |  |  |  |
| Group |  |  |  |  |  |  |  |  | 4.70 | 3.65 | 0.38 |  | 4.70 | 3.65 | 0.40 |  | 4.60 | 4.03 | 0.30 |
| P-I x Group |  |  |  |  |  |  |  |  |  |  |  |  | 0.25 | 0.31 | 0.11 |  | 0.28 | 0.36 | 0.12 |
| P-II x Group |  |  |  |  |  |  |  |  |  |  |  |  | -0.00 | 0.30 | 0.05 |  | -0.00 | 0.35 | 0.05 |
| **Absolute change score** |  |  |  |  |  |  |  |  |  |  |  |  |  |  |  |  |  |  |  |
| Intercept | 2.77 | 1.85 | 0.42 |  | 2.77 | 1.85 | 0.48 |  | 0.43 | 2.34 | 0.09 |  | 0.45 | 2.34 | 0.09 |  | 0.42 | 2.39 | 0.07 |
| P-I | 0.11 | 0.16 | 0.10 |  | 0.12 | 0.15 | 0.12 |  | 0.13 | 0.15 | 0.12 |  | 0.00 | 0.18 | 0.02 |  | 0.01 | 0.20 | 0.03 |
| P-II | 0.01 | 0.16 | 0.04 |  | 0.01 | 0.15 | 0.04 |  | 0.01 | 0.15 | 0.04 |  | 0.00 | 0.19 | 0.02 |  | 0.01 | 0.21 | 0.04 |
| Pre-test score |  |  |  |  | -0.40 | 0.15 | 0.72 |  | -0.40 | 0.15 | 0.75 |  | -0.41 | 0.16 | 0.73 |  |  |  |  |
| Group |  |  |  |  |  |  |  |  | 4.70 | 3.65 | 0.38 |  | 4.70 | 3.65 | 0.40 |  | 4.75 | 3.78 | 0.36 |
| P-I x Group |  |  |  |  |  |  |  |  |  |  |  |  | 0.25 | 0.31 | 0.11 |  | 0.23 | 0.32 | 0.10 |
| P-II x Group |  |  |  |  |  |  |  |  |  |  |  |  | -0.00 | 0.30 | 0.05 |  | -0.01 | 0.32 | 0.05 |
| **Relative change score** |  |  |  |  |  |  |  |  |  |  |  |  |  |  |  |  |  |  |  |
| Intercept | 7.71 | 4.05 | 0.57 |  | 7.71 | 4.05 | 0.63 |  | 3.10 | 5.11 | 0.14 |  | 3.15 | 5.11 | 0.14 |  | 3.08 | 5.26 | 0.09 |
| P-I | 0.23 | 0.36 | 0.08 |  | 0.25 | 0.32 | 0.12 |  | 0.26 | 0.32 | 0.12 |  | 0.01 | 0.39 | 0.02 |  | 0.02 | 0.45 | 0.03 |
| P-II | 0.01 | 0.36 | 0.05 |  | 0.00 | 0.33 | 0.05 |  | 0.00 | 0.32 | 0.04 |  | 0.00 | 0.42 | 0.03 |  | 0.01 | 0.47 | 0.04 |
| Pre-test score |  |  |  |  | -1.06 | 0.40 | 0.83 |  | -1.06 | 0.40 | 0.84 |  | -1.07 | 0.41 | 0.83 |  |  |  |  |
| Group |  |  |  |  |  |  |  |  | 9.22 | 7.81 | 0.35 |  | 9.22 | 7.83 | 0.35 |  | 9.35 | 8.32 | 0.30 |
| P-I x Group |  |  |  |  |  |  |  |  |  |  |  |  | 0.52 | 0.66 | 0.10 |  | 0.46 | 0.72 | 0.08 |
| P-II x Group |  |  |  |  |  |  |  |  |  |  |  |  | -0.01 | 0.67 | 0.05 |  | -0.01 | 0.74 | 0.04 |
| **Residual score** |  |  |  |  |  |  |  |  |  |  |  |  |  |  |  |  |  |  |  |
| Intercept | -0.00 | 0.00 | 0.00 |  | -0.00 | 0.00 | 0.00 |  | -2.35 | 1.82 | 0.17 |  | -2.32 | 1.82 | 0.18 |  | -2.27 | 1.79 | 0.17 |
| P-I | 0.12 | 0.15 | 0.12 |  | 0.12 | 0.15 | 0.12 |  | 0.13 | 0.15 | 0.12 |  | 0.00 | 0.18 | 0.02 |  | 0.00 | 0.18 | 0.02 |
| P-II | 0.01 | 0.15 | 0.04 |  | 0.01 | 0.15 | 0.04 |  | 0.01 | 0.15 | 0.04 |  | 0.00 | 0.19 | 0.02 |  | 0.00 | 0.19 | 0.02 |
| Pre-test score |  |  |  |  | -0.00 | 0.04 | 0.00 |  | -0.00 | 0.06 | 0.00 |  | -0.01 | 0.07 | 0.00 |  |  |  |  |
| Group |  |  |  |  |  |  |  |  | 4.70 | 3.65 | 0.38 |  | 4.70 | 3.65 | 0.40 |  | 4.59 | 3.57 | 0.39 |
| P-I x Group |  |  |  |  |  |  |  |  |  |  |  |  | 0.25 | 0.31 | 0.11 |  | 0.24 | 0.30 | 0.10 |
| P-II x Group |  |  |  |  |  |  |  |  |  |  |  |  | -0.00 | 0.30 | 0.05 |  | -0.00 | 0.30 | 0.05 |

Table 2: Results of all investigated simulations ordered according to their used model and dependent variable, for a **reliability of .60**, and a sample size of ***n* = 100**

| **Coefficient** | **Model 1** | | |  | **Model 2** | | |  | **Model 3** | | |  | **Model 4** | | |  | **Model 5** | | |
| --- | --- | --- | --- | --- | --- | --- | --- | --- | --- | --- | --- | --- | --- | --- | --- | --- | --- | --- | --- |
|  | ***M*** | ***SE*** | ***P*** |  | ***M*** | ***SE*** | ***P*** |  | ***M*** | ***SE*** | ***P*** |  | ***M*** | ***SE*** | ***P*** |  | ***M*** | ***SE*** | ***P*** |
| **Post-test score** |  |  |  |  |  |  |  |  |  |  |  |  |  |  |  |  |  |  |  |
| Intercept | 52.85 | 1.35 | 1.00 |  | 52.85 | 1.35 | 1.00 |  | 50.39 | 1.62 | 1.00 |  | 50.38 | 1.62 | 1.00 |  | 50.40 | 1.75 | 1.00 |
| P-I | 0.14 | 0.12 | 0.20 |  | 0.12 | 0.10 | 0.18 |  | 0.12 | 0.10 | 0.20 |  | 0.00 | 0.13 | 0.03 |  | 0.00 | 0.15 | 0.03 |
| P-II | 0.01 | 0.11 | 0.04 |  | 0.01 | 0.10 | 0.04 |  | 0.01 | 0.10 | 0.04 |  | 0.01 | 0.13 | 0.02 |  | 0.01 | 0.15 | 0.03 |
| Pre-test score |  |  |  |  | 0.60 | 0.10 | 1.00 |  | 0.60 | 0.10 | 1.00 |  | 0.59 | 0.10 | 1.00 |  |  |  |  |
| Group |  |  |  |  |  |  |  |  | 4.91 | 2.55 | 0.63 |  | 4.90 | 2.56 | 0.64 |  | 4.89 | 2.86 | 0.52 |
| P-I x Group |  |  |  |  |  |  |  |  |  |  |  |  | 0.23 | 0.20 | 0.22 |  | 0.27 | 0.24 | 0.21 |
| P-II x Group |  |  |  |  |  |  |  |  |  |  |  |  | 0.00 | 0.20 | 0.04 |  | -0.00 | 0.23 | 0.04 |
| **Absolute change score** |  |  |  |  |  |  |  |  |  |  |  |  |  |  |  |  |  |  |  |
| Intercept | 2.87 | 1.29 | 0.71 |  | 2.87 | 1.29 | 0.76 |  | 0.42 | 1.60 | 0.11 |  | 0.41 | 1.60 | 0.11 |  | 0.39 | 1.65 | 0.10 |
| P-I | 0.11 | 0.11 | 0.16 |  | 0.12 | 0.10 | 0.18 |  | 0.12 | 0.10 | 0.20 |  | 0.00 | 0.13 | 0.03 |  | 0.01 | 0.15 | 0.03 |
| P-II | 0.01 | 0.11 | 0.04 |  | 0.01 | 0.10 | 0.04 |  | 0.01 | 0.10 | 0.04 |  | 0.01 | 0.13 | 0.02 |  | 0.01 | 0.15 | 0.03 |
| Pre-test score |  |  |  |  | -0.40 | 0.10 | 0.96 |  | -0.40 | 0.10 | 0.97 |  | -0.41 | 0.10 | 0.97 |  |  |  |  |
| Group |  |  |  |  |  |  |  |  | 4.91 | 2.55 | 0.63 |  | 4.90 | 2.56 | 0.64 |  | 4.92 | 2.63 | 0.60 |
| P-I x Group |  |  |  |  |  |  |  |  |  |  |  |  | 0.23 | 0.20 | 0.22 |  | 0.21 | 0.22 | 0.16 |
| P-II x Group |  |  |  |  |  |  |  |  |  |  |  |  | 0.00 | 0.20 | 0.04 |  | 0.00 | 0.22 | 0.05 |
| **Relative change score** |  |  |  |  |  |  |  |  |  |  |  |  |  |  |  |  |  |  |  |
| Intercept | 7.89 | 2.89 | 0.88 |  | 7.89 | 2.89 | 0.89 |  | 3.12 | 3.51 | 0.21 |  | 3.10 | 3.51 | 0.20 |  | 3.07 | 3.67 | 0.15 |
| P-I | 0.23 | 0.25 | 0.14 |  | 0.25 | 0.22 | 0.19 |  | 0.25 | 0.22 | 0.19 |  | 0.01 | 0.28 | 0.03 |  | 0.01 | 0.33 | 0.03 |
| P-II | 0.02 | 0.26 | 0.04 |  | 0.02 | 0.22 | 0.04 |  | 0.02 | 0.22 | 0.04 |  | 0.01 | 0.28 | 0.03 |  | 0.01 | 0.33 | 0.04 |
| Pre-test score |  |  |  |  | -1.07 | 0.31 | 0.98 |  | -1.07 | 0.31 | 0.99 |  | -1.07 | 0.31 | 0.99 |  |  |  |  |
| Group |  |  |  |  |  |  |  |  | 9.56 | 5.52 | 0.54 |  | 9.55 | 5.53 | 0.55 |  | 9.58 | 5.85 | 0.50 |
| P-I x Group |  |  |  |  |  |  |  |  |  |  |  |  | 0.49 | 0.44 | 0.19 |  | 0.43 | 0.50 | 0.13 |
| P-II x Group |  |  |  |  |  |  |  |  |  |  |  |  | 0.00 | 0.44 | 0.04 |  | 0.01 | 0.50 | 0.06 |
| **Residual score** |  |  |  |  |  |  |  |  |  |  |  |  |  |  |  |  |  |  |  |
| Intercept | 0.00 | 0.00 | 0.00 |  | 0.00 | 0.00 | 0.00 |  | -2.45 | 1.28 | 0.38 |  | -2.46 | 1.28 | 0.38 |  | -2.44 | 1.26 | 0.38 |
| P-I | 0.12 | 0.10 | 0.18 |  | 0.12 | 0.10 | 0.18 |  | 0.12 | 0.10 | 0.20 |  | 0.00 | 0.13 | 0.03 |  | 0.00 | 0.13 | 0.02 |
| P-II | 0.01 | 0.10 | 0.04 |  | 0.01 | 0.10 | 0.04 |  | 0.01 | 0.10 | 0.04 |  | 0.01 | 0.13 | 0.02 |  | 0.01 | 0.13 | 0.02 |
| Pre-test score |  |  |  |  | -0.00 | 0.02 | 0.00 |  | -0.00 | 0.04 | 0.00 |  | -0.01 | 0.04 | 0.00 |  |  |  |  |
| Group |  |  |  |  |  |  |  |  | 4.91 | 2.55 | 0.63 |  | 4.90 | 2.56 | 0.64 |  | 4.85 | 2.53 | 0.64 |
| P-I x Group |  |  |  |  |  |  |  |  |  |  |  |  | 0.23 | 0.20 | 0.22 |  | 0.23 | 0.20 | 0.22 |
| P-II x Group |  |  |  |  |  |  |  |  |  |  |  |  | 0.00 | 0.20 | 0.04 |  | 0.00 | 0.20 | 0.04 |

Table 3: Results of all investigated simulations ordered according to their used model and dependent variable, for a **reliability of .60**, and a sample size of ***n* = 150**

| **Coefficient** | **Model 1** | | |  | **Model 2** | | |  | **Model 3** | | |  | **Model 4** | | |  | **Model 5** | | |
| --- | --- | --- | --- | --- | --- | --- | --- | --- | --- | --- | --- | --- | --- | --- | --- | --- | --- | --- | --- |
|  | ***M*** | ***SE*** | ***P*** |  | ***M*** | ***SE*** | ***P*** |  | ***M*** | ***SE*** | ***P*** |  | ***M*** | ***SE*** | ***P*** |  | ***M*** | ***SE*** | ***P*** |
| **Post-test score** |  |  |  |  |  |  |  |  |  |  |  |  |  |  |  |  |  |  |  |
| Intercept | 52.84 | 1.13 | 1.00 |  | 52.84 | 1.13 | 1.00 |  | 50.42 | 1.38 | 1.00 |  | 50.42 | 1.38 | 1.00 |  | 50.44 | 1.47 | 1.00 |
| P-I | 0.13 | 0.10 | 0.26 |  | 0.12 | 0.09 | 0.26 |  | 0.12 | 0.08 | 0.29 |  | 0.00 | 0.10 | 0.03 |  | 0.01 | 0.12 | 0.02 |
| P-II | 0.00 | 0.10 | 0.05 |  | 0.00 | 0.08 | 0.04 |  | 0.00 | 0.08 | 0.05 |  | 0.00 | 0.10 | 0.03 |  | 0.00 | 0.12 | 0.03 |
| Pre-test score |  |  |  |  | 0.60 | 0.08 | 1.00 |  | 0.60 | 0.08 | 1.00 |  | 0.59 | 0.08 | 1.00 |  |  |  |  |
| Group |  |  |  |  |  |  |  |  | 4.85 | 2.13 | 0.78 |  | 4.84 | 2.13 | 0.78 |  | 4.80 | 2.38 | 0.68 |
| P-I x Group |  |  |  |  |  |  |  |  |  |  |  |  | 0.23 | 0.16 | 0.29 |  | 0.26 | 0.19 | 0.27 |
| P-II x Group |  |  |  |  |  |  |  |  |  |  |  |  | 0.00 | 0.16 | 0.04 |  | 0.00 | 0.19 | 0.04 |
| **Absolute change score** |  |  |  |  |  |  |  |  |  |  |  |  |  |  |  |  |  |  |  |
| Intercept | 2.86 | 1.06 | 0.86 |  | 2.86 | 1.06 | 0.88 |  | 0.44 | 1.34 | 0.11 |  | 0.43 | 1.34 | 0.12 |  | 0.42 | 1.36 | 0.09 |
| P-I | 0.10 | 0.09 | 0.21 |  | 0.12 | 0.09 | 0.26 |  | 0.12 | 0.08 | 0.29 |  | 0.00 | 0.10 | 0.03 |  | -0.00 | 0.12 | 0.04 |
| P-II | 0.00 | 0.09 | 0.05 |  | 0.00 | 0.08 | 0.04 |  | 0.00 | 0.08 | 0.05 |  | 0.00 | 0.10 | 0.03 |  | -0.00 | 0.12 | 0.04 |
| Pre-test score |  |  |  |  | -0.40 | 0.08 | 1.00 |  | -0.40 | 0.08 | 1.00 |  | -0.41 | 0.08 | 1.00 |  |  |  |  |
| Group |  |  |  |  |  |  |  |  | 4.85 | 2.13 | 0.78 |  | 4.84 | 2.13 | 0.78 |  | 4.87 | 2.18 | 0.74 |
| P-I x Group |  |  |  |  |  |  |  |  |  |  |  |  | 0.23 | 0.16 | 0.29 |  | 0.21 | 0.18 | 0.23 |
| P-II x Group |  |  |  |  |  |  |  |  |  |  |  |  | 0.00 | 0.16 | 0.04 |  | 0.01 | 0.17 | 0.04 |
| **Relative change score** |  |  |  |  |  |  |  |  |  |  |  |  |  |  |  |  |  |  |  |
| Intercept | 7.84 | 2.38 | 0.96 |  | 7.84 | 2.38 | 0.97 |  | 3.12 | 2.90 | 0.25 |  | 3.11 | 2.90 | 0.25 |  | 3.08 | 2.96 | 0.20 |
| P-I | 0.21 | 0.21 | 0.19 |  | 0.24 | 0.18 | 0.26 |  | 0.24 | 0.18 | 0.27 |  | 0.01 | 0.22 | 0.03 |  | 0.00 | 0.27 | 0.05 |
| P-II | 0.00 | 0.20 | 0.04 |  | 0.00 | 0.18 | 0.04 |  | 0.00 | 0.18 | 0.04 |  | 0.00 | 0.22 | 0.02 |  | -0.00 | 0.26 | 0.03 |
| Pre-test score |  |  |  |  | -1.06 | 0.23 | 1.00 |  | -1.06 | 0.23 | 1.00 |  | -1.07 | 0.23 | 1.00 |  |  |  |  |
| Group |  |  |  |  |  |  |  |  | 9.46 | 4.52 | 0.70 |  | 9.45 | 4.53 | 0.71 |  | 9.52 | 4.73 | 0.65 |
| P-I x Group |  |  |  |  |  |  |  |  |  |  |  |  | 0.47 | 0.36 | 0.27 |  | 0.42 | 0.41 | 0.20 |
| P-II x Group |  |  |  |  |  |  |  |  |  |  |  |  | 0.01 | 0.35 | 0.04 |  | 0.01 | 0.39 | 0.04 |
| **Residual score** |  |  |  |  |  |  |  |  |  |  |  |  |  |  |  |  |  |  |  |
| Intercept | 0.00 | 0.00 | 0.00 |  | 0.00 | 0.00 | 0.00 |  | -2.42 | 1.06 | 0.57 |  | -2.43 | 1.06 | 0.58 |  | -2.41 | 1.05 | 0.58 |
| P-I | 0.11 | 0.08 | 0.26 |  | 0.12 | 0.09 | 0.26 |  | 0.12 | 0.08 | 0.29 |  | 0.00 | 0.10 | 0.03 |  | 0.00 | 0.10 | 0.03 |
| P-II | 0.00 | 0.08 | 0.04 |  | 0.00 | 0.08 | 0.04 |  | 0.00 | 0.08 | 0.05 |  | 0.00 | 0.10 | 0.03 |  | 0.00 | 0.10 | 0.03 |
| Pre-test score |  |  |  |  | -0.00 | 0.01 | 0.00 |  | -0.00 | 0.03 | 0.00 |  | -0.01 | 0.03 | 0.00 |  |  |  |  |
| Group |  |  |  |  |  |  |  |  | 4.85 | 2.13 | 0.78 |  | 4.84 | 2.13 | 0.78 |  | 4.81 | 2.11 | 0.78 |
| P-I x Group |  |  |  |  |  |  |  |  |  |  |  |  | 0.23 | 0.16 | 0.29 |  | 0.23 | 0.16 | 0.29 |
| P-II x Group |  |  |  |  |  |  |  |  |  |  |  |  | 0.00 | 0.16 | 0.04 |  | 0.00 | 0.16 | 0.04 |

Table 4: Results of all investigated simulations ordered according to their used model and dependent variable, for a **reliability of .60**, and a sample size of ***n* = 200**

| **Coefficient** | **Model 1** | | |  | **Model 2** | | |  | **Model 3** | | |  | **Model 4** | | |  | **Model 5** | | |
| --- | --- | --- | --- | --- | --- | --- | --- | --- | --- | --- | --- | --- | --- | --- | --- | --- | --- | --- | --- |
|  | ***M*** | ***SE*** | ***P*** |  | ***M*** | ***SE*** | ***P*** |  | ***M*** | ***SE*** | ***P*** |  | ***M*** | ***SE*** | ***P*** |  | ***M*** | ***SE*** | ***P*** |
| **Post-test score** |  |  |  |  |  |  |  |  |  |  |  |  |  |  |  |  |  |  |  |
| Intercept | 52.81 | 0.99 | 1.00 |  | 52.81 | 0.99 | 1.00 |  | 50.36 | 1.24 | 1.00 |  | 50.36 | 1.23 | 1.00 |  | 50.37 | 1.31 | 1.00 |
| P-I | 0.13 | 0.09 | 0.35 |  | 0.12 | 0.07 | 0.34 |  | 0.12 | 0.07 | 0.36 |  | -0.00 | 0.09 | 0.03 |  | -0.00 | 0.10 | 0.02 |
| P-II | 0.00 | 0.09 | 0.06 |  | 0.00 | 0.07 | 0.06 |  | 0.00 | 0.07 | 0.06 |  | 0.00 | 0.09 | 0.03 |  | 0.00 | 0.10 | 0.03 |
| Pre-test score |  |  |  |  | 0.60 | 0.07 | 1.00 |  | 0.60 | 0.07 | 1.00 |  | 0.59 | 0.07 | 1.00 |  |  |  |  |
| Group |  |  |  |  |  |  |  |  | 4.91 | 1.83 | 0.87 |  | 4.90 | 1.83 | 0.87 |  | 4.88 | 2.03 | 0.80 |
| P-I x Group |  |  |  |  |  |  |  |  |  |  |  |  | 0.24 | 0.14 | 0.39 |  | 0.28 | 0.17 | 0.40 |
| P-II x Group |  |  |  |  |  |  |  |  |  |  |  |  | 0.00 | 0.14 | 0.06 |  | -0.00 | 0.16 | 0.05 |
| **Absolute change score** |  |  |  |  |  |  |  |  |  |  |  |  |  |  |  |  |  |  |  |
| Intercept | 2.83 | 0.90 | 0.93 |  | 2.83 | 0.90 | 0.94 |  | 0.37 | 1.20 | 0.12 |  | 0.38 | 1.20 | 0.13 |  | 0.37 | 1.23 | 0.10 |
| P-I | 0.10 | 0.08 | 0.25 |  | 0.12 | 0.07 | 0.34 |  | 0.12 | 0.07 | 0.36 |  | -0.00 | 0.09 | 0.03 |  | -0.00 | 0.10 | 0.03 |
| P-II | 0.00 | 0.08 | 0.04 |  | 0.00 | 0.07 | 0.06 |  | 0.00 | 0.07 | 0.06 |  | 0.00 | 0.09 | 0.03 |  | -0.00 | 0.10 | 0.03 |
| Pre-test score |  |  |  |  | -0.40 | 0.07 | 1.00 |  | -0.40 | 0.07 | 1.00 |  | -0.41 | 0.07 | 1.00 |  |  |  |  |
| Group |  |  |  |  |  |  |  |  | 4.91 | 1.83 | 0.87 |  | 4.90 | 1.83 | 0.87 |  | 4.92 | 1.93 | 0.84 |
| P-I x Group |  |  |  |  |  |  |  |  |  |  |  |  | 0.24 | 0.14 | 0.39 |  | 0.21 | 0.15 | 0.29 |
| P-II x Group |  |  |  |  |  |  |  |  |  |  |  |  | 0.00 | 0.14 | 0.06 |  | 0.01 | 0.15 | 0.06 |
| **Relative change score** |  |  |  |  |  |  |  |  |  |  |  |  |  |  |  |  |  |  |  |
| Intercept | 7.78 | 2.02 | 0.99 |  | 7.78 | 2.02 | 0.99 |  | 2.98 | 2.66 | 0.30 |  | 2.99 | 2.65 | 0.31 |  | 2.96 | 2.73 | 0.24 |
| P-I | 0.21 | 0.17 | 0.21 |  | 0.24 | 0.16 | 0.33 |  | 0.24 | 0.16 | 0.35 |  | -0.01 | 0.20 | 0.03 |  | -0.01 | 0.23 | 0.03 |
| P-II | 0.01 | 0.18 | 0.05 |  | 0.00 | 0.16 | 0.06 |  | 0.01 | 0.16 | 0.06 |  | 0.00 | 0.19 | 0.03 |  | 0.00 | 0.23 | 0.03 |
| Pre-test score |  |  |  |  | -1.06 | 0.24 | 1.00 |  | -1.06 | 0.23 | 1.00 |  | -1.07 | 0.23 | 1.00 |  |  |  |  |
| Group |  |  |  |  |  |  |  |  | 9.60 | 4.03 | 0.81 |  | 9.60 | 4.03 | 0.81 |  | 9.64 | 4.30 | 0.75 |
| P-I x Group |  |  |  |  |  |  |  |  |  |  |  |  | 0.50 | 0.32 | 0.37 |  | 0.44 | 0.35 | 0.24 |
| P-II x Group |  |  |  |  |  |  |  |  |  |  |  |  | 0.01 | 0.31 | 0.05 |  | 0.01 | 0.35 | 0.05 |
| **Residual score** |  |  |  |  |  |  |  |  |  |  |  |  |  |  |  |  |  |  |  |
| Intercept | 0.00 | 0.00 | 0.00 |  | 0.00 | 0.00 | 0.00 |  | -2.45 | 0.92 | 0.71 |  | -2.45 | 0.91 | 0.72 |  | -2.44 | 0.91 | 0.71 |
| P-I | 0.11 | 0.07 | 0.34 |  | 0.12 | 0.07 | 0.34 |  | 0.12 | 0.07 | 0.36 |  | -0.00 | 0.09 | 0.03 |  | -0.00 | 0.09 | 0.03 |
| P-II | 0.00 | 0.07 | 0.06 |  | 0.00 | 0.07 | 0.06 |  | 0.00 | 0.07 | 0.06 |  | 0.00 | 0.09 | 0.03 |  | 0.00 | 0.09 | 0.03 |
| Pre-test score |  |  |  |  | -0.00 | 0.01 | 0.00 |  | -0.00 | 0.02 | 0.00 |  | -0.01 | 0.02 | 0.00 |  |  |  |  |
| Group |  |  |  |  |  |  |  |  | 4.91 | 1.83 | 0.87 |  | 4.90 | 1.83 | 0.87 |  | 4.88 | 1.83 | 0.87 |
| P-I x Group |  |  |  |  |  |  |  |  |  |  |  |  | 0.24 | 0.14 | 0.39 |  | 0.24 | 0.14 | 0.39 |
| P-II x Group |  |  |  |  |  |  |  |  |  |  |  |  | 0.00 | 0.14 | 0.06 |  | 0.00 | 0.14 | 0.06 |

Table 5: Results of all investigated simulations ordered according to their used model and dependent variable, for a **reliability of .60**, and a sample size of ***n* = 250**

| **Coefficient** | **Model 1** | | |  | **Model 2** | | |  | **Model 3** | | |  | **Model 4** | | |  | **Model 5** | | |
| --- | --- | --- | --- | --- | --- | --- | --- | --- | --- | --- | --- | --- | --- | --- | --- | --- | --- | --- | --- |
|  | ***M*** | ***SE*** | ***P*** |  | ***M*** | ***SE*** | ***P*** |  | ***M*** | ***SE*** | ***P*** |  | ***M*** | ***SE*** | ***P*** |  | ***M*** | ***SE*** | ***P*** |
| **Post-test score** |  |  |  |  |  |  |  |  |  |  |  |  |  |  |  |  |  |  |  |
| Intercept | 52.84 | 0.90 | 1.00 |  | 52.84 | 0.90 | 1.00 |  | 50.43 | 1.04 | 1.00 |  | 50.43 | 1.04 | 1.00 |  | 50.43 | 1.10 | 1.00 |
| P-I | 0.14 | 0.08 | 0.42 |  | 0.12 | 0.06 | 0.44 |  | 0.12 | 0.06 | 0.46 |  | 0.00 | 0.08 | 0.03 |  | 0.00 | 0.09 | 0.02 |
| P-II | 0.00 | 0.08 | 0.05 |  | 0.00 | 0.06 | 0.05 |  | 0.00 | 0.06 | 0.05 |  | 0.00 | 0.08 | 0.02 |  | 0.00 | 0.09 | 0.03 |
| Pre-test score |  |  |  |  | 0.60 | 0.06 | 1.00 |  | 0.60 | 0.06 | 1.00 |  | 0.60 | 0.06 | 1.00 |  |  |  |  |
| Group |  |  |  |  |  |  |  |  | 4.83 | 1.59 | 0.93 |  | 4.83 | 1.59 | 0.93 |  | 4.83 | 1.77 | 0.88 |
| P-I x Group |  |  |  |  |  |  |  |  |  |  |  |  | 0.23 | 0.13 | 0.45 |  | 0.27 | 0.15 | 0.45 |
| P-II x Group |  |  |  |  |  |  |  |  |  |  |  |  | -0.00 | 0.12 | 0.04 |  | -0.00 | 0.15 | 0.05 |
| **Absolute change score** |  |  |  |  |  |  |  |  |  |  |  |  |  |  |  |  |  |  |  |
| Intercept | 2.85 | 0.84 | 0.95 |  | 2.85 | 0.84 | 0.97 |  | 0.44 | 0.98 | 0.11 |  | 0.44 | 0.98 | 0.11 |  | 0.44 | 1.02 | 0.09 |
| P-I | 0.11 | 0.07 | 0.33 |  | 0.12 | 0.06 | 0.44 |  | 0.12 | 0.06 | 0.46 |  | 0.00 | 0.08 | 0.03 |  | 0.00 | 0.09 | 0.03 |
| P-II | 0.00 | 0.07 | 0.05 |  | 0.00 | 0.06 | 0.05 |  | 0.00 | 0.06 | 0.05 |  | 0.00 | 0.08 | 0.02 |  | 0.00 | 0.09 | 0.03 |
| Pre-test score |  |  |  |  | -0.40 | 0.06 | 1.00 |  | -0.40 | 0.06 | 1.00 |  | -0.40 | 0.06 | 1.00 |  |  |  |  |
| Group |  |  |  |  |  |  |  |  | 4.83 | 1.59 | 0.93 |  | 4.83 | 1.59 | 0.93 |  | 4.82 | 1.68 | 0.91 |
| P-I x Group |  |  |  |  |  |  |  |  |  |  |  |  | 0.23 | 0.13 | 0.45 |  | 0.21 | 0.13 | 0.31 |
| P-II x Group |  |  |  |  |  |  |  |  |  |  |  |  | -0.00 | 0.12 | 0.04 |  | -0.00 | 0.13 | 0.04 |
| **Relative change score** |  |  |  |  |  |  |  |  |  |  |  |  |  |  |  |  |  |  |  |
| Intercept | 7.82 | 1.85 | 0.99 |  | 7.82 | 1.85 | 1.00 |  | 3.15 | 2.16 | 0.38 |  | 3.15 | 2.16 | 0.38 |  | 3.16 | 2.28 | 0.32 |
| P-I | 0.21 | 0.16 | 0.28 |  | 0.24 | 0.14 | 0.40 |  | 0.24 | 0.14 | 0.41 |  | 0.01 | 0.17 | 0.02 |  | 0.01 | 0.20 | 0.03 |
| P-II | 0.00 | 0.15 | 0.05 |  | 0.00 | 0.14 | 0.05 |  | 0.00 | 0.13 | 0.04 |  | 0.00 | 0.17 | 0.02 |  | 0.00 | 0.20 | 0.03 |
| Pre-test score |  |  |  |  | -1.06 | 0.18 | 1.00 |  | -1.06 | 0.17 | 1.00 |  | -1.07 | 0.17 | 1.00 |  |  |  |  |
| Group |  |  |  |  |  |  |  |  | 9.35 | 3.41 | 0.88 |  | 9.35 | 3.41 | 0.88 |  | 9.32 | 3.69 | 0.82 |
| P-I x Group |  |  |  |  |  |  |  |  |  |  |  |  | 0.48 | 0.28 | 0.40 |  | 0.41 | 0.30 | 0.26 |
| P-II x Group |  |  |  |  |  |  |  |  |  |  |  |  | -0.00 | 0.27 | 0.05 |  | -0.00 | 0.30 | 0.04 |
| **Residual score** |  |  |  |  |  |  |  |  |  |  |  |  |  |  |  |  |  |  |  |
| Intercept | -0.00 | 0.00 | 0.00 |  | -0.00 | 0.00 | 0.00 |  | -2.41 | 0.80 | 0.80 |  | -2.41 | 0.79 | 0.80 |  | -2.40 | 0.79 | 0.81 |
| P-I | 0.12 | 0.06 | 0.44 |  | 0.12 | 0.06 | 0.44 |  | 0.12 | 0.06 | 0.46 |  | 0.00 | 0.08 | 0.03 |  | 0.00 | 0.08 | 0.03 |
| P-II | 0.00 | 0.06 | 0.05 |  | 0.00 | 0.06 | 0.05 |  | 0.00 | 0.06 | 0.05 |  | 0.00 | 0.08 | 0.02 |  | 0.00 | 0.08 | 0.02 |
| Pre-test score |  |  |  |  | -0.00 | 0.01 | 0.00 |  | -0.00 | 0.02 | 0.00 |  | -0.01 | 0.02 | 0.00 |  |  |  |  |
| Group |  |  |  |  |  |  |  |  | 4.83 | 1.59 | 0.93 |  | 4.83 | 1.59 | 0.93 |  | 4.81 | 1.59 | 0.93 |
| P-I x Group |  |  |  |  |  |  |  |  |  |  |  |  | 0.23 | 0.13 | 0.45 |  | 0.23 | 0.12 | 0.45 |
| P-II x Group |  |  |  |  |  |  |  |  |  |  |  |  | -0.00 | 0.12 | 0.04 |  | -0.00 | 0.12 | 0.04 |

Table 6: Results of all investigated simulations ordered according to their used model and dependent variable, for a **reliability of .60**, and a sample size of ***n* = 300**

| **Coefficient** | **Model 1** | | |  | **Model 2** | | |  | **Model 3** | | |  | **Model 4** | | |  | **Model 5** | | |
| --- | --- | --- | --- | --- | --- | --- | --- | --- | --- | --- | --- | --- | --- | --- | --- | --- | --- | --- | --- |
|  | ***M*** | ***SE*** | ***P*** |  | ***M*** | ***SE*** | ***P*** |  | ***M*** | ***SE*** | ***P*** |  | ***M*** | ***SE*** | ***P*** |  | ***M*** | ***SE*** | ***P*** |
| **Post-test score** |  |  |  |  |  |  |  |  |  |  |  |  |  |  |  |  |  |  |  |
| Intercept | 52.85 | 0.82 | 1.00 |  | 52.85 | 0.82 | 1.00 |  | 50.44 | 1.00 | 1.00 |  | 50.44 | 1.00 | 1.00 |  | 50.43 | 1.05 | 1.00 |
| P-I | 0.14 | 0.07 | 0.53 |  | 0.12 | 0.06 | 0.52 |  | 0.12 | 0.06 | 0.54 |  | 0.00 | 0.07 | 0.02 |  | 0.00 | 0.08 | 0.02 |
| P-II | 0.00 | 0.07 | 0.04 |  | -0.00 | 0.06 | 0.05 |  | -0.00 | 0.06 | 0.04 |  | -0.00 | 0.07 | 0.03 |  | -0.00 | 0.08 | 0.02 |
| Pre-test score |  |  |  |  | 0.60 | 0.06 | 1.00 |  | 0.60 | 0.06 | 1.00 |  | 0.60 | 0.06 | 1.00 |  |  |  |  |
| Group |  |  |  |  |  |  |  |  | 4.81 | 1.50 | 0.95 |  | 4.81 | 1.50 | 0.95 |  | 4.83 | 1.64 | 0.92 |
| P-I x Group |  |  |  |  |  |  |  |  |  |  |  |  | 0.23 | 0.11 | 0.53 |  | 0.28 | 0.13 | 0.54 |
| P-II x Group |  |  |  |  |  |  |  |  |  |  |  |  | 0.00 | 0.12 | 0.05 |  | 0.00 | 0.13 | 0.05 |
| **Absolute change score** |  |  |  |  |  |  |  |  |  |  |  |  |  |  |  |  |  |  |  |
| Intercept | 2.86 | 0.78 | 0.98 |  | 2.86 | 0.78 | 0.99 |  | 0.45 | 0.97 | 0.14 |  | 0.45 | 0.97 | 0.14 |  | 0.46 | 1.00 | 0.13 |
| P-I | 0.11 | 0.06 | 0.39 |  | 0.12 | 0.06 | 0.52 |  | 0.12 | 0.06 | 0.54 |  | 0.00 | 0.07 | 0.02 |  | 0.00 | 0.08 | 0.03 |
| P-II | -0.00 | 0.06 | 0.04 |  | -0.00 | 0.06 | 0.05 |  | -0.00 | 0.06 | 0.04 |  | -0.00 | 0.07 | 0.03 |  | -0.00 | 0.08 | 0.03 |
| Pre-test score |  |  |  |  | -0.40 | 0.06 | 1.00 |  | -0.40 | 0.06 | 1.00 |  | -0.40 | 0.06 | 1.00 |  |  |  |  |
| Group |  |  |  |  |  |  |  |  | 4.81 | 1.50 | 0.95 |  | 4.81 | 1.50 | 0.95 |  | 4.80 | 1.58 | 0.94 |
| P-I x Group |  |  |  |  |  |  |  |  |  |  |  |  | 0.23 | 0.11 | 0.53 |  | 0.21 | 0.12 | 0.39 |
| P-II x Group |  |  |  |  |  |  |  |  |  |  |  |  | 0.00 | 0.12 | 0.05 |  | 0.00 | 0.13 | 0.05 |
| **Relative change score** |  |  |  |  |  |  |  |  |  |  |  |  |  |  |  |  |  |  |  |
| Intercept | 7.81 | 1.72 | 1.00 |  | 7.81 | 1.72 | 1.00 |  | 3.15 | 2.14 | 0.45 |  | 3.15 | 2.14 | 0.46 |  | 3.16 | 2.22 | 0.37 |
| P-I | 0.21 | 0.14 | 0.32 |  | 0.25 | 0.13 | 0.49 |  | 0.25 | 0.12 | 0.51 |  | 0.01 | 0.15 | 0.02 |  | 0.01 | 0.18 | 0.03 |
| P-II | -0.01 | 0.14 | 0.04 |  | -0.01 | 0.12 | 0.05 |  | -0.01 | 0.12 | 0.05 |  | -0.01 | 0.15 | 0.03 |  | -0.01 | 0.18 | 0.03 |
| Pre-test score |  |  |  |  | -1.05 | 0.16 | 1.00 |  | -1.05 | 0.16 | 1.00 |  | -1.06 | 0.16 | 1.00 |  |  |  |  |
| Group |  |  |  |  |  |  |  |  | 9.32 | 3.22 | 0.92 |  | 9.32 | 3.22 | 0.92 |  | 9.29 | 3.47 | 0.86 |
| P-I x Group |  |  |  |  |  |  |  |  |  |  |  |  | 0.48 | 0.24 | 0.49 |  | 0.41 | 0.26 | 0.30 |
| P-II x Group |  |  |  |  |  |  |  |  |  |  |  |  | 0.01 | 0.25 | 0.05 |  | 0.01 | 0.28 | 0.06 |
| **Residual score** |  |  |  |  |  |  |  |  |  |  |  |  |  |  |  |  |  |  |  |
| Intercept | -0.00 | 0.00 | 0.00 |  | -0.00 | 0.00 | 0.00 |  | -2.41 | 0.75 | 0.86 |  | -2.41 | 0.75 | 0.87 |  | -2.40 | 0.75 | 0.87 |
| P-I | 0.12 | 0.06 | 0.52 |  | 0.12 | 0.06 | 0.52 |  | 0.12 | 0.06 | 0.54 |  | 0.00 | 0.07 | 0.02 |  | 0.00 | 0.07 | 0.02 |
| P-II | -0.00 | 0.06 | 0.05 |  | -0.00 | 0.06 | 0.05 |  | -0.00 | 0.06 | 0.04 |  | -0.00 | 0.07 | 0.03 |  | -0.00 | 0.07 | 0.03 |
| Pre-test score |  |  |  |  | -0.00 | 0.01 | 0.00 |  | -0.00 | 0.02 | 0.00 |  | -0.01 | 0.02 | 0.00 |  |  |  |  |
| Group |  |  |  |  |  |  |  |  | 4.81 | 1.50 | 0.95 |  | 4.81 | 1.50 | 0.95 |  | 4.80 | 1.50 | 0.95 |
| P-I x Group |  |  |  |  |  |  |  |  |  |  |  |  | 0.23 | 0.11 | 0.53 |  | 0.23 | 0.11 | 0.53 |
| P-II x Group |  |  |  |  |  |  |  |  |  |  |  |  | 0.00 | 0.12 | 0.05 |  | 0.00 | 0.12 | 0.05 |

Table 7: Results of all investigated simulations ordered according to their used model and dependent variable, for a **reliability of .60**, and a sample size of ***n* = 400**

| **Coefficient** | **Model 1** | | |  | **Model 2** | | |  | **Model 3** | | |  | **Model 4** | | |  | **Model 5** | | |
| --- | --- | --- | --- | --- | --- | --- | --- | --- | --- | --- | --- | --- | --- | --- | --- | --- | --- | --- | --- |
|  | ***M*** | ***SE*** | ***P*** |  | ***M*** | ***SE*** | ***P*** |  | ***M*** | ***SE*** | ***P*** |  | ***M*** | ***SE*** | ***P*** |  | ***M*** | ***SE*** | ***P*** |
| **Post-test score** |  |  |  |  |  |  |  |  |  |  |  |  |  |  |  |  |  |  |  |
| Intercept | 52.83 | 0.70 | 1.00 |  | 52.83 | 0.70 | 1.00 |  | 50.43 | 0.84 | 1.00 |  | 50.43 | 0.84 | 1.00 |  | 50.42 | 0.88 | 1.00 |
| P-I | 0.13 | 0.06 | 0.60 |  | 0.12 | 0.05 | 0.61 |  | 0.12 | 0.05 | 0.65 |  | -0.00 | 0.06 | 0.03 |  | -0.00 | 0.07 | 0.03 |
| P-II | 0.00 | 0.06 | 0.05 |  | 0.00 | 0.05 | 0.04 |  | 0.00 | 0.05 | 0.04 |  | 0.00 | 0.06 | 0.04 |  | 0.00 | 0.07 | 0.03 |
| Pre-test score |  |  |  |  | 0.60 | 0.05 | 1.00 |  | 0.60 | 0.05 | 1.00 |  | 0.60 | 0.05 | 1.00 |  |  |  |  |
| Group |  |  |  |  |  |  |  |  | 4.80 | 1.25 | 0.98 |  | 4.80 | 1.24 | 0.98 |  | 4.82 | 1.37 | 0.97 |
| P-I x Group |  |  |  |  |  |  |  |  |  |  |  |  | 0.23 | 0.10 | 0.66 |  | 0.27 | 0.12 | 0.65 |
| P-II x Group |  |  |  |  |  |  |  |  |  |  |  |  | 0.00 | 0.10 | 0.05 |  | 0.00 | 0.11 | 0.05 |
| **Absolute change score** |  |  |  |  |  |  |  |  |  |  |  |  |  |  |  |  |  |  |  |
| Intercept | 2.82 | 0.65 | 0.99 |  | 2.82 | 0.65 | 1.00 |  | 0.42 | 0.82 | 0.15 |  | 0.42 | 0.81 | 0.14 |  | 0.42 | 0.84 | 0.13 |
| P-I | 0.10 | 0.05 | 0.47 |  | 0.12 | 0.05 | 0.61 |  | 0.12 | 0.05 | 0.65 |  | -0.00 | 0.06 | 0.03 |  | 0.00 | 0.07 | 0.04 |
| P-II | 0.00 | 0.05 | 0.05 |  | 0.00 | 0.05 | 0.04 |  | 0.00 | 0.05 | 0.04 |  | 0.00 | 0.06 | 0.04 |  | 0.00 | 0.07 | 0.05 |
| Pre-test score |  |  |  |  | -0.40 | 0.05 | 1.00 |  | -0.40 | 0.05 | 1.00 |  | -0.40 | 0.05 | 1.00 |  |  |  |  |
| Group |  |  |  |  |  |  |  |  | 4.80 | 1.25 | 0.98 |  | 4.80 | 1.24 | 0.98 |  | 4.79 | 1.32 | 0.98 |
| P-I x Group |  |  |  |  |  |  |  |  |  |  |  |  | 0.23 | 0.10 | 0.66 |  | 0.21 | 0.11 | 0.50 |
| P-II x Group |  |  |  |  |  |  |  |  |  |  |  |  | 0.00 | 0.10 | 0.05 |  | 0.00 | 0.11 | 0.06 |
| **Relative change score** |  |  |  |  |  |  |  |  |  |  |  |  |  |  |  |  |  |  |  |
| Intercept | 7.74 | 1.43 | 1.00 |  | 7.74 | 1.43 | 1.00 |  | 3.09 | 1.81 | 0.53 |  | 3.10 | 1.80 | 0.54 |  | 3.11 | 1.88 | 0.45 |
| P-I | 0.21 | 0.12 | 0.39 |  | 0.24 | 0.11 | 0.57 |  | 0.24 | 0.11 | 0.60 |  | -0.00 | 0.14 | 0.04 |  | 0.00 | 0.17 | 0.04 |
| P-II | 0.00 | 0.12 | 0.06 |  | 0.01 | 0.11 | 0.05 |  | 0.00 | 0.11 | 0.05 |  | 0.00 | 0.14 | 0.03 |  | -0.00 | 0.16 | 0.05 |
| Pre-test score |  |  |  |  | -1.06 | 0.15 | 1.00 |  | -1.06 | 0.14 | 1.00 |  | -1.07 | 0.14 | 1.00 |  |  |  |  |
| Group |  |  |  |  |  |  |  |  | 9.30 | 2.70 | 0.97 |  | 9.30 | 2.70 | 0.97 |  | 9.27 | 2.94 | 0.94 |
| P-I x Group |  |  |  |  |  |  |  |  |  |  |  |  | 0.49 | 0.22 | 0.61 |  | 0.42 | 0.26 | 0.41 |
| P-II x Group |  |  |  |  |  |  |  |  |  |  |  |  | 0.01 | 0.21 | 0.05 |  | 0.01 | 0.24 | 0.06 |
| **Residual score** |  |  |  |  |  |  |  |  |  |  |  |  |  |  |  |  |  |  |  |
| Intercept | 0.00 | 0.00 | 0.00 |  | 0.00 | 0.00 | 0.00 |  | -2.40 | 0.62 | 0.95 |  | -2.40 | 0.62 | 0.95 |  | -2.39 | 0.62 | 0.95 |
| P-I | 0.11 | 0.05 | 0.61 |  | 0.12 | 0.05 | 0.61 |  | 0.12 | 0.05 | 0.65 |  | -0.00 | 0.06 | 0.03 |  | -0.00 | 0.06 | 0.03 |
| P-II | 0.00 | 0.05 | 0.04 |  | 0.00 | 0.05 | 0.04 |  | 0.00 | 0.05 | 0.04 |  | 0.00 | 0.06 | 0.04 |  | 0.00 | 0.06 | 0.03 |
| Pre-test score |  |  |  |  | -0.00 | 0.01 | 0.00 |  | -0.00 | 0.01 | 0.00 |  | -0.01 | 0.02 | 0.00 |  |  |  |  |
| Group |  |  |  |  |  |  |  |  | 4.80 | 1.25 | 0.98 |  | 4.80 | 1.24 | 0.98 |  | 4.79 | 1.24 | 0.98 |
| P-I x Group |  |  |  |  |  |  |  |  |  |  |  |  | 0.23 | 0.10 | 0.66 |  | 0.23 | 0.10 | 0.66 |
| P-II x Group |  |  |  |  |  |  |  |  |  |  |  |  | 0.00 | 0.10 | 0.05 |  | 0.00 | 0.10 | 0.05 |

Table 8: Results of all investigated simulations ordered according to their used model and dependent variable, for a **reliability of .60**, and a sample size of ***n* = 500**

| **Coefficient** | **Model 1** | | |  | **Model 2** | | |  | **Model 3** | | |  | **Model 4** | | |  | **Model 5** | | |
| --- | --- | --- | --- | --- | --- | --- | --- | --- | --- | --- | --- | --- | --- | --- | --- | --- | --- | --- | --- |
|  | ***M*** | ***SE*** | ***P*** |  | ***M*** | ***SE*** | ***P*** |  | ***M*** | ***SE*** | ***P*** |  | ***M*** | ***SE*** | ***P*** |  | ***M*** | ***SE*** | ***P*** |
| **Post-test score** |  |  |  |  |  |  |  |  |  |  |  |  |  |  |  |  |  |  |  |
| Intercept | 52.83 | 0.61 | 1.00 |  | 52.83 | 0.61 | 1.00 |  | 50.43 | 0.77 | 1.00 |  | 50.43 | 0.77 | 1.00 |  | 50.42 | 0.81 | 1.00 |
| P-I | 0.14 | 0.05 | 0.75 |  | 0.12 | 0.05 | 0.74 |  | 0.12 | 0.04 | 0.77 |  | 0.00 | 0.06 | 0.03 |  | 0.00 | 0.06 | 0.03 |
| P-II | -0.00 | 0.05 | 0.05 |  | 0.00 | 0.05 | 0.04 |  | 0.00 | 0.04 | 0.05 |  | 0.00 | 0.06 | 0.04 |  | -0.00 | 0.06 | 0.04 |
| Pre-test score |  |  |  |  | 0.60 | 0.04 | 1.00 |  | 0.60 | 0.04 | 1.00 |  | 0.59 | 0.04 | 1.00 |  |  |  |  |
| Group |  |  |  |  |  |  |  |  | 4.80 | 1.15 | 1.00 |  | 4.80 | 1.15 | 1.00 |  | 4.82 | 1.27 | 0.99 |
| P-I x Group |  |  |  |  |  |  |  |  |  |  |  |  | 0.24 | 0.08 | 0.78 |  | 0.27 | 0.10 | 0.77 |
| P-II x Group |  |  |  |  |  |  |  |  |  |  |  |  | 0.00 | 0.09 | 0.06 |  | 0.00 | 0.10 | 0.04 |
| **Absolute change score** |  |  |  |  |  |  |  |  |  |  |  |  |  |  |  |  |  |  |  |
| Intercept | 2.83 | 0.57 | 1.00 |  | 2.83 | 0.57 | 1.00 |  | 0.43 | 0.74 | 0.14 |  | 0.43 | 0.74 | 0.15 |  | 0.44 | 0.76 | 0.14 |
| P-I | 0.11 | 0.05 | 0.57 |  | 0.12 | 0.05 | 0.74 |  | 0.12 | 0.04 | 0.77 |  | 0.00 | 0.06 | 0.03 |  | 0.00 | 0.06 | 0.04 |
| P-II | 0.00 | 0.05 | 0.06 |  | 0.00 | 0.05 | 0.04 |  | 0.00 | 0.04 | 0.05 |  | 0.00 | 0.06 | 0.04 |  | 0.00 | 0.06 | 0.04 |
| Pre-test score |  |  |  |  | -0.40 | 0.04 | 1.00 |  | -0.40 | 0.04 | 1.00 |  | -0.41 | 0.04 | 1.00 |  |  |  |  |
| Group |  |  |  |  |  |  |  |  | 4.80 | 1.15 | 1.00 |  | 4.80 | 1.15 | 1.00 |  | 4.79 | 1.21 | 0.99 |
| P-I x Group |  |  |  |  |  |  |  |  |  |  |  |  | 0.24 | 0.08 | 0.78 |  | 0.21 | 0.09 | 0.61 |
| P-II x Group |  |  |  |  |  |  |  |  |  |  |  |  | 0.00 | 0.09 | 0.06 |  | 0.00 | 0.10 | 0.06 |
| **Relative change score** |  |  |  |  |  |  |  |  |  |  |  |  |  |  |  |  |  |  |  |
| Intercept | 7.77 | 1.26 | 1.00 |  | 7.77 | 1.26 | 1.00 |  | 3.10 | 1.63 | 0.61 |  | 3.10 | 1.63 | 0.61 |  | 3.11 | 1.68 | 0.53 |
| P-I | 0.21 | 0.11 | 0.47 |  | 0.25 | 0.10 | 0.70 |  | 0.25 | 0.10 | 0.72 |  | 0.00 | 0.12 | 0.03 |  | 0.00 | 0.14 | 0.04 |
| P-II | 0.00 | 0.11 | 0.05 |  | 0.00 | 0.10 | 0.05 |  | 0.00 | 0.10 | 0.05 |  | 0.00 | 0.12 | 0.03 |  | 0.00 | 0.14 | 0.04 |
| Pre-test score |  |  |  |  | -1.06 | 0.13 | 1.00 |  | -1.06 | 0.13 | 1.00 |  | -1.07 | 0.13 | 1.00 |  |  |  |  |
| Group |  |  |  |  |  |  |  |  | 9.35 | 2.50 | 0.99 |  | 9.35 | 2.50 | 0.99 |  | 9.32 | 2.66 | 0.98 |
| P-I x Group |  |  |  |  |  |  |  |  |  |  |  |  | 0.49 | 0.18 | 0.73 |  | 0.42 | 0.21 | 0.50 |
| P-II x Group |  |  |  |  |  |  |  |  |  |  |  |  | 0.00 | 0.20 | 0.05 |  | 0.01 | 0.22 | 0.06 |
| **Residual score** |  |  |  |  |  |  |  |  |  |  |  |  |  |  |  |  |  |  |  |
| Intercept | -0.00 | 0.00 | 0.00 |  | -0.00 | 0.00 | 0.00 |  | -2.40 | 0.58 | 0.98 |  | -2.40 | 0.58 | 0.99 |  | -2.40 | 0.58 | 0.99 |
| P-I | 0.12 | 0.05 | 0.74 |  | 0.12 | 0.05 | 0.74 |  | 0.12 | 0.04 | 0.77 |  | 0.00 | 0.06 | 0.03 |  | 0.00 | 0.06 | 0.03 |
| P-II | 0.00 | 0.05 | 0.04 |  | 0.00 | 0.05 | 0.04 |  | 0.00 | 0.04 | 0.05 |  | 0.00 | 0.06 | 0.04 |  | 0.00 | 0.06 | 0.04 |
| Pre-test score |  |  |  |  | -0.00 | 0.01 | 0.00 |  | -0.00 | 0.01 | 0.00 |  | -0.01 | 0.01 | 0.00 |  |  |  |  |
| Group |  |  |  |  |  |  |  |  | 4.80 | 1.15 | 1.00 |  | 4.80 | 1.15 | 1.00 |  | 4.79 | 1.15 | 1.00 |
| P-I x Group |  |  |  |  |  |  |  |  |  |  |  |  | 0.24 | 0.08 | 0.78 |  | 0.24 | 0.08 | 0.78 |
| P-II x Group |  |  |  |  |  |  |  |  |  |  |  |  | 0.00 | 0.09 | 0.06 |  | 0.00 | 0.09 | 0.06 |

Table 9: Results of all investigated simulations ordered according to their used model and dependent variable, for a **reliability of .70**, and a sample size of ***n* = 50**

| **Coefficient** | **Model 1** | | |  | **Model 2** | | |  | **Model 3** | | |  | **Model 4** | | |  | **Model 5** | | |
| --- | --- | --- | --- | --- | --- | --- | --- | --- | --- | --- | --- | --- | --- | --- | --- | --- | --- | --- | --- |
|  | ***M*** | ***SE*** | ***P*** |  | ***M*** | ***SE*** | ***P*** |  | ***M*** | ***SE*** | ***P*** |  | ***M*** | ***SE*** | ***P*** |  | ***M*** | ***SE*** | ***P*** |
| **Post-test score** |  |  |  |  |  |  |  |  |  |  |  |  |  |  |  |  |  |  |  |
| Intercept | 53.02 | 2.06 | 1.00 |  | 53.02 | 2.06 | 1.00 |  | 50.29 | 2.51 | 1.00 |  | 50.30 | 2.51 | 1.00 |  | 50.29 | 2.71 | 1.00 |
| P-I | 0.15 | 0.18 | 0.12 |  | 0.13 | 0.14 | 0.13 |  | 0.13 | 0.14 | 0.14 |  | -0.00 | 0.18 | 0.02 |  | -0.00 | 0.23 | 0.02 |
| P-II | -0.00 | 0.18 | 0.05 |  | -0.00 | 0.14 | 0.05 |  | -0.00 | 0.14 | 0.05 |  | -0.01 | 0.18 | 0.02 |  | -0.01 | 0.22 | 0.02 |
| Pre-test score |  |  |  |  | 0.70 | 0.14 | 1.00 |  | 0.70 | 0.14 | 1.00 |  | 0.69 | 0.14 | 1.00 |  |  |  |  |
| Group |  |  |  |  |  |  |  |  | 5.46 | 3.65 | 0.53 |  | 5.45 | 3.64 | 0.53 |  | 5.46 | 4.20 | 0.41 |
| P-I x Group |  |  |  |  |  |  |  |  |  |  |  |  | 0.26 | 0.28 | 0.14 |  | 0.30 | 0.36 | 0.13 |
| P-II x Group |  |  |  |  |  |  |  |  |  |  |  |  | 0.01 | 0.28 | 0.04 |  | 0.02 | 0.35 | 0.05 |
| **Absolute change score** |  |  |  |  |  |  |  |  |  |  |  |  |  |  |  |  |  |  |  |
| Intercept | 3.15 | 1.88 | 0.56 |  | 3.15 | 1.88 | 0.59 |  | 0.42 | 2.39 | 0.12 |  | 0.43 | 2.40 | 0.13 |  | 0.44 | 2.41 | 0.10 |
| P-I | 0.12 | 0.15 | 0.11 |  | 0.13 | 0.14 | 0.13 |  | 0.13 | 0.14 | 0.14 |  | -0.00 | 0.18 | 0.02 |  | -0.00 | 0.20 | 0.02 |
| P-II | 0.00 | 0.15 | 0.05 |  | -0.00 | 0.14 | 0.05 |  | -0.00 | 0.14 | 0.05 |  | -0.01 | 0.18 | 0.02 |  | -0.01 | 0.20 | 0.04 |
| Pre-test score |  |  |  |  | -0.30 | 0.14 | 0.56 |  | -0.30 | 0.14 | 0.59 |  | -0.31 | 0.14 | 0.59 |  |  |  |  |
| Group |  |  |  |  |  |  |  |  | 5.46 | 3.65 | 0.53 |  | 5.45 | 3.64 | 0.53 |  | 5.45 | 3.67 | 0.48 |
| P-I x Group |  |  |  |  |  |  |  |  |  |  |  |  | 0.26 | 0.28 | 0.14 |  | 0.25 | 0.29 | 0.12 |
| P-II x Group |  |  |  |  |  |  |  |  |  |  |  |  | 0.01 | 0.28 | 0.04 |  | 0.01 | 0.29 | 0.05 |
| **Relative change score** |  |  |  |  |  |  |  |  |  |  |  |  |  |  |  |  |  |  |  |
| Intercept | 8.06 | 4.20 | 0.65 |  | 8.06 | 4.20 | 0.69 |  | 2.75 | 5.28 | 0.17 |  | 2.76 | 5.31 | 0.17 |  | 2.76 | 5.35 | 0.14 |
| P-I | 0.25 | 0.33 | 0.10 |  | 0.27 | 0.31 | 0.12 |  | 0.27 | 0.30 | 0.13 |  | 0.00 | 0.39 | 0.02 |  | 0.00 | 0.44 | 0.03 |
| P-II | 0.01 | 0.34 | 0.05 |  | 0.00 | 0.31 | 0.05 |  | -0.00 | 0.31 | 0.05 |  | -0.02 | 0.39 | 0.03 |  | -0.01 | 0.45 | 0.04 |
| Pre-test score |  |  |  |  | -0.85 | 0.42 | 0.72 |  | -0.85 | 0.41 | 0.73 |  | -0.86 | 0.41 | 0.74 |  |  |  |  |
| Group |  |  |  |  |  |  |  |  | 10.62 | 7.80 | 0.46 |  | 10.60 | 7.80 | 0.46 |  | 10.62 | 8.02 | 0.41 |
| P-I x Group |  |  |  |  |  |  |  |  |  |  |  |  | 0.54 | 0.61 | 0.12 |  | 0.50 | 0.68 | 0.11 |
| P-II x Group |  |  |  |  |  |  |  |  |  |  |  |  | 0.03 | 0.61 | 0.04 |  | 0.02 | 0.67 | 0.04 |
| **Residual score** |  |  |  |  |  |  |  |  |  |  |  |  |  |  |  |  |  |  |  |
| Intercept | -0.00 | 0.00 | 0.00 |  | -0.00 | 0.00 | 0.00 |  | -2.73 | 1.82 | 0.31 |  | -2.72 | 1.83 | 0.30 |  | -2.67 | 1.80 | 0.30 |
| P-I | 0.13 | 0.14 | 0.12 |  | 0.13 | 0.14 | 0.13 |  | 0.13 | 0.14 | 0.14 |  | -0.00 | 0.18 | 0.02 |  | -0.00 | 0.17 | 0.02 |
| P-II | -0.00 | 0.14 | 0.05 |  | -0.00 | 0.14 | 0.05 |  | -0.00 | 0.14 | 0.05 |  | -0.01 | 0.18 | 0.02 |  | -0.01 | 0.17 | 0.02 |
| Pre-test score |  |  |  |  | -0.00 | 0.04 | 0.00 |  | -0.01 | 0.06 | 0.00 |  | -0.01 | 0.07 | 0.00 |  |  |  |  |
| Group |  |  |  |  |  |  |  |  | 5.46 | 3.65 | 0.53 |  | 5.45 | 3.64 | 0.53 |  | 5.35 | 3.57 | 0.52 |
| P-I x Group |  |  |  |  |  |  |  |  |  |  |  |  | 0.26 | 0.28 | 0.14 |  | 0.26 | 0.27 | 0.14 |
| P-II x Group |  |  |  |  |  |  |  |  |  |  |  |  | 0.01 | 0.28 | 0.04 |  | 0.01 | 0.27 | 0.04 |

Table 10: Results of all investigated simulations ordered according to their used model and dependent variable, for a **reliability of .70**, and a sample size of ***n* = 100**

| **Coefficient** | **Model 1** | | |  | **Model 2** | | |  | **Model 3** | | |  | **Model 4** | | |  | **Model 5** | | |
| --- | --- | --- | --- | --- | --- | --- | --- | --- | --- | --- | --- | --- | --- | --- | --- | --- | --- | --- | --- |
|  | ***M*** | ***SE*** | ***P*** |  | ***M*** | ***SE*** | ***P*** |  | ***M*** | ***SE*** | ***P*** |  | ***M*** | ***SE*** | ***P*** |  | ***M*** | ***SE*** | ***P*** |
| **Post-test score** |  |  |  |  |  |  |  |  |  |  |  |  |  |  |  |  |  |  |  |
| Intercept | 53.11 | 1.40 | 1.00 |  | 53.11 | 1.40 | 1.00 |  | 50.49 | 1.70 | 1.00 |  | 50.49 | 1.69 | 1.00 |  | 50.50 | 1.85 | 1.00 |
| P-I | 0.16 | 0.12 | 0.26 |  | 0.14 | 0.09 | 0.25 |  | 0.14 | 0.09 | 0.28 |  | 0.00 | 0.11 | 0.02 |  | 0.00 | 0.15 | 0.03 |
| P-II | 0.00 | 0.12 | 0.05 |  | 0.00 | 0.10 | 0.06 |  | -0.00 | 0.09 | 0.05 |  | 0.00 | 0.12 | 0.03 |  | 0.00 | 0.15 | 0.02 |
| Pre-test score |  |  |  |  | 0.70 | 0.10 | 1.00 |  | 0.70 | 0.09 | 1.00 |  | 0.70 | 0.09 | 1.00 |  |  |  |  |
| Group |  |  |  |  |  |  |  |  | 5.23 | 2.44 | 0.74 |  | 5.23 | 2.44 | 0.75 |  | 5.22 | 2.80 | 0.60 |
| P-I x Group |  |  |  |  |  |  |  |  |  |  |  |  | 0.27 | 0.19 | 0.29 |  | 0.32 | 0.25 | 0.27 |
| P-II x Group |  |  |  |  |  |  |  |  |  |  |  |  | -0.01 | 0.19 | 0.05 |  | -0.00 | 0.24 | 0.06 |
| **Absolute change score** |  |  |  |  |  |  |  |  |  |  |  |  |  |  |  |  |  |  |  |
| Intercept | 3.12 | 1.29 | 0.80 |  | 3.12 | 1.29 | 0.82 |  | 0.50 | 1.58 | 0.12 |  | 0.50 | 1.57 | 0.12 |  | 0.50 | 1.61 | 0.12 |
| P-I | 0.13 | 0.10 | 0.21 |  | 0.14 | 0.09 | 0.25 |  | 0.14 | 0.09 | 0.28 |  | 0.00 | 0.11 | 0.02 |  | 0.01 | 0.13 | 0.03 |
| P-II | 0.00 | 0.11 | 0.06 |  | 0.00 | 0.10 | 0.06 |  | -0.00 | 0.09 | 0.05 |  | 0.00 | 0.12 | 0.03 |  | 0.00 | 0.13 | 0.03 |
| Pre-test score |  |  |  |  | -0.30 | 0.10 | 0.84 |  | -0.30 | 0.09 | 0.87 |  | -0.30 | 0.09 | 0.88 |  |  |  |  |
| Group |  |  |  |  |  |  |  |  | 5.23 | 2.44 | 0.74 |  | 5.23 | 2.44 | 0.75 |  | 5.24 | 2.51 | 0.72 |
| P-I x Group |  |  |  |  |  |  |  |  |  |  |  |  | 0.27 | 0.19 | 0.29 |  | 0.25 | 0.19 | 0.23 |
| P-II x Group |  |  |  |  |  |  |  |  |  |  |  |  | -0.01 | 0.19 | 0.05 |  | -0.00 | 0.20 | 0.05 |
| **Relative change score** |  |  |  |  |  |  |  |  |  |  |  |  |  |  |  |  |  |  |  |
| Intercept | 7.91 | 2.79 | 0.90 |  | 7.91 | 2.79 | 0.92 |  | 2.84 | 3.43 | 0.22 |  | 2.85 | 3.42 | 0.22 |  | 2.84 | 3.51 | 0.17 |
| P-I | 0.26 | 0.21 | 0.18 |  | 0.28 | 0.20 | 0.25 |  | 0.29 | 0.19 | 0.27 |  | 0.00 | 0.25 | 0.02 |  | 0.01 | 0.28 | 0.03 |
| P-II | 0.00 | 0.24 | 0.06 |  | 0.00 | 0.21 | 0.05 |  | -0.00 | 0.20 | 0.05 |  | 0.00 | 0.26 | 0.02 |  | 0.00 | 0.30 | 0.03 |
| Pre-test score |  |  |  |  | -0.83 | 0.26 | 0.95 |  | -0.83 | 0.25 | 0.95 |  | -0.85 | 0.25 | 0.95 |  |  |  |  |
| Group |  |  |  |  |  |  |  |  | 10.15 | 5.27 | 0.66 |  | 10.14 | 5.27 | 0.67 |  | 10.16 | 5.56 | 0.63 |
| P-I x Group |  |  |  |  |  |  |  |  |  |  |  |  | 0.57 | 0.41 | 0.28 |  | 0.51 | 0.44 | 0.20 |
| P-II x Group |  |  |  |  |  |  |  |  |  |  |  |  | -0.01 | 0.42 | 0.06 |  | -0.01 | 0.46 | 0.05 |
| **Residual score** |  |  |  |  |  |  |  |  |  |  |  |  |  |  |  |  |  |  |  |
| Intercept | 0.00 | 0.00 | 0.00 |  | 0.00 | 0.00 | 0.00 |  | -2.61 | 1.22 | 0.51 |  | -2.61 | 1.21 | 0.51 |  | -2.58 | 1.21 | 0.51 |
| P-I | 0.13 | 0.09 | 0.25 |  | 0.14 | 0.09 | 0.25 |  | 0.14 | 0.09 | 0.28 |  | 0.00 | 0.11 | 0.02 |  | 0.00 | 0.11 | 0.02 |
| P-II | 0.00 | 0.10 | 0.06 |  | 0.00 | 0.10 | 0.06 |  | -0.00 | 0.09 | 0.05 |  | 0.00 | 0.12 | 0.03 |  | 0.00 | 0.12 | 0.03 |
| Pre-test score |  |  |  |  | -0.00 | 0.02 | 0.00 |  | -0.00 | 0.04 | 0.00 |  | -0.01 | 0.04 | 0.00 |  |  |  |  |
| Group |  |  |  |  |  |  |  |  | 5.23 | 2.44 | 0.74 |  | 5.23 | 2.44 | 0.75 |  | 5.18 | 2.42 | 0.75 |
| P-I x Group |  |  |  |  |  |  |  |  |  |  |  |  | 0.27 | 0.19 | 0.29 |  | 0.27 | 0.19 | 0.30 |
| P-II x Group |  |  |  |  |  |  |  |  |  |  |  |  | -0.01 | 0.19 | 0.05 |  | -0.01 | 0.19 | 0.05 |

Table 11: Results of all investigated simulations ordered according to their used model and dependent variable, for a **reliability of .70**, and a sample size of ***n* = 150**

| **Coefficient** | **Model 1** | | |  | **Model 2** | | |  | **Model 3** | | |  | **Model 4** | | |  | **Model 5** | | |
| --- | --- | --- | --- | --- | --- | --- | --- | --- | --- | --- | --- | --- | --- | --- | --- | --- | --- | --- | --- |
|  | ***M*** | ***SE*** | ***P*** |  | ***M*** | ***SE*** | ***P*** |  | ***M*** | ***SE*** | ***P*** |  | ***M*** | ***SE*** | ***P*** |  | ***M*** | ***SE*** | ***P*** |
| **Post-test score** |  |  |  |  |  |  |  |  |  |  |  |  |  |  |  |  |  |  |  |
| Intercept | 53.06 | 1.17 | 1.00 |  | 53.06 | 1.17 | 1.00 |  | 50.49 | 1.39 | 1.00 |  | 50.49 | 1.39 | 1.00 |  | 50.53 | 1.50 | 1.00 |
| P-I | 0.16 | 0.10 | 0.35 |  | 0.13 | 0.08 | 0.36 |  | 0.13 | 0.07 | 0.39 |  | -0.00 | 0.10 | 0.04 |  | -0.01 | 0.12 | 0.02 |
| P-II | -0.00 | 0.10 | 0.06 |  | -0.00 | 0.08 | 0.06 |  | -0.00 | 0.08 | 0.06 |  | 0.00 | 0.09 | 0.02 |  | 0.01 | 0.12 | 0.03 |
| Pre-test score |  |  |  |  | 0.70 | 0.09 | 1.00 |  | 0.70 | 0.08 | 1.00 |  | 0.69 | 0.08 | 1.00 |  |  |  |  |
| Group |  |  |  |  |  |  |  |  | 5.16 | 2.11 | 0.85 |  | 5.16 | 2.11 | 0.86 |  | 5.08 | 2.41 | 0.73 |
| P-I x Group |  |  |  |  |  |  |  |  |  |  |  |  | 0.27 | 0.16 | 0.43 |  | 0.33 | 0.20 | 0.41 |
| P-II x Group |  |  |  |  |  |  |  |  |  |  |  |  | -0.01 | 0.15 | 0.04 |  | -0.01 | 0.19 | 0.05 |
| **Absolute change score** |  |  |  |  |  |  |  |  |  |  |  |  |  |  |  |  |  |  |  |
| Intercept | 3.04 | 1.07 | 0.90 |  | 3.04 | 1.07 | 0.91 |  | 0.46 | 1.31 | 0.13 |  | 0.46 | 1.30 | 0.13 |  | 0.44 | 1.33 | 0.11 |
| P-I | 0.12 | 0.08 | 0.29 |  | 0.13 | 0.08 | 0.36 |  | 0.13 | 0.07 | 0.39 |  | -0.00 | 0.10 | 0.04 |  | -0.00 | 0.11 | 0.05 |
| P-II | -0.00 | 0.08 | 0.06 |  | -0.00 | 0.08 | 0.06 |  | -0.00 | 0.08 | 0.06 |  | 0.00 | 0.09 | 0.02 |  | -0.00 | 0.10 | 0.03 |
| Pre-test score |  |  |  |  | -0.30 | 0.09 | 0.93 |  | -0.30 | 0.08 | 0.95 |  | -0.31 | 0.08 | 0.96 |  |  |  |  |
| Group |  |  |  |  |  |  |  |  | 5.16 | 2.11 | 0.85 |  | 5.16 | 2.11 | 0.86 |  | 5.19 | 2.17 | 0.83 |
| P-I x Group |  |  |  |  |  |  |  |  |  |  |  |  | 0.27 | 0.16 | 0.43 |  | 0.25 | 0.16 | 0.34 |
| P-II x Group |  |  |  |  |  |  |  |  |  |  |  |  | -0.01 | 0.15 | 0.04 |  | -0.01 | 0.16 | 0.04 |
| **Relative change score** |  |  |  |  |  |  |  |  |  |  |  |  |  |  |  |  |  |  |  |
| Intercept | 7.73 | 2.36 | 0.97 |  | 7.73 | 2.36 | 0.97 |  | 2.73 | 2.81 | 0.27 |  | 2.74 | 2.80 | 0.28 |  | 2.70 | 2.87 | 0.22 |
| P-I | 0.25 | 0.17 | 0.26 |  | 0.27 | 0.16 | 0.35 |  | 0.28 | 0.16 | 0.37 |  | -0.01 | 0.21 | 0.04 |  | -0.00 | 0.24 | 0.05 |
| P-II | -0.01 | 0.21 | 0.05 |  | -0.01 | 0.19 | 0.06 |  | -0.01 | 0.18 | 0.06 |  | 0.00 | 0.20 | 0.03 |  | -0.00 | 0.22 | 0.03 |
| Pre-test score |  |  |  |  | -0.84 | 0.30 | 0.98 |  | -0.84 | 0.30 | 0.99 |  | -0.85 | 0.29 | 0.99 |  |  |  |  |
| Group |  |  |  |  |  |  |  |  | 10.00 | 4.69 | 0.79 |  | 10.00 | 4.69 | 0.79 |  | 10.07 | 4.81 | 0.76 |
| P-I x Group |  |  |  |  |  |  |  |  |  |  |  |  | 0.57 | 0.34 | 0.41 |  | 0.50 | 0.36 | 0.27 |
| P-II x Group |  |  |  |  |  |  |  |  |  |  |  |  | -0.02 | 0.37 | 0.04 |  | -0.01 | 0.40 | 0.04 |
| **Residual score** |  |  |  |  |  |  |  |  |  |  |  |  |  |  |  |  |  |  |  |
| Intercept | 0.00 | 0.00 | 0.00 |  | 0.00 | 0.00 | 0.00 |  | -2.58 | 1.05 | 0.68 |  | -2.58 | 1.05 | 0.69 |  | -2.56 | 1.05 | 0.69 |
| P-I | 0.13 | 0.07 | 0.36 |  | 0.13 | 0.08 | 0.36 |  | 0.13 | 0.07 | 0.39 |  | -0.00 | 0.10 | 0.04 |  | -0.00 | 0.10 | 0.04 |
| P-II | -0.00 | 0.08 | 0.06 |  | -0.00 | 0.08 | 0.06 |  | -0.00 | 0.08 | 0.06 |  | 0.00 | 0.09 | 0.02 |  | 0.00 | 0.09 | 0.02 |
| Pre-test score |  |  |  |  | -0.00 | 0.02 | 0.00 |  | -0.00 | 0.03 | 0.00 |  | -0.01 | 0.03 | 0.00 |  |  |  |  |
| Group |  |  |  |  |  |  |  |  | 5.16 | 2.11 | 0.85 |  | 5.16 | 2.11 | 0.86 |  | 5.12 | 2.10 | 0.86 |
| P-I x Group |  |  |  |  |  |  |  |  |  |  |  |  | 0.27 | 0.16 | 0.43 |  | 0.27 | 0.15 | 0.43 |
| P-II x Group |  |  |  |  |  |  |  |  |  |  |  |  | -0.01 | 0.15 | 0.04 |  | -0.01 | 0.15 | 0.04 |

Table 12: Results of all investigated simulations ordered according to their used model and dependent variable, for a **reliability of .70**, and a sample size of ***n* = 200**

| **Coefficient** | **Model 1** | | |  | **Model 2** | | |  | **Model 3** | | |  | **Model 4** | | |  | **Model 5** | | |
| --- | --- | --- | --- | --- | --- | --- | --- | --- | --- | --- | --- | --- | --- | --- | --- | --- | --- | --- | --- |
|  | ***M*** | ***SE*** | ***P*** |  | ***M*** | ***SE*** | ***P*** |  | ***M*** | ***SE*** | ***P*** |  | ***M*** | ***SE*** | ***P*** |  | ***M*** | ***SE*** | ***P*** |
| **Post-test score** |  |  |  |  |  |  |  |  |  |  |  |  |  |  |  |  |  |  |  |
| Intercept | 53.10 | 1.02 | 1.00 |  | 53.10 | 1.02 | 1.00 |  | 50.47 | 1.21 | 1.00 |  | 50.47 | 1.20 | 1.00 |  | 50.44 | 1.30 | 1.00 |
| P-I | 0.15 | 0.08 | 0.45 |  | 0.13 | 0.07 | 0.46 |  | 0.13 | 0.06 | 0.51 |  | -0.00 | 0.08 | 0.02 |  | -0.00 | 0.10 | 0.02 |
| P-II | -0.00 | 0.08 | 0.05 |  | -0.00 | 0.06 | 0.03 |  | -0.00 | 0.06 | 0.03 |  | -0.00 | 0.08 | 0.02 |  | -0.00 | 0.10 | 0.03 |
| Pre-test score |  |  |  |  | 0.70 | 0.07 | 1.00 |  | 0.70 | 0.07 | 1.00 |  | 0.70 | 0.07 | 1.00 |  |  |  |  |
| Group |  |  |  |  |  |  |  |  | 5.26 | 1.77 | 0.93 |  | 5.26 | 1.77 | 0.93 |  | 5.32 | 2.08 | 0.84 |
| P-I x Group |  |  |  |  |  |  |  |  |  |  |  |  | 0.27 | 0.13 | 0.52 |  | 0.31 | 0.17 | 0.50 |
| P-II x Group |  |  |  |  |  |  |  |  |  |  |  |  | 0.00 | 0.13 | 0.05 |  | 0.00 | 0.16 | 0.06 |
| **Absolute change score** |  |  |  |  |  |  |  |  |  |  |  |  |  |  |  |  |  |  |  |
| Intercept | 3.08 | 0.92 | 0.97 |  | 3.08 | 0.92 | 0.98 |  | 0.45 | 1.11 | 0.12 |  | 0.45 | 1.10 | 0.12 |  | 0.46 | 1.11 | 0.10 |
| P-I | 0.12 | 0.07 | 0.39 |  | 0.13 | 0.07 | 0.46 |  | 0.13 | 0.06 | 0.51 |  | -0.00 | 0.08 | 0.02 |  | -0.00 | 0.09 | 0.04 |
| P-II | -0.00 | 0.07 | 0.03 |  | -0.00 | 0.06 | 0.03 |  | -0.00 | 0.06 | 0.03 |  | -0.00 | 0.08 | 0.02 |  | -0.00 | 0.09 | 0.03 |
| Pre-test score |  |  |  |  | -0.30 | 0.07 | 0.98 |  | -0.30 | 0.07 | 0.99 |  | -0.30 | 0.07 | 0.99 |  |  |  |  |
| Group |  |  |  |  |  |  |  |  | 5.26 | 1.77 | 0.93 |  | 5.26 | 1.77 | 0.93 |  | 5.24 | 1.79 | 0.92 |
| P-I x Group |  |  |  |  |  |  |  |  |  |  |  |  | 0.27 | 0.13 | 0.52 |  | 0.25 | 0.13 | 0.42 |
| P-II x Group |  |  |  |  |  |  |  |  |  |  |  |  | 0.00 | 0.13 | 0.05 |  | 0.00 | 0.13 | 0.04 |
| **Relative change score** |  |  |  |  |  |  |  |  |  |  |  |  |  |  |  |  |  |  |  |
| Intercept | 7.80 | 2.00 | 0.99 |  | 7.80 | 2.00 | 1.00 |  | 2.70 | 2.41 | 0.32 |  | 2.70 | 2.40 | 0.32 |  | 2.73 | 2.41 | 0.27 |
| P-I | 0.25 | 0.15 | 0.34 |  | 0.27 | 0.14 | 0.43 |  | 0.27 | 0.14 | 0.47 |  | -0.00 | 0.18 | 0.02 |  | -0.00 | 0.20 | 0.03 |
| P-II | 0.00 | 0.15 | 0.03 |  | -0.00 | 0.14 | 0.03 |  | -0.00 | 0.13 | 0.03 |  | -0.00 | 0.17 | 0.03 |  | -0.00 | 0.19 | 0.03 |
| Pre-test score |  |  |  |  | -0.82 | 0.19 | 1.00 |  | -0.83 | 0.18 | 1.00 |  | -0.84 | 0.18 | 1.00 |  |  |  |  |
| Group |  |  |  |  |  |  |  |  | 10.20 | 3.77 | 0.89 |  | 10.20 | 3.77 | 0.89 |  | 10.14 | 3.87 | 0.86 |
| P-I x Group |  |  |  |  |  |  |  |  |  |  |  |  | 0.55 | 0.27 | 0.48 |  | 0.50 | 0.29 | 0.35 |
| P-II x Group |  |  |  |  |  |  |  |  |  |  |  |  | 0.01 | 0.28 | 0.04 |  | 0.01 | 0.30 | 0.05 |
| **Residual score** |  |  |  |  |  |  |  |  |  |  |  |  |  |  |  |  |  |  |  |
| Intercept | -0.00 | 0.00 | 0.00 |  | -0.00 | 0.00 | 0.00 |  | -2.63 | 0.88 | 0.82 |  | -2.63 | 0.88 | 0.83 |  | -2.62 | 0.88 | 0.82 |
| P-I | 0.13 | 0.07 | 0.46 |  | 0.13 | 0.07 | 0.46 |  | 0.13 | 0.06 | 0.51 |  | -0.00 | 0.08 | 0.02 |  | -0.00 | 0.08 | 0.02 |
| P-II | -0.00 | 0.06 | 0.03 |  | -0.00 | 0.06 | 0.03 |  | -0.00 | 0.06 | 0.03 |  | -0.00 | 0.08 | 0.02 |  | -0.00 | 0.08 | 0.03 |
| Pre-test score |  |  |  |  | -0.00 | 0.01 | 0.00 |  | -0.01 | 0.02 | 0.00 |  | -0.01 | 0.03 | 0.00 |  |  |  |  |
| Group |  |  |  |  |  |  |  |  | 5.26 | 1.77 | 0.93 |  | 5.26 | 1.77 | 0.93 |  | 5.24 | 1.76 | 0.93 |
| P-I x Group |  |  |  |  |  |  |  |  |  |  |  |  | 0.27 | 0.13 | 0.52 |  | 0.26 | 0.12 | 0.52 |
| P-II x Group |  |  |  |  |  |  |  |  |  |  |  |  | 0.00 | 0.13 | 0.05 |  | 0.00 | 0.13 | 0.04 |

Table 13: Results of all investigated simulations ordered according to their used model and dependent variable, for a **reliability of .70**, and a sample size of ***n* = 250**

| **Coefficient** | **Model 1** | | |  | **Model 2** | | |  | **Model 3** | | |  | **Model 4** | | |  | **Model 5** | | |
| --- | --- | --- | --- | --- | --- | --- | --- | --- | --- | --- | --- | --- | --- | --- | --- | --- | --- | --- | --- |
|  | ***M*** | ***SE*** | ***P*** |  | ***M*** | ***SE*** | ***P*** |  | ***M*** | ***SE*** | ***P*** |  | ***M*** | ***SE*** | ***P*** |  | ***M*** | ***SE*** | ***P*** |
| **Post-test score** |  |  |  |  |  |  |  |  |  |  |  |  |  |  |  |  |  |  |  |
| Intercept | 53.05 | 0.94 | 1.00 |  | 53.05 | 0.94 | 1.00 |  | 50.46 | 1.08 | 1.00 |  | 50.46 | 1.08 | 1.00 |  | 50.47 | 1.17 | 1.00 |
| P-I | 0.16 | 0.08 | 0.58 |  | 0.14 | 0.06 | 0.61 |  | 0.14 | 0.06 | 0.64 |  | 0.01 | 0.07 | 0.03 |  | 0.01 | 0.09 | 0.02 |
| P-II | 0.00 | 0.08 | 0.05 |  | 0.00 | 0.06 | 0.06 |  | 0.00 | 0.06 | 0.06 |  | -0.00 | 0.08 | 0.03 |  | -0.00 | 0.09 | 0.02 |
| Pre-test score |  |  |  |  | 0.70 | 0.06 | 1.00 |  | 0.70 | 0.06 | 1.00 |  | 0.69 | 0.06 | 1.00 |  |  |  |  |
| Group |  |  |  |  |  |  |  |  | 5.18 | 1.54 | 0.97 |  | 5.18 | 1.54 | 0.97 |  | 5.15 | 1.77 | 0.89 |
| P-I x Group |  |  |  |  |  |  |  |  |  |  |  |  | 0.26 | 0.12 | 0.62 |  | 0.32 | 0.15 | 0.58 |
| P-II x Group |  |  |  |  |  |  |  |  |  |  |  |  | -0.00 | 0.11 | 0.05 |  | -0.00 | 0.15 | 0.06 |
| **Absolute change score** |  |  |  |  |  |  |  |  |  |  |  |  |  |  |  |  |  |  |  |
| Intercept | 3.02 | 0.85 | 0.98 |  | 3.02 | 0.85 | 0.99 |  | 0.43 | 1.01 | 0.14 |  | 0.44 | 1.01 | 0.15 |  | 0.43 | 1.02 | 0.13 |
| P-I | 0.13 | 0.06 | 0.50 |  | 0.14 | 0.06 | 0.61 |  | 0.14 | 0.06 | 0.64 |  | 0.01 | 0.07 | 0.03 |  | 0.01 | 0.08 | 0.04 |
| P-II | 0.00 | 0.07 | 0.06 |  | 0.00 | 0.06 | 0.06 |  | 0.00 | 0.06 | 0.06 |  | -0.00 | 0.08 | 0.03 |  | 0.00 | 0.08 | 0.04 |
| Pre-test score |  |  |  |  | -0.30 | 0.06 | 1.00 |  | -0.30 | 0.06 | 0.99 |  | -0.31 | 0.06 | 1.00 |  |  |  |  |
| Group |  |  |  |  |  |  |  |  | 5.18 | 1.54 | 0.97 |  | 5.18 | 1.54 | 0.97 |  | 5.19 | 1.57 | 0.97 |
| P-I x Group |  |  |  |  |  |  |  |  |  |  |  |  | 0.26 | 0.12 | 0.62 |  | 0.24 | 0.12 | 0.48 |
| P-II x Group |  |  |  |  |  |  |  |  |  |  |  |  | -0.00 | 0.11 | 0.05 |  | -0.00 | 0.12 | 0.04 |
| **Relative change score** |  |  |  |  |  |  |  |  |  |  |  |  |  |  |  |  |  |  |  |
| Intercept | 7.70 | 1.86 | 1.00 |  | 7.70 | 1.86 | 1.00 |  | 2.70 | 2.20 | 0.35 |  | 2.71 | 2.20 | 0.36 |  | 2.70 | 2.23 | 0.31 |
| P-I | 0.25 | 0.14 | 0.43 |  | 0.28 | 0.13 | 0.58 |  | 0.28 | 0.12 | 0.59 |  | 0.01 | 0.16 | 0.04 |  | 0.01 | 0.18 | 0.05 |
| P-II | 0.00 | 0.15 | 0.06 |  | 0.00 | 0.13 | 0.06 |  | 0.00 | 0.13 | 0.05 |  | -0.00 | 0.16 | 0.03 |  | 0.00 | 0.18 | 0.04 |
| Pre-test score |  |  |  |  | -0.83 | 0.16 | 1.00 |  | -0.83 | 0.15 | 1.00 |  | -0.84 | 0.15 | 1.00 |  |  |  |  |
| Group |  |  |  |  |  |  |  |  | 9.99 | 3.28 | 0.94 |  | 9.99 | 3.28 | 0.94 |  | 10.03 | 3.41 | 0.92 |
| P-I x Group |  |  |  |  |  |  |  |  |  |  |  |  | 0.54 | 0.25 | 0.58 |  | 0.48 | 0.27 | 0.41 |
| P-II x Group |  |  |  |  |  |  |  |  |  |  |  |  | -0.00 | 0.25 | 0.05 |  | -0.00 | 0.27 | 0.04 |
| **Residual score** |  |  |  |  |  |  |  |  |  |  |  |  |  |  |  |  |  |  |  |
| Intercept | 0.00 | 0.00 | 0.00 |  | 0.00 | 0.00 | 0.00 |  | -2.59 | 0.77 | 0.90 |  | -2.59 | 0.77 | 0.91 |  | -2.58 | 0.76 | 0.91 |
| P-I | 0.14 | 0.06 | 0.61 |  | 0.14 | 0.06 | 0.61 |  | 0.14 | 0.06 | 0.64 |  | 0.01 | 0.07 | 0.03 |  | 0.01 | 0.07 | 0.03 |
| P-II | 0.00 | 0.06 | 0.06 |  | 0.00 | 0.06 | 0.06 |  | 0.00 | 0.06 | 0.06 |  | -0.00 | 0.08 | 0.03 |  | 0.00 | 0.08 | 0.03 |
| Pre-test score |  |  |  |  | -0.01 | 0.01 | 0.00 |  | -0.00 | 0.02 | 0.00 |  | -0.01 | 0.02 | 0.00 |  |  |  |  |
| Group |  |  |  |  |  |  |  |  | 5.18 | 1.54 | 0.97 |  | 5.18 | 1.54 | 0.97 |  | 5.16 | 1.53 | 0.97 |
| P-I x Group |  |  |  |  |  |  |  |  |  |  |  |  | 0.26 | 0.12 | 0.62 |  | 0.26 | 0.11 | 0.62 |
| P-II x Group |  |  |  |  |  |  |  |  |  |  |  |  | -0.00 | 0.11 | 0.05 |  | -0.00 | 0.11 | 0.05 |

Table 14: Results of all investigated simulations ordered according to their used model and dependent variable, for a **reliability of .70**, and a sample size of ***n* = 300**

| **Coefficient** | **Model 1** | | |  | **Model 2** | | |  | **Model 3** | | |  | **Model 4** | | |  | **Model 5** | | |
| --- | --- | --- | --- | --- | --- | --- | --- | --- | --- | --- | --- | --- | --- | --- | --- | --- | --- | --- | --- |
|  | ***M*** | ***SE*** | ***P*** |  | ***M*** | ***SE*** | ***P*** |  | ***M*** | ***SE*** | ***P*** |  | ***M*** | ***SE*** | ***P*** |  | ***M*** | ***SE*** | ***P*** |
| **Post-test score** |  |  |  |  |  |  |  |  |  |  |  |  |  |  |  |  |  |  |  |
| Intercept | 53.04 | 0.82 | 1.00 |  | 53.04 | 0.82 | 1.00 |  | 50.49 | 1.02 | 1.00 |  | 50.50 | 1.01 | 1.00 |  | 50.50 | 1.09 | 1.00 |
| P-I | 0.16 | 0.07 | 0.62 |  | 0.13 | 0.05 | 0.66 |  | 0.13 | 0.05 | 0.69 |  | -0.00 | 0.07 | 0.02 |  | -0.00 | 0.08 | 0.03 |
| P-II | -0.00 | 0.07 | 0.05 |  | 0.00 | 0.05 | 0.05 |  | 0.00 | 0.05 | 0.05 |  | -0.00 | 0.07 | 0.02 |  | -0.00 | 0.08 | 0.02 |
| Pre-test score |  |  |  |  | 0.70 | 0.05 | 1.00 |  | 0.70 | 0.05 | 1.00 |  | 0.69 | 0.05 | 1.00 |  |  |  |  |
| Group |  |  |  |  |  |  |  |  | 5.10 | 1.44 | 0.98 |  | 5.10 | 1.44 | 0.98 |  | 5.10 | 1.65 | 0.94 |
| P-I x Group |  |  |  |  |  |  |  |  |  |  |  |  | 0.27 | 0.10 | 0.72 |  | 0.32 | 0.14 | 0.68 |
| P-II x Group |  |  |  |  |  |  |  |  |  |  |  |  | 0.00 | 0.10 | 0.04 |  | 0.00 | 0.13 | 0.05 |
| **Absolute change score** |  |  |  |  |  |  |  |  |  |  |  |  |  |  |  |  |  |  |  |
| Intercept | 3.06 | 0.74 | 1.00 |  | 3.06 | 0.74 | 1.00 |  | 0.51 | 0.94 | 0.19 |  | 0.52 | 0.94 | 0.19 |  | 0.52 | 0.95 | 0.17 |
| P-I | 0.12 | 0.06 | 0.54 |  | 0.13 | 0.05 | 0.66 |  | 0.13 | 0.05 | 0.69 |  | -0.00 | 0.07 | 0.02 |  | -0.00 | 0.07 | 0.03 |
| P-II | 0.00 | 0.06 | 0.04 |  | 0.00 | 0.05 | 0.05 |  | 0.00 | 0.05 | 0.05 |  | -0.00 | 0.07 | 0.02 |  | 0.00 | 0.07 | 0.02 |
| Pre-test score |  |  |  |  | -0.30 | 0.05 | 1.00 |  | -0.30 | 0.05 | 1.00 |  | -0.31 | 0.05 | 1.00 |  |  |  |  |
| Group |  |  |  |  |  |  |  |  | 5.10 | 1.44 | 0.98 |  | 5.10 | 1.44 | 0.98 |  | 5.10 | 1.48 | 0.98 |
| P-I x Group |  |  |  |  |  |  |  |  |  |  |  |  | 0.27 | 0.10 | 0.72 |  | 0.25 | 0.11 | 0.60 |
| P-II x Group |  |  |  |  |  |  |  |  |  |  |  |  | 0.00 | 0.10 | 0.04 |  | 0.00 | 0.11 | 0.04 |
| **Relative change score** |  |  |  |  |  |  |  |  |  |  |  |  |  |  |  |  |  |  |  |
| Intercept | 7.81 | 1.63 | 1.00 |  | 7.81 | 1.63 | 1.00 |  | 2.89 | 2.05 | 0.44 |  | 2.90 | 2.05 | 0.45 |  | 2.90 | 2.09 | 0.40 |
| P-I | 0.24 | 0.13 | 0.46 |  | 0.27 | 0.12 | 0.62 |  | 0.27 | 0.11 | 0.65 |  | -0.00 | 0.14 | 0.02 |  | -0.00 | 0.16 | 0.03 |
| P-II | 0.00 | 0.13 | 0.05 |  | 0.00 | 0.12 | 0.04 |  | 0.00 | 0.11 | 0.05 |  | -0.00 | 0.14 | 0.02 |  | 0.00 | 0.16 | 0.02 |
| Pre-test score |  |  |  |  | -0.84 | 0.16 | 1.00 |  | -0.84 | 0.16 | 1.00 |  | -0.85 | 0.16 | 1.00 |  |  |  |  |
| Group |  |  |  |  |  |  |  |  | 9.86 | 3.12 | 0.96 |  | 9.86 | 3.12 | 0.96 |  | 9.85 | 3.27 | 0.94 |
| P-I x Group |  |  |  |  |  |  |  |  |  |  |  |  | 0.56 | 0.22 | 0.68 |  | 0.49 | 0.24 | 0.50 |
| P-II x Group |  |  |  |  |  |  |  |  |  |  |  |  | 0.01 | 0.23 | 0.04 |  | 0.00 | 0.24 | 0.03 |
| **Residual score** |  |  |  |  |  |  |  |  |  |  |  |  |  |  |  |  |  |  |  |
| Intercept | -0.00 | 0.00 | 0.00 |  | -0.00 | 0.00 | 0.00 |  | -2.55 | 0.72 | 0.93 |  | -2.54 | 0.72 | 0.93 |  | -2.54 | 0.72 | 0.93 |
| P-I | 0.13 | 0.05 | 0.66 |  | 0.13 | 0.05 | 0.66 |  | 0.13 | 0.05 | 0.69 |  | -0.00 | 0.07 | 0.02 |  | -0.00 | 0.06 | 0.02 |
| P-II | 0.00 | 0.05 | 0.05 |  | 0.00 | 0.05 | 0.05 |  | 0.00 | 0.05 | 0.05 |  | -0.00 | 0.07 | 0.02 |  | -0.00 | 0.07 | 0.02 |
| Pre-test score |  |  |  |  | -0.00 | 0.01 | 0.00 |  | -0.00 | 0.02 | 0.00 |  | -0.01 | 0.02 | 0.00 |  |  |  |  |
| Group |  |  |  |  |  |  |  |  | 5.10 | 1.44 | 0.98 |  | 5.10 | 1.44 | 0.98 |  | 5.08 | 1.44 | 0.98 |
| P-I x Group |  |  |  |  |  |  |  |  |  |  |  |  | 0.27 | 0.10 | 0.72 |  | 0.27 | 0.10 | 0.72 |
| P-II x Group |  |  |  |  |  |  |  |  |  |  |  |  | 0.00 | 0.10 | 0.04 |  | 0.00 | 0.10 | 0.04 |

Table 15: Results of all investigated simulations ordered according to their used model and dependent variable, for a **reliability of .70**, and a sample size of ***n* = 400**

| **Coefficient** | **Model 1** | | |  | **Model 2** | | |  | **Model 3** | | |  | **Model 4** | | |  | **Model 5** | | |
| --- | --- | --- | --- | --- | --- | --- | --- | --- | --- | --- | --- | --- | --- | --- | --- | --- | --- | --- | --- |
|  | ***M*** | ***SE*** | ***P*** |  | ***M*** | ***SE*** | ***P*** |  | ***M*** | ***SE*** | ***P*** |  | ***M*** | ***SE*** | ***P*** |  | ***M*** | ***SE*** | ***P*** |
| **Post-test score** |  |  |  |  |  |  |  |  |  |  |  |  |  |  |  |  |  |  |  |
| Intercept | 53.09 | 0.71 | 1.00 |  | 53.09 | 0.71 | 1.00 |  | 50.50 | 0.83 | 1.00 |  | 50.51 | 0.83 | 1.00 |  | 50.51 | 0.90 | 1.00 |
| P-I | 0.16 | 0.06 | 0.78 |  | 0.13 | 0.05 | 0.80 |  | 0.13 | 0.04 | 0.84 |  | -0.00 | 0.06 | 0.02 |  | 0.00 | 0.07 | 0.03 |
| P-II | 0.00 | 0.06 | 0.05 |  | 0.00 | 0.05 | 0.06 |  | 0.00 | 0.05 | 0.06 |  | 0.00 | 0.06 | 0.03 |  | 0.00 | 0.07 | 0.02 |
| Pre-test score |  |  |  |  | 0.70 | 0.05 | 1.00 |  | 0.70 | 0.05 | 1.00 |  | 0.69 | 0.04 | 1.00 |  |  |  |  |
| Group |  |  |  |  |  |  |  |  | 5.17 | 1.19 | 0.99 |  | 5.16 | 1.19 | 0.99 |  | 5.15 | 1.35 | 0.98 |
| P-I x Group |  |  |  |  |  |  |  |  |  |  |  |  | 0.27 | 0.09 | 0.86 |  | 0.33 | 0.12 | 0.80 |
| P-II x Group |  |  |  |  |  |  |  |  |  |  |  |  | 0.00 | 0.09 | 0.04 |  | -0.00 | 0.11 | 0.04 |
| **Absolute change score** |  |  |  |  |  |  |  |  |  |  |  |  |  |  |  |  |  |  |  |
| Intercept | 3.07 | 0.64 | 1.00 |  | 3.07 | 0.64 | 1.00 |  | 0.49 | 0.76 | 0.16 |  | 0.50 | 0.76 | 0.16 |  | 0.49 | 0.78 | 0.15 |
| P-I | 0.12 | 0.05 | 0.68 |  | 0.13 | 0.05 | 0.80 |  | 0.13 | 0.04 | 0.84 |  | -0.00 | 0.06 | 0.02 |  | -0.00 | 0.06 | 0.04 |
| P-II | 0.00 | 0.05 | 0.06 |  | 0.00 | 0.05 | 0.06 |  | 0.00 | 0.05 | 0.06 |  | 0.00 | 0.06 | 0.03 |  | 0.00 | 0.06 | 0.04 |
| Pre-test score |  |  |  |  | -0.30 | 0.05 | 1.00 |  | -0.30 | 0.05 | 1.00 |  | -0.31 | 0.04 | 1.00 |  |  |  |  |
| Group |  |  |  |  |  |  |  |  | 5.17 | 1.19 | 0.99 |  | 5.16 | 1.19 | 0.99 |  | 5.17 | 1.24 | 1.00 |
| P-I x Group |  |  |  |  |  |  |  |  |  |  |  |  | 0.27 | 0.09 | 0.86 |  | 0.25 | 0.09 | 0.75 |
| P-II x Group |  |  |  |  |  |  |  |  |  |  |  |  | 0.00 | 0.09 | 0.04 |  | 0.00 | 0.09 | 0.05 |
| **Relative change score** |  |  |  |  |  |  |  |  |  |  |  |  |  |  |  |  |  |  |  |
| Intercept | 7.79 | 1.41 | 1.00 |  | 7.79 | 1.41 | 1.00 |  | 2.81 | 1.69 | 0.50 |  | 2.83 | 1.69 | 0.51 |  | 2.82 | 1.73 | 0.45 |
| P-I | 0.24 | 0.11 | 0.57 |  | 0.27 | 0.10 | 0.78 |  | 0.28 | 0.10 | 0.81 |  | -0.01 | 0.13 | 0.03 |  | -0.01 | 0.14 | 0.04 |
| P-II | 0.01 | 0.12 | 0.06 |  | 0.01 | 0.11 | 0.06 |  | 0.01 | 0.10 | 0.06 |  | 0.01 | 0.12 | 0.03 |  | 0.01 | 0.14 | 0.04 |
| Pre-test score |  |  |  |  | -0.83 | 0.13 | 1.00 |  | -0.83 | 0.13 | 1.00 |  | -0.84 | 0.13 | 1.00 |  |  |  |  |
| Group |  |  |  |  |  |  |  |  | 9.95 | 2.56 | 0.99 |  | 9.95 | 2.56 | 0.99 |  | 9.97 | 2.73 | 0.98 |
| P-I x Group |  |  |  |  |  |  |  |  |  |  |  |  | 0.57 | 0.20 | 0.83 |  | 0.50 | 0.21 | 0.64 |
| P-II x Group |  |  |  |  |  |  |  |  |  |  |  |  | 0.00 | 0.19 | 0.04 |  | 0.00 | 0.21 | 0.05 |
| **Residual score** |  |  |  |  |  |  |  |  |  |  |  |  |  |  |  |  |  |  |  |
| Intercept | -0.00 | 0.00 | 0.00 |  | -0.00 | 0.00 | 0.00 |  | -2.58 | 0.59 | 0.99 |  | -2.58 | 0.59 | 0.99 |  | -2.57 | 0.59 | 0.99 |
| P-I | 0.13 | 0.05 | 0.80 |  | 0.13 | 0.05 | 0.80 |  | 0.13 | 0.04 | 0.84 |  | -0.00 | 0.06 | 0.02 |  | -0.00 | 0.06 | 0.02 |
| P-II | 0.00 | 0.05 | 0.07 |  | 0.00 | 0.05 | 0.06 |  | 0.00 | 0.05 | 0.06 |  | 0.00 | 0.06 | 0.03 |  | 0.00 | 0.06 | 0.03 |
| Pre-test score |  |  |  |  | -0.01 | 0.01 | 0.00 |  | -0.01 | 0.02 | 0.00 |  | -0.01 | 0.02 | 0.00 |  |  |  |  |
| Group |  |  |  |  |  |  |  |  | 5.17 | 1.19 | 0.99 |  | 5.16 | 1.19 | 0.99 |  | 5.15 | 1.19 | 0.99 |
| P-I x Group |  |  |  |  |  |  |  |  |  |  |  |  | 0.27 | 0.09 | 0.86 |  | 0.27 | 0.09 | 0.86 |
| P-II x Group |  |  |  |  |  |  |  |  |  |  |  |  | 0.00 | 0.09 | 0.04 |  | 0.00 | 0.09 | 0.04 |

Table 16: Results of all investigated simulations ordered according to their used model and dependent variable, for a **reliability of .70**, and a sample size of ***n* = 500**

| **Coefficient** | **Model 1** | | |  | **Model 2** | | |  | **Model 3** | | |  | **Model 4** | | |  | **Model 5** | | |
| --- | --- | --- | --- | --- | --- | --- | --- | --- | --- | --- | --- | --- | --- | --- | --- | --- | --- | --- | --- |
|  | ***M*** | ***SE*** | ***P*** |  | ***M*** | ***SE*** | ***P*** |  | ***M*** | ***SE*** | ***P*** |  | ***M*** | ***SE*** | ***P*** |  | ***M*** | ***SE*** | ***P*** |
| **Post-test score** |  |  |  |  |  |  |  |  |  |  |  |  |  |  |  |  |  |  |  |
| Intercept | 53.04 | 0.63 | 1.00 |  | 53.04 | 0.63 | 1.00 |  | 50.46 | 0.76 | 1.00 |  | 50.46 | 0.76 | 1.00 |  | 50.46 | 0.82 | 1.00 |
| P-I | 0.16 | 0.05 | 0.85 |  | 0.13 | 0.04 | 0.89 |  | 0.13 | 0.04 | 0.91 |  | -0.00 | 0.05 | 0.02 |  | -0.00 | 0.06 | 0.03 |
| P-II | 0.00 | 0.05 | 0.04 |  | 0.00 | 0.04 | 0.04 |  | 0.00 | 0.04 | 0.04 |  | 0.00 | 0.05 | 0.02 |  | 0.00 | 0.06 | 0.02 |
| Pre-test score |  |  |  |  | 0.70 | 0.04 | 1.00 |  | 0.70 | 0.04 | 1.00 |  | 0.69 | 0.04 | 1.00 |  |  |  |  |
| Group |  |  |  |  |  |  |  |  | 5.14 | 1.15 | 1.00 |  | 5.14 | 1.15 | 1.00 |  | 5.15 | 1.32 | 0.99 |
| P-I x Group |  |  |  |  |  |  |  |  |  |  |  |  | 0.27 | 0.08 | 0.93 |  | 0.33 | 0.11 | 0.88 |
| P-II x Group |  |  |  |  |  |  |  |  |  |  |  |  | 0.00 | 0.08 | 0.04 |  | 0.00 | 0.10 | 0.05 |
| **Absolute change score** |  |  |  |  |  |  |  |  |  |  |  |  |  |  |  |  |  |  |  |
| Intercept | 3.04 | 0.57 | 1.00 |  | 3.04 | 0.57 | 1.00 |  | 0.47 | 0.71 | 0.19 |  | 0.47 | 0.71 | 0.19 |  | 0.47 | 0.72 | 0.17 |
| P-I | 0.12 | 0.04 | 0.78 |  | 0.13 | 0.04 | 0.89 |  | 0.13 | 0.04 | 0.91 |  | -0.00 | 0.05 | 0.02 |  | -0.00 | 0.06 | 0.03 |
| P-II | 0.00 | 0.04 | 0.05 |  | 0.00 | 0.04 | 0.04 |  | 0.00 | 0.04 | 0.04 |  | 0.00 | 0.05 | 0.02 |  | 0.00 | 0.05 | 0.02 |
| Pre-test score |  |  |  |  | -0.30 | 0.04 | 1.00 |  | -0.30 | 0.04 | 1.00 |  | -0.31 | 0.04 | 1.00 |  |  |  |  |
| Group |  |  |  |  |  |  |  |  | 5.14 | 1.15 | 1.00 |  | 5.14 | 1.15 | 1.00 |  | 5.14 | 1.18 | 1.00 |
| P-I x Group |  |  |  |  |  |  |  |  |  |  |  |  | 0.27 | 0.08 | 0.93 |  | 0.25 | 0.09 | 0.82 |
| P-II x Group |  |  |  |  |  |  |  |  |  |  |  |  | 0.00 | 0.08 | 0.04 |  | 0.00 | 0.08 | 0.04 |
| **Relative change score** |  |  |  |  |  |  |  |  |  |  |  |  |  |  |  |  |  |  |  |
| Intercept | 7.75 | 1.26 | 1.00 |  | 7.75 | 1.26 | 1.00 |  | 2.79 | 1.56 | 0.56 |  | 2.78 | 1.56 | 0.57 |  | 2.79 | 1.59 | 0.52 |
| P-I | 0.24 | 0.10 | 0.69 |  | 0.28 | 0.09 | 0.86 |  | 0.28 | 0.08 | 0.88 |  | -0.01 | 0.11 | 0.02 |  | -0.01 | 0.13 | 0.03 |
| P-II | 0.01 | 0.10 | 0.04 |  | 0.01 | 0.09 | 0.04 |  | 0.01 | 0.09 | 0.04 |  | 0.01 | 0.11 | 0.01 |  | 0.01 | 0.12 | 0.02 |
| Pre-test score |  |  |  |  | -0.84 | 0.12 | 1.00 |  | -0.84 | 0.11 | 1.00 |  | -0.85 | 0.11 | 1.00 |  |  |  |  |
| Group |  |  |  |  |  |  |  |  | 9.92 | 2.48 | 0.99 |  | 9.92 | 2.48 | 0.99 |  | 9.91 | 2.58 | 0.99 |
| P-I x Group |  |  |  |  |  |  |  |  |  |  |  |  | 0.56 | 0.18 | 0.90 |  | 0.50 | 0.19 | 0.73 |
| P-II x Group |  |  |  |  |  |  |  |  |  |  |  |  | 0.00 | 0.17 | 0.04 |  | -0.00 | 0.18 | 0.04 |
| **Residual score** |  |  |  |  |  |  |  |  |  |  |  |  |  |  |  |  |  |  |  |
| Intercept | -0.00 | 0.00 | 0.00 |  | -0.00 | 0.00 | 0.00 |  | -2.57 | 0.58 | 0.99 |  | -2.57 | 0.57 | 0.99 |  | -2.57 | 0.57 | 0.99 |
| P-I | 0.13 | 0.04 | 0.89 |  | 0.13 | 0.04 | 0.89 |  | 0.13 | 0.04 | 0.91 |  | -0.00 | 0.05 | 0.02 |  | -0.00 | 0.05 | 0.02 |
| P-II | 0.00 | 0.04 | 0.04 |  | 0.00 | 0.04 | 0.04 |  | 0.00 | 0.04 | 0.04 |  | 0.00 | 0.05 | 0.02 |  | 0.00 | 0.05 | 0.02 |
| Pre-test score |  |  |  |  | -0.01 | 0.01 | 0.00 |  | -0.01 | 0.01 | 0.00 |  | -0.01 | 0.02 | 0.00 |  |  |  |  |
| Group |  |  |  |  |  |  |  |  | 5.14 | 1.15 | 1.00 |  | 5.14 | 1.15 | 1.00 |  | 5.13 | 1.15 | 1.00 |
| P-I x Group |  |  |  |  |  |  |  |  |  |  |  |  | 0.27 | 0.08 | 0.93 |  | 0.27 | 0.08 | 0.92 |
| P-II x Group |  |  |  |  |  |  |  |  |  |  |  |  | 0.00 | 0.08 | 0.04 |  | 0.00 | 0.08 | 0.04 |

Table 17: Results of all investigated simulations ordered according to their used model and dependent variable, for a **reliability of .80**, and a sample size of ***n* = 50**

| **Coefficient** | **Model 1** | | |  | **Model 2** | | |  | **Model 3** | | |  | **Model 4** | | |  | **Model 5** | | |
| --- | --- | --- | --- | --- | --- | --- | --- | --- | --- | --- | --- | --- | --- | --- | --- | --- | --- | --- | --- |
|  | ***M*** | ***SE*** | ***P*** |  | ***M*** | ***SE*** | ***P*** |  | ***M*** | ***SE*** | ***P*** |  | ***M*** | ***SE*** | ***P*** |  | ***M*** | ***SE*** | ***P*** |
| **Post-test score** |  |  |  |  |  |  |  |  |  |  |  |  |  |  |  |  |  |  |  |
| Intercept | 53.42 | 2.07 | 1.00 |  | 53.42 | 2.07 | 1.00 |  | 50.50 | 2.53 | 1.00 |  | 50.49 | 2.51 | 1.00 |  | 50.49 | 2.74 | 1.00 |
| P-I | 0.18 | 0.17 | 0.17 |  | 0.15 | 0.13 | 0.19 |  | 0.15 | 0.12 | 0.21 |  | -0.00 | 0.16 | 0.03 |  | 0.01 | 0.21 | 0.02 |
| P-II | -0.00 | 0.17 | 0.05 |  | -0.00 | 0.13 | 0.05 |  | 0.00 | 0.12 | 0.05 |  | -0.00 | 0.16 | 0.02 |  | -0.00 | 0.22 | 0.03 |
| Pre-test score |  |  |  |  | 0.80 | 0.14 | 1.00 |  | 0.80 | 0.13 | 1.00 |  | 0.79 | 0.13 | 1.00 |  |  |  |  |
| Group |  |  |  |  |  |  |  |  | 5.85 | 3.63 | 0.60 |  | 5.85 | 3.63 | 0.60 |  | 5.85 | 4.28 | 0.44 |
| P-I x Group |  |  |  |  |  |  |  |  |  |  |  |  | 0.31 | 0.25 | 0.22 |  | 0.35 | 0.36 | 0.18 |
| P-II x Group |  |  |  |  |  |  |  |  |  |  |  |  | 0.01 | 0.25 | 0.04 |  | 0.01 | 0.34 | 0.03 |
| **Absolute change score** |  |  |  |  |  |  |  |  |  |  |  |  |  |  |  |  |  |  |  |
| Intercept | 3.39 | 1.75 | 0.67 |  | 3.39 | 1.75 | 0.69 |  | 0.47 | 2.30 | 0.15 |  | 0.46 | 2.29 | 0.15 |  | 0.47 | 2.31 | 0.14 |
| P-I | 0.14 | 0.13 | 0.17 |  | 0.15 | 0.13 | 0.19 |  | 0.15 | 0.12 | 0.21 |  | -0.00 | 0.16 | 0.03 |  | -0.00 | 0.17 | 0.03 |
| P-II | 0.00 | 0.13 | 0.04 |  | -0.00 | 0.13 | 0.05 |  | 0.00 | 0.12 | 0.05 |  | -0.00 | 0.16 | 0.02 |  | -0.00 | 0.17 | 0.03 |
| Pre-test score |  |  |  |  | -0.20 | 0.14 | 0.36 |  | -0.20 | 0.13 | 0.41 |  | -0.21 | 0.13 | 0.40 |  |  |  |  |
| Group |  |  |  |  |  |  |  |  | 5.85 | 3.63 | 0.60 |  | 5.85 | 3.63 | 0.60 |  | 5.84 | 3.68 | 0.59 |
| P-I x Group |  |  |  |  |  |  |  |  |  |  |  |  | 0.31 | 0.25 | 0.22 |  | 0.30 | 0.25 | 0.19 |
| P-II x Group |  |  |  |  |  |  |  |  |  |  |  |  | 0.01 | 0.25 | 0.04 |  | 0.01 | 0.26 | 0.05 |
| **Relative change score** |  |  |  |  |  |  |  |  |  |  |  |  |  |  |  |  |  |  |  |
| Intercept | 8.07 | 3.83 | 0.74 |  | 8.07 | 3.83 | 0.77 |  | 2.46 | 4.96 | 0.20 |  | 2.45 | 4.94 | 0.20 |  | 2.46 | 5.01 | 0.17 |
| P-I | 0.29 | 0.29 | 0.15 |  | 0.31 | 0.27 | 0.17 |  | 0.31 | 0.26 | 0.18 |  | -0.01 | 0.35 | 0.03 |  | -0.02 | 0.38 | 0.04 |
| P-II | 0.00 | 0.29 | 0.05 |  | -0.00 | 0.28 | 0.05 |  | 0.00 | 0.27 | 0.05 |  | -0.00 | 0.34 | 0.02 |  | -0.00 | 0.38 | 0.03 |
| Pre-test score |  |  |  |  | -0.62 | 0.36 | 0.56 |  | -0.62 | 0.35 | 0.59 |  | -0.63 | 0.35 | 0.59 |  |  |  |  |
| Group |  |  |  |  |  |  |  |  | 11.23 | 7.66 | 0.55 |  | 11.24 | 7.67 | 0.55 |  | 11.24 | 7.95 | 0.53 |
| P-I x Group |  |  |  |  |  |  |  |  |  |  |  |  | 0.65 | 0.54 | 0.21 |  | 0.62 | 0.57 | 0.17 |
| P-II x Group |  |  |  |  |  |  |  |  |  |  |  |  | 0.02 | 0.53 | 0.04 |  | 0.02 | 0.58 | 0.04 |
| **Residual score** |  |  |  |  |  |  |  |  |  |  |  |  |  |  |  |  |  |  |  |
| Intercept | -0.00 | 0.00 | 0.00 |  | -0.00 | 0.00 | 0.00 |  | -2.92 | 1.81 | 0.40 |  | -2.93 | 1.81 | 0.40 |  | -2.87 | 1.78 | 0.40 |
| P-I | 0.15 | 0.13 | 0.19 |  | 0.15 | 0.13 | 0.19 |  | 0.15 | 0.12 | 0.21 |  | -0.00 | 0.16 | 0.03 |  | -0.00 | 0.16 | 0.02 |
| P-II | -0.00 | 0.12 | 0.05 |  | -0.00 | 0.13 | 0.05 |  | 0.00 | 0.12 | 0.05 |  | -0.00 | 0.16 | 0.02 |  | -0.00 | 0.15 | 0.02 |
| Pre-test score |  |  |  |  | -0.01 | 0.04 | 0.00 |  | -0.01 | 0.06 | 0.00 |  | -0.01 | 0.07 | 0.00 |  |  |  |  |
| Group |  |  |  |  |  |  |  |  | 5.85 | 3.63 | 0.60 |  | 5.85 | 3.63 | 0.60 |  | 5.72 | 3.57 | 0.60 |
| P-I x Group |  |  |  |  |  |  |  |  |  |  |  |  | 0.31 | 0.25 | 0.22 |  | 0.30 | 0.24 | 0.21 |
| P-II x Group |  |  |  |  |  |  |  |  |  |  |  |  | 0.01 | 0.25 | 0.04 |  | 0.01 | 0.24 | 0.04 |

Table 18: Results of all investigated simulations ordered according to their used model and dependent variable, for a **reliability of .80**, and a sample size of ***n* = 100**

| **Coefficient** | **Model 1** | | |  | **Model 2** | | |  | **Model 3** | | |  | **Model 4** | | |  | **Model 5** | | |
| --- | --- | --- | --- | --- | --- | --- | --- | --- | --- | --- | --- | --- | --- | --- | --- | --- | --- | --- | --- |
|  | ***M*** | ***SE*** | ***P*** |  | ***M*** | ***SE*** | ***P*** |  | ***M*** | ***SE*** | ***P*** |  | ***M*** | ***SE*** | ***P*** |  | ***M*** | ***SE*** | ***P*** |
| **Post-test score** |  |  |  |  |  |  |  |  |  |  |  |  |  |  |  |  |  |  |  |
| Intercept | 53.33 | 1.45 | 1.00 |  | 53.33 | 1.45 | 1.00 |  | 50.58 | 1.77 | 1.00 |  | 50.58 | 1.77 | 1.00 |  | 50.59 | 1.96 | 1.00 |
| P-I | 0.18 | 0.13 | 0.32 |  | 0.15 | 0.09 | 0.37 |  | 0.15 | 0.09 | 0.41 |  | -0.00 | 0.11 | 0.03 |  | -0.00 | 0.15 | 0.02 |
| P-II | 0.00 | 0.12 | 0.05 |  | 0.01 | 0.09 | 0.05 |  | 0.00 | 0.08 | 0.05 |  | 0.00 | 0.11 | 0.03 |  | -0.00 | 0.15 | 0.02 |
| Pre-test score |  |  |  |  | 0.79 | 0.10 | 1.00 |  | 0.79 | 0.09 | 1.00 |  | 0.79 | 0.09 | 1.00 |  |  |  |  |
| Group |  |  |  |  |  |  |  |  | 5.51 | 2.46 | 0.81 |  | 5.50 | 2.46 | 0.81 |  | 5.48 | 2.94 | 0.63 |
| P-I x Group |  |  |  |  |  |  |  |  |  |  |  |  | 0.30 | 0.16 | 0.40 |  | 0.37 | 0.24 | 0.33 |
| P-II x Group |  |  |  |  |  |  |  |  |  |  |  |  | -0.00 | 0.17 | 0.06 |  | 0.01 | 0.23 | 0.05 |
| **Absolute change score** |  |  |  |  |  |  |  |  |  |  |  |  |  |  |  |  |  |  |  |
| Intercept | 3.32 | 1.19 | 0.90 |  | 3.32 | 1.19 | 0.90 |  | 0.56 | 1.55 | 0.16 |  | 0.57 | 1.55 | 0.17 |  | 0.56 | 1.56 | 0.15 |
| P-I | 0.14 | 0.10 | 0.31 |  | 0.15 | 0.09 | 0.37 |  | 0.15 | 0.09 | 0.41 |  | -0.00 | 0.11 | 0.03 |  | 0.00 | 0.12 | 0.03 |
| P-II | 0.01 | 0.09 | 0.05 |  | 0.01 | 0.09 | 0.05 |  | 0.00 | 0.08 | 0.05 |  | 0.00 | 0.11 | 0.03 |  | 0.01 | 0.12 | 0.04 |
| Pre-test score |  |  |  |  | -0.21 | 0.10 | 0.62 |  | -0.21 | 0.09 | 0.64 |  | -0.21 | 0.09 | 0.67 |  |  |  |  |
| Group |  |  |  |  |  |  |  |  | 5.51 | 2.46 | 0.81 |  | 5.50 | 2.46 | 0.81 |  | 5.51 | 2.50 | 0.80 |
| P-I x Group |  |  |  |  |  |  |  |  |  |  |  |  | 0.30 | 0.16 | 0.40 |  | 0.29 | 0.16 | 0.36 |
| P-II x Group |  |  |  |  |  |  |  |  |  |  |  |  | -0.00 | 0.17 | 0.06 |  | -0.00 | 0.18 | 0.06 |
| **Relative change score** |  |  |  |  |  |  |  |  |  |  |  |  |  |  |  |  |  |  |  |
| Intercept | 7.89 | 2.64 | 0.94 |  | 7.89 | 2.64 | 0.94 |  | 2.59 | 3.39 | 0.26 |  | 2.59 | 3.40 | 0.26 |  | 2.59 | 3.45 | 0.22 |
| P-I | 0.29 | 0.21 | 0.28 |  | 0.31 | 0.20 | 0.35 |  | 0.31 | 0.19 | 0.39 |  | 0.00 | 0.23 | 0.03 |  | 0.00 | 0.25 | 0.04 |
| P-II | 0.01 | 0.20 | 0.04 |  | 0.01 | 0.19 | 0.04 |  | 0.01 | 0.18 | 0.04 |  | 0.01 | 0.23 | 0.03 |  | 0.01 | 0.26 | 0.04 |
| Pre-test score |  |  |  |  | -0.63 | 0.25 | 0.82 |  | -0.63 | 0.25 | 0.83 |  | -0.64 | 0.25 | 0.85 |  |  |  |  |
| Group |  |  |  |  |  |  |  |  | 10.61 | 5.25 | 0.75 |  | 10.60 | 5.25 | 0.75 |  | 10.61 | 5.43 | 0.72 |
| P-I x Group |  |  |  |  |  |  |  |  |  |  |  |  | 0.62 | 0.35 | 0.38 |  | 0.58 | 0.37 | 0.29 |
| P-II x Group |  |  |  |  |  |  |  |  |  |  |  |  | -0.00 | 0.37 | 0.06 |  | -0.01 | 0.40 | 0.06 |
| **Residual score** |  |  |  |  |  |  |  |  |  |  |  |  |  |  |  |  |  |  |  |
| Intercept | 0.00 | 0.00 | 0.00 |  | 0.00 | 0.00 | 0.00 |  | -2.75 | 1.23 | 0.64 |  | -2.75 | 1.22 | 0.64 |  | -2.72 | 1.21 | 0.64 |
| P-I | 0.15 | 0.09 | 0.37 |  | 0.15 | 0.09 | 0.37 |  | 0.15 | 0.09 | 0.41 |  | -0.00 | 0.11 | 0.03 |  | -0.00 | 0.11 | 0.03 |
| P-II | 0.01 | 0.09 | 0.05 |  | 0.01 | 0.09 | 0.05 |  | 0.00 | 0.08 | 0.05 |  | 0.00 | 0.11 | 0.03 |  | 0.00 | 0.11 | 0.03 |
| Pre-test score |  |  |  |  | -0.01 | 0.02 | 0.00 |  | -0.01 | 0.04 | 0.00 |  | -0.01 | 0.04 | 0.00 |  |  |  |  |
| Group |  |  |  |  |  |  |  |  | 5.51 | 2.46 | 0.81 |  | 5.50 | 2.46 | 0.81 |  | 5.45 | 2.44 | 0.81 |
| P-I x Group |  |  |  |  |  |  |  |  |  |  |  |  | 0.30 | 0.16 | 0.40 |  | 0.30 | 0.16 | 0.40 |
| P-II x Group |  |  |  |  |  |  |  |  |  |  |  |  | -0.00 | 0.17 | 0.06 |  | -0.00 | 0.17 | 0.05 |

Table 19: Results of all investigated simulations ordered according to their used model and dependent variable, for a **reliability of .80**, and a sample size of ***n* = 150**

| **Coefficient** | **Model 1** | | |  | **Model 2** | | |  | **Model 3** | | |  | **Model 4** | | |  | **Model 5** | | |
| --- | --- | --- | --- | --- | --- | --- | --- | --- | --- | --- | --- | --- | --- | --- | --- | --- | --- | --- | --- |
|  | ***M*** | ***SE*** | ***P*** |  | ***M*** | ***SE*** | ***P*** |  | ***M*** | ***SE*** | ***P*** |  | ***M*** | ***SE*** | ***P*** |  | ***M*** | ***SE*** | ***P*** |
| **Post-test score** |  |  |  |  |  |  |  |  |  |  |  |  |  |  |  |  |  |  |  |
| Intercept | 53.31 | 1.17 | 1.00 |  | 53.31 | 1.17 | 1.00 |  | 50.57 | 1.40 | 1.00 |  | 50.57 | 1.39 | 1.00 |  | 50.57 | 1.58 | 1.00 |
| P-I | 0.18 | 0.10 | 0.47 |  | 0.15 | 0.07 | 0.57 |  | 0.15 | 0.06 | 0.64 |  | 0.00 | 0.09 | 0.04 |  | 0.00 | 0.12 | 0.02 |
| P-II | -0.00 | 0.10 | 0.05 |  | -0.00 | 0.07 | 0.04 |  | -0.00 | 0.07 | 0.05 |  | 0.00 | 0.09 | 0.03 |  | 0.00 | 0.12 | 0.03 |
| Pre-test score |  |  |  |  | 0.80 | 0.08 | 1.00 |  | 0.80 | 0.07 | 1.00 |  | 0.79 | 0.07 | 1.00 |  |  |  |  |
| Group |  |  |  |  |  |  |  |  | 5.48 | 1.95 | 0.92 |  | 5.48 | 1.95 | 0.92 |  | 5.50 | 2.35 | 0.78 |
| P-I x Group |  |  |  |  |  |  |  |  |  |  |  |  | 0.30 | 0.13 | 0.62 |  | 0.37 | 0.19 | 0.50 |
| P-II x Group |  |  |  |  |  |  |  |  |  |  |  |  | -0.00 | 0.14 | 0.05 |  | -0.01 | 0.19 | 0.05 |
| **Absolute change score** |  |  |  |  |  |  |  |  |  |  |  |  |  |  |  |  |  |  |  |
| Intercept | 3.31 | 0.99 | 0.97 |  | 3.31 | 0.99 | 0.97 |  | 0.57 | 1.26 | 0.18 |  | 0.57 | 1.25 | 0.18 |  | 0.58 | 1.25 | 0.16 |
| P-I | 0.15 | 0.07 | 0.50 |  | 0.15 | 0.07 | 0.57 |  | 0.15 | 0.06 | 0.64 |  | 0.00 | 0.09 | 0.04 |  | 0.00 | 0.09 | 0.04 |
| P-II | -0.00 | 0.07 | 0.04 |  | -0.00 | 0.07 | 0.04 |  | -0.00 | 0.07 | 0.05 |  | 0.00 | 0.09 | 0.03 |  | 0.00 | 0.09 | 0.03 |
| Pre-test score |  |  |  |  | -0.20 | 0.08 | 0.77 |  | -0.20 | 0.07 | 0.80 |  | -0.21 | 0.07 | 0.82 |  |  |  |  |
| Group |  |  |  |  |  |  |  |  | 5.48 | 1.95 | 0.92 |  | 5.48 | 1.95 | 0.92 |  | 5.47 | 1.96 | 0.91 |
| P-I x Group |  |  |  |  |  |  |  |  |  |  |  |  | 0.30 | 0.13 | 0.62 |  | 0.29 | 0.14 | 0.52 |
| P-II x Group |  |  |  |  |  |  |  |  |  |  |  |  | -0.00 | 0.14 | 0.05 |  | -0.00 | 0.14 | 0.05 |
| **Relative change score** |  |  |  |  |  |  |  |  |  |  |  |  |  |  |  |  |  |  |  |
| Intercept | 7.85 | 2.17 | 0.99 |  | 7.85 | 2.17 | 0.99 |  | 2.59 | 2.74 | 0.30 |  | 2.59 | 2.72 | 0.30 |  | 2.60 | 2.73 | 0.27 |
| P-I | 0.29 | 0.16 | 0.42 |  | 0.31 | 0.15 | 0.52 |  | 0.32 | 0.14 | 0.56 |  | 0.00 | 0.19 | 0.04 |  | 0.00 | 0.21 | 0.04 |
| P-II | -0.00 | 0.16 | 0.04 |  | -0.00 | 0.15 | 0.04 |  | -0.00 | 0.14 | 0.04 |  | 0.00 | 0.19 | 0.03 |  | 0.00 | 0.20 | 0.03 |
| Pre-test score |  |  |  |  | -0.62 | 0.20 | 0.93 |  | -0.62 | 0.20 | 0.94 |  | -0.63 | 0.20 | 0.96 |  |  |  |  |
| Group |  |  |  |  |  |  |  |  | 10.53 | 4.15 | 0.87 |  | 10.53 | 4.14 | 0.87 |  | 10.51 | 4.25 | 0.84 |
| P-I x Group |  |  |  |  |  |  |  |  |  |  |  |  | 0.63 | 0.29 | 0.57 |  | 0.58 | 0.30 | 0.44 |
| P-II x Group |  |  |  |  |  |  |  |  |  |  |  |  | -0.01 | 0.30 | 0.05 |  | -0.00 | 0.31 | 0.05 |
| **Residual score** |  |  |  |  |  |  |  |  |  |  |  |  |  |  |  |  |  |  |  |
| Intercept | 0.00 | 0.00 | 0.00 |  | 0.00 | 0.00 | 0.00 |  | -2.74 | 0.97 | 0.81 |  | -2.74 | 0.97 | 0.81 |  | -2.72 | 0.96 | 0.81 |
| P-I | 0.15 | 0.07 | 0.57 |  | 0.15 | 0.07 | 0.57 |  | 0.15 | 0.06 | 0.64 |  | 0.00 | 0.09 | 0.04 |  | 0.00 | 0.09 | 0.04 |
| P-II | -0.00 | 0.07 | 0.05 |  | -0.00 | 0.07 | 0.04 |  | -0.00 | 0.07 | 0.05 |  | 0.00 | 0.09 | 0.03 |  | 0.00 | 0.09 | 0.03 |
| Pre-test score |  |  |  |  | -0.01 | 0.02 | 0.00 |  | -0.01 | 0.03 | 0.00 |  | -0.01 | 0.03 | 0.00 |  |  |  |  |
| Group |  |  |  |  |  |  |  |  | 5.48 | 1.95 | 0.92 |  | 5.48 | 1.95 | 0.92 |  | 5.44 | 1.93 | 0.92 |
| P-I x Group |  |  |  |  |  |  |  |  |  |  |  |  | 0.30 | 0.13 | 0.62 |  | 0.30 | 0.13 | 0.62 |
| P-II x Group |  |  |  |  |  |  |  |  |  |  |  |  | -0.00 | 0.14 | 0.05 |  | -0.00 | 0.14 | 0.05 |

Table 20: Results of all investigated simulations ordered according to their used model and dependent variable, for a **reliability of .80**, and a sample size of ***n* = 200**

| **Coefficient** | **Model 1** | | |  | **Model 2** | | |  | **Model 3** | | |  | **Model 4** | | |  | **Model 5** | | |
| --- | --- | --- | --- | --- | --- | --- | --- | --- | --- | --- | --- | --- | --- | --- | --- | --- | --- | --- | --- |
|  | ***M*** | ***SE*** | ***P*** |  | ***M*** | ***SE*** | ***P*** |  | ***M*** | ***SE*** | ***P*** |  | ***M*** | ***SE*** | ***P*** |  | ***M*** | ***SE*** | ***P*** |
| **Post-test score** |  |  |  |  |  |  |  |  |  |  |  |  |  |  |  |  |  |  |  |
| Intercept | 53.29 | 1.03 | 1.00 |  | 53.29 | 1.03 | 1.00 |  | 50.49 | 1.24 | 1.00 |  | 50.48 | 1.24 | 1.00 |  | 50.47 | 1.36 | 1.00 |
| P-I | 0.18 | 0.09 | 0.57 |  | 0.15 | 0.06 | 0.65 |  | 0.15 | 0.06 | 0.71 |  | 0.00 | 0.07 | 0.02 |  | 0.01 | 0.10 | 0.02 |
| P-II | -0.00 | 0.08 | 0.04 |  | 0.00 | 0.06 | 0.05 |  | 0.00 | 0.06 | 0.05 |  | -0.00 | 0.08 | 0.03 |  | -0.01 | 0.10 | 0.03 |
| Pre-test score |  |  |  |  | 0.80 | 0.07 | 1.00 |  | 0.80 | 0.07 | 1.00 |  | 0.79 | 0.06 | 1.00 |  |  |  |  |
| Group |  |  |  |  |  |  |  |  | 5.61 | 1.77 | 0.96 |  | 5.61 | 1.78 | 0.96 |  | 5.64 | 2.09 | 0.87 |
| P-I x Group |  |  |  |  |  |  |  |  |  |  |  |  | 0.30 | 0.11 | 0.71 |  | 0.36 | 0.17 | 0.59 |
| P-II x Group |  |  |  |  |  |  |  |  |  |  |  |  | 0.01 | 0.12 | 0.04 |  | 0.01 | 0.16 | 0.05 |
| **Absolute change score** |  |  |  |  |  |  |  |  |  |  |  |  |  |  |  |  |  |  |  |
| Intercept | 3.26 | 0.89 | 0.99 |  | 3.26 | 0.89 | 0.99 |  | 0.46 | 1.13 | 0.19 |  | 0.45 | 1.12 | 0.20 |  | 0.46 | 1.13 | 0.18 |
| P-I | 0.14 | 0.06 | 0.57 |  | 0.15 | 0.06 | 0.65 |  | 0.15 | 0.06 | 0.71 |  | 0.00 | 0.07 | 0.02 |  | 0.00 | 0.08 | 0.02 |
| P-II | 0.00 | 0.06 | 0.05 |  | 0.00 | 0.06 | 0.05 |  | 0.00 | 0.06 | 0.05 |  | -0.00 | 0.08 | 0.03 |  | 0.00 | 0.08 | 0.04 |
| Pre-test score |  |  |  |  | -0.20 | 0.07 | 0.85 |  | -0.20 | 0.07 | 0.87 |  | -0.21 | 0.06 | 0.89 |  |  |  |  |
| Group |  |  |  |  |  |  |  |  | 5.61 | 1.77 | 0.96 |  | 5.61 | 1.78 | 0.96 |  | 5.60 | 1.80 | 0.96 |
| P-I x Group |  |  |  |  |  |  |  |  |  |  |  |  | 0.30 | 0.11 | 0.71 |  | 0.28 | 0.12 | 0.63 |
| P-II x Group |  |  |  |  |  |  |  |  |  |  |  |  | 0.01 | 0.12 | 0.04 |  | 0.00 | 0.12 | 0.04 |
| **Relative change score** |  |  |  |  |  |  |  |  |  |  |  |  |  |  |  |  |  |  |  |
| Intercept | 7.74 | 1.94 | 1.00 |  | 7.74 | 1.94 | 1.00 |  | 2.35 | 2.42 | 0.32 |  | 2.35 | 2.41 | 0.33 |  | 2.36 | 2.44 | 0.30 |
| P-I | 0.28 | 0.14 | 0.50 |  | 0.31 | 0.13 | 0.62 |  | 0.31 | 0.12 | 0.67 |  | 0.00 | 0.16 | 0.02 |  | 0.00 | 0.18 | 0.02 |
| P-II | 0.01 | 0.14 | 0.05 |  | 0.01 | 0.13 | 0.05 |  | 0.01 | 0.13 | 0.05 |  | -0.00 | 0.16 | 0.03 |  | 0.00 | 0.18 | 0.04 |
| Pre-test score |  |  |  |  | -0.61 | 0.18 | 0.97 |  | -0.62 | 0.17 | 0.98 |  | -0.63 | 0.17 | 0.99 |  |  |  |  |
| Group |  |  |  |  |  |  |  |  | 10.77 | 3.80 | 0.93 |  | 10.77 | 3.81 | 0.93 |  | 10.75 | 3.91 | 0.92 |
| P-I x Group |  |  |  |  |  |  |  |  |  |  |  |  | 0.61 | 0.24 | 0.66 |  | 0.57 | 0.26 | 0.55 |
| P-II x Group |  |  |  |  |  |  |  |  |  |  |  |  | 0.01 | 0.25 | 0.04 |  | 0.01 | 0.26 | 0.04 |
| **Residual score** |  |  |  |  |  |  |  |  |  |  |  |  |  |  |  |  |  |  |  |
| Intercept | 0.00 | 0.00 | 0.00 |  | 0.00 | 0.00 | 0.00 |  | -2.80 | 0.89 | 0.90 |  | -2.81 | 0.88 | 0.90 |  | -2.79 | 0.88 | 0.90 |
| P-I | 0.15 | 0.06 | 0.65 |  | 0.15 | 0.06 | 0.65 |  | 0.15 | 0.06 | 0.71 |  | 0.00 | 0.07 | 0.02 |  | 0.00 | 0.07 | 0.02 |
| P-II | 0.00 | 0.06 | 0.05 |  | 0.00 | 0.06 | 0.05 |  | 0.00 | 0.06 | 0.05 |  | -0.00 | 0.08 | 0.03 |  | 0.00 | 0.08 | 0.03 |
| Pre-test score |  |  |  |  | -0.01 | 0.01 | 0.00 |  | -0.01 | 0.02 | 0.00 |  | -0.01 | 0.03 | 0.00 |  |  |  |  |
| Group |  |  |  |  |  |  |  |  | 5.61 | 1.77 | 0.96 |  | 5.61 | 1.78 | 0.96 |  | 5.58 | 1.77 | 0.96 |
| P-I x Group |  |  |  |  |  |  |  |  |  |  |  |  | 0.30 | 0.11 | 0.71 |  | 0.29 | 0.11 | 0.71 |
| P-II x Group |  |  |  |  |  |  |  |  |  |  |  |  | 0.01 | 0.12 | 0.04 |  | 0.00 | 0.11 | 0.04 |

Table 21: Results of all investigated simulations ordered according to their used model and dependent variable, for a **reliability of .80**, and a sample size of ***n* = 250**

| **Coefficient** | **Model 1** | | |  | **Model 2** | | |  | **Model 3** | | |  | **Model 4** | | |  | **Model 5** | | |
| --- | --- | --- | --- | --- | --- | --- | --- | --- | --- | --- | --- | --- | --- | --- | --- | --- | --- | --- | --- |
|  | ***M*** | ***SE*** | ***P*** |  | ***M*** | ***SE*** | ***P*** |  | ***M*** | ***SE*** | ***P*** |  | ***M*** | ***SE*** | ***P*** |  | ***M*** | ***SE*** | ***P*** |
| **Post-test score** |  |  |  |  |  |  |  |  |  |  |  |  |  |  |  |  |  |  |  |
| Intercept | 53.25 | 0.90 | 1.00 |  | 53.25 | 0.90 | 1.00 |  | 50.47 | 1.04 | 1.00 |  | 50.47 | 1.04 | 1.00 |  | 50.45 | 1.13 | 1.00 |
| P-I | 0.18 | 0.08 | 0.68 |  | 0.15 | 0.05 | 0.77 |  | 0.15 | 0.05 | 0.83 |  | -0.00 | 0.06 | 0.01 |  | 0.00 | 0.09 | 0.03 |
| P-II | -0.00 | 0.07 | 0.05 |  | -0.00 | 0.05 | 0.04 |  | -0.00 | 0.05 | 0.04 |  | -0.00 | 0.07 | 0.03 |  | -0.00 | 0.09 | 0.03 |
| Pre-test score |  |  |  |  | 0.80 | 0.06 | 1.00 |  | 0.80 | 0.06 | 1.00 |  | 0.79 | 0.06 | 1.00 |  |  |  |  |
| Group |  |  |  |  |  |  |  |  | 5.57 | 1.54 | 0.99 |  | 5.57 | 1.54 | 0.99 |  | 5.60 | 1.82 | 0.94 |
| P-I x Group |  |  |  |  |  |  |  |  |  |  |  |  | 0.30 | 0.09 | 0.85 |  | 0.37 | 0.14 | 0.72 |
| P-II x Group |  |  |  |  |  |  |  |  |  |  |  |  | -0.00 | 0.10 | 0.05 |  | -0.00 | 0.14 | 0.04 |
| **Absolute change score** |  |  |  |  |  |  |  |  |  |  |  |  |  |  |  |  |  |  |  |
| Intercept | 3.23 | 0.76 | 0.99 |  | 3.23 | 0.76 | 0.99 |  | 0.45 | 0.94 | 0.17 |  | 0.45 | 0.94 | 0.17 |  | 0.45 | 0.95 | 0.16 |
| P-I | 0.14 | 0.05 | 0.70 |  | 0.15 | 0.05 | 0.77 |  | 0.15 | 0.05 | 0.83 |  | -0.00 | 0.06 | 0.01 |  | -0.00 | 0.07 | 0.02 |
| P-II | -0.00 | 0.05 | 0.04 |  | -0.00 | 0.05 | 0.04 |  | -0.00 | 0.05 | 0.04 |  | -0.00 | 0.07 | 0.03 |  | -0.00 | 0.07 | 0.04 |
| Pre-test score |  |  |  |  | -0.20 | 0.06 | 0.92 |  | -0.20 | 0.06 | 0.94 |  | -0.21 | 0.06 | 0.96 |  |  |  |  |
| Group |  |  |  |  |  |  |  |  | 5.57 | 1.54 | 0.99 |  | 5.57 | 1.54 | 0.99 |  | 5.56 | 1.56 | 0.98 |
| P-I x Group |  |  |  |  |  |  |  |  |  |  |  |  | 0.30 | 0.09 | 0.85 |  | 0.29 | 0.10 | 0.79 |
| P-II x Group |  |  |  |  |  |  |  |  |  |  |  |  | -0.00 | 0.10 | 0.05 |  | 0.00 | 0.11 | 0.05 |
| **Relative change score** |  |  |  |  |  |  |  |  |  |  |  |  |  |  |  |  |  |  |  |
| Intercept | 7.68 | 1.62 | 1.00 |  | 7.68 | 1.62 | 1.00 |  | 2.35 | 2.02 | 0.35 |  | 2.35 | 2.01 | 0.36 |  | 2.36 | 2.04 | 0.32 |
| P-I | 0.28 | 0.11 | 0.62 |  | 0.31 | 0.11 | 0.74 |  | 0.31 | 0.10 | 0.80 |  | -0.00 | 0.14 | 0.02 |  | -0.00 | 0.15 | 0.02 |
| P-II | -0.00 | 0.12 | 0.03 |  | -0.00 | 0.11 | 0.04 |  | -0.00 | 0.11 | 0.04 |  | -0.00 | 0.14 | 0.03 |  | -0.00 | 0.16 | 0.04 |
| Pre-test score |  |  |  |  | -0.62 | 0.15 | 0.99 |  | -0.62 | 0.15 | 1.00 |  | -0.63 | 0.14 | 1.00 |  |  |  |  |
| Group |  |  |  |  |  |  |  |  | 10.66 | 3.26 | 0.96 |  | 10.66 | 3.26 | 0.97 |  | 10.64 | 3.37 | 0.96 |
| P-I x Group |  |  |  |  |  |  |  |  |  |  |  |  | 0.62 | 0.21 | 0.81 |  | 0.57 | 0.22 | 0.68 |
| P-II x Group |  |  |  |  |  |  |  |  |  |  |  |  | 0.00 | 0.22 | 0.05 |  | 0.00 | 0.24 | 0.05 |
| **Residual score** |  |  |  |  |  |  |  |  |  |  |  |  |  |  |  |  |  |  |  |
| Intercept | -0.00 | 0.00 | 0.00 |  | -0.00 | 0.00 | 0.00 |  | -2.78 | 0.77 | 0.96 |  | -2.78 | 0.76 | 0.96 |  | -2.77 | 0.76 | 0.96 |
| P-I | 0.15 | 0.05 | 0.77 |  | 0.15 | 0.05 | 0.77 |  | 0.15 | 0.05 | 0.83 |  | -0.00 | 0.06 | 0.01 |  | -0.00 | 0.06 | 0.01 |
| P-II | -0.00 | 0.05 | 0.04 |  | -0.00 | 0.05 | 0.04 |  | -0.00 | 0.05 | 0.04 |  | -0.00 | 0.07 | 0.03 |  | -0.00 | 0.07 | 0.03 |
| Pre-test score |  |  |  |  | -0.01 | 0.01 | 0.00 |  | -0.01 | 0.02 | 0.00 |  | -0.01 | 0.03 | 0.00 |  |  |  |  |
| Group |  |  |  |  |  |  |  |  | 5.57 | 1.54 | 0.99 |  | 5.57 | 1.54 | 0.99 |  | 5.54 | 1.54 | 0.99 |
| P-I x Group |  |  |  |  |  |  |  |  |  |  |  |  | 0.30 | 0.09 | 0.85 |  | 0.30 | 0.09 | 0.84 |
| P-II x Group |  |  |  |  |  |  |  |  |  |  |  |  | -0.00 | 0.10 | 0.05 |  | -0.00 | 0.10 | 0.05 |

Table 22: Results of all investigated simulations ordered according to their used model and dependent variable, for a **reliability of .80**, and a sample size of ***n* = 300**

| **Coefficient** | **Model 1** | | |  | **Model 2** | | |  | **Model 3** | | |  | **Model 4** | | |  | **Model 5** | | |
| --- | --- | --- | --- | --- | --- | --- | --- | --- | --- | --- | --- | --- | --- | --- | --- | --- | --- | --- | --- |
|  | ***M*** | ***SE*** | ***P*** |  | ***M*** | ***SE*** | ***P*** |  | ***M*** | ***SE*** | ***P*** |  | ***M*** | ***SE*** | ***P*** |  | ***M*** | ***SE*** | ***P*** |
| **Post-test score** |  |  |  |  |  |  |  |  |  |  |  |  |  |  |  |  |  |  |  |
| Intercept | 53.24 | 0.86 | 1.00 |  | 53.24 | 0.86 | 1.00 |  | 50.51 | 1.02 | 1.00 |  | 50.51 | 1.02 | 1.00 |  | 50.50 | 1.12 | 1.00 |
| P-I | 0.18 | 0.07 | 0.77 |  | 0.15 | 0.05 | 0.86 |  | 0.15 | 0.04 | 0.89 |  | 0.00 | 0.06 | 0.01 |  | -0.00 | 0.08 | 0.03 |
| P-II | -0.00 | 0.07 | 0.05 |  | 0.00 | 0.05 | 0.04 |  | 0.00 | 0.05 | 0.05 |  | 0.00 | 0.06 | 0.03 |  | -0.00 | 0.08 | 0.02 |
| Pre-test score |  |  |  |  | 0.79 | 0.06 | 1.00 |  | 0.79 | 0.05 | 1.00 |  | 0.79 | 0.05 | 1.00 |  |  |  |  |
| Group |  |  |  |  |  |  |  |  | 5.45 | 1.44 | 0.99 |  | 5.45 | 1.44 | 0.99 |  | 5.47 | 1.73 | 0.94 |
| P-I x Group |  |  |  |  |  |  |  |  |  |  |  |  | 0.30 | 0.09 | 0.90 |  | 0.37 | 0.13 | 0.80 |
| P-II x Group |  |  |  |  |  |  |  |  |  |  |  |  | 0.00 | 0.10 | 0.04 |  | 0.00 | 0.13 | 0.03 |
| **Absolute change score** |  |  |  |  |  |  |  |  |  |  |  |  |  |  |  |  |  |  |  |
| Intercept | 3.23 | 0.72 | 1.00 |  | 3.23 | 0.72 | 1.00 |  | 0.51 | 0.90 | 0.20 |  | 0.51 | 0.90 | 0.21 |  | 0.51 | 0.91 | 0.20 |
| P-I | 0.14 | 0.05 | 0.81 |  | 0.15 | 0.05 | 0.86 |  | 0.15 | 0.04 | 0.89 |  | 0.00 | 0.06 | 0.01 |  | 0.00 | 0.06 | 0.03 |
| P-II | 0.00 | 0.05 | 0.05 |  | 0.00 | 0.05 | 0.04 |  | 0.00 | 0.05 | 0.05 |  | 0.00 | 0.06 | 0.03 |  | 0.00 | 0.07 | 0.04 |
| Pre-test score |  |  |  |  | -0.21 | 0.06 | 0.96 |  | -0.21 | 0.05 | 0.97 |  | -0.21 | 0.05 | 0.98 |  |  |  |  |
| Group |  |  |  |  |  |  |  |  | 5.45 | 1.44 | 0.99 |  | 5.45 | 1.44 | 0.99 |  | 5.45 | 1.45 | 0.99 |
| P-I x Group |  |  |  |  |  |  |  |  |  |  |  |  | 0.30 | 0.09 | 0.90 |  | 0.28 | 0.09 | 0.83 |
| P-II x Group |  |  |  |  |  |  |  |  |  |  |  |  | 0.00 | 0.10 | 0.04 |  | 0.00 | 0.10 | 0.05 |
| **Relative change score** |  |  |  |  |  |  |  |  |  |  |  |  |  |  |  |  |  |  |  |
| Intercept | 7.69 | 1.56 | 1.00 |  | 7.69 | 1.56 | 1.00 |  | 2.48 | 1.94 | 0.43 |  | 2.47 | 1.95 | 0.43 |  | 2.48 | 1.98 | 0.39 |
| P-I | 0.29 | 0.11 | 0.73 |  | 0.31 | 0.10 | 0.84 |  | 0.31 | 0.10 | 0.87 |  | 0.00 | 0.12 | 0.02 |  | 0.01 | 0.14 | 0.03 |
| P-II | 0.00 | 0.12 | 0.05 |  | 0.00 | 0.11 | 0.05 |  | 0.00 | 0.11 | 0.05 |  | 0.00 | 0.13 | 0.04 |  | 0.00 | 0.15 | 0.04 |
| Pre-test score |  |  |  |  | -0.62 | 0.16 | 1.00 |  | -0.62 | 0.16 | 1.00 |  | -0.63 | 0.16 | 1.00 |  |  |  |  |
| Group |  |  |  |  |  |  |  |  | 10.42 | 3.13 | 0.98 |  | 10.42 | 3.13 | 0.98 |  | 10.41 | 3.20 | 0.98 |
| P-I x Group |  |  |  |  |  |  |  |  |  |  |  |  | 0.61 | 0.19 | 0.86 |  | 0.56 | 0.21 | 0.74 |
| P-II x Group |  |  |  |  |  |  |  |  |  |  |  |  | 0.01 | 0.21 | 0.05 |  | 0.01 | 0.23 | 0.05 |
| **Residual score** |  |  |  |  |  |  |  |  |  |  |  |  |  |  |  |  |  |  |  |
| Intercept | -0.00 | 0.00 | 0.00 |  | -0.00 | 0.00 | 0.00 |  | -2.72 | 0.72 | 0.98 |  | -2.72 | 0.72 | 0.98 |  | -2.72 | 0.72 | 0.98 |
| P-I | 0.15 | 0.05 | 0.86 |  | 0.15 | 0.05 | 0.86 |  | 0.15 | 0.04 | 0.89 |  | 0.00 | 0.06 | 0.01 |  | 0.00 | 0.06 | 0.02 |
| P-II | 0.00 | 0.05 | 0.04 |  | 0.00 | 0.05 | 0.04 |  | 0.00 | 0.05 | 0.05 |  | 0.00 | 0.06 | 0.03 |  | 0.00 | 0.06 | 0.03 |
| Pre-test score |  |  |  |  | -0.01 | 0.01 | 0.00 |  | -0.01 | 0.02 | 0.00 |  | -0.01 | 0.02 | 0.00 |  |  |  |  |
| Group |  |  |  |  |  |  |  |  | 5.45 | 1.44 | 0.99 |  | 5.45 | 1.44 | 0.99 |  | 5.43 | 1.44 | 0.99 |
| P-I x Group |  |  |  |  |  |  |  |  |  |  |  |  | 0.30 | 0.09 | 0.90 |  | 0.30 | 0.09 | 0.89 |
| P-II x Group |  |  |  |  |  |  |  |  |  |  |  |  | 0.00 | 0.10 | 0.04 |  | 0.00 | 0.10 | 0.04 |

Table 23: Results of all investigated simulations ordered according to their used model and dependent variable, for a **reliability of .80**, and a sample size of ***n* = 400**

| **Coefficient** | **Model 1** | | |  | **Model 2** | | |  | **Model 3** | | |  | **Model 4** | | |  | **Model 5** | | |
| --- | --- | --- | --- | --- | --- | --- | --- | --- | --- | --- | --- | --- | --- | --- | --- | --- | --- | --- | --- |
|  | ***M*** | ***SE*** | ***P*** |  | ***M*** | ***SE*** | ***P*** |  | ***M*** | ***SE*** | ***P*** |  | ***M*** | ***SE*** | ***P*** |  | ***M*** | ***SE*** | ***P*** |
| **Post-test score** |  |  |  |  |  |  |  |  |  |  |  |  |  |  |  |  |  |  |  |
| Intercept | 53.27 | 0.74 | 1.00 |  | 53.27 | 0.74 | 1.00 |  | 50.51 | 0.86 | 1.00 |  | 50.51 | 0.85 | 1.00 |  | 50.51 | 0.93 | 1.00 |
| P-I | 0.18 | 0.06 | 0.86 |  | 0.15 | 0.04 | 0.94 |  | 0.15 | 0.04 | 0.96 |  | 0.00 | 0.05 | 0.02 |  | 0.00 | 0.07 | 0.02 |
| P-II | 0.00 | 0.06 | 0.05 |  | -0.00 | 0.04 | 0.06 |  | -0.00 | 0.04 | 0.05 |  | 0.00 | 0.05 | 0.03 |  | 0.00 | 0.07 | 0.03 |
| Pre-test score |  |  |  |  | 0.80 | 0.05 | 1.00 |  | 0.80 | 0.05 | 1.00 |  | 0.79 | 0.05 | 1.00 |  |  |  |  |
| Group |  |  |  |  |  |  |  |  | 5.51 | 1.19 | 1.00 |  | 5.51 | 1.19 | 1.00 |  | 5.51 | 1.43 | 0.98 |
| P-I x Group |  |  |  |  |  |  |  |  |  |  |  |  | 0.30 | 0.08 | 0.96 |  | 0.37 | 0.12 | 0.88 |
| P-II x Group |  |  |  |  |  |  |  |  |  |  |  |  | -0.00 | 0.08 | 0.06 |  | -0.01 | 0.12 | 0.06 |
| **Absolute change score** |  |  |  |  |  |  |  |  |  |  |  |  |  |  |  |  |  |  |  |
| Intercept | 3.26 | 0.63 | 1.00 |  | 3.26 | 0.63 | 1.00 |  | 0.50 | 0.76 | 0.22 |  | 0.50 | 0.75 | 0.23 |  | 0.50 | 0.76 | 0.21 |
| P-I | 0.14 | 0.04 | 0.88 |  | 0.15 | 0.04 | 0.94 |  | 0.15 | 0.04 | 0.96 |  | 0.00 | 0.05 | 0.02 |  | 0.00 | 0.06 | 0.03 |
| P-II | 0.00 | 0.05 | 0.05 |  | -0.00 | 0.04 | 0.06 |  | -0.00 | 0.04 | 0.05 |  | 0.00 | 0.05 | 0.03 |  | -0.00 | 0.06 | 0.04 |
| Pre-test score |  |  |  |  | -0.20 | 0.05 | 0.99 |  | -0.20 | 0.05 | 0.99 |  | -0.21 | 0.05 | 1.00 |  |  |  |  |
| Group |  |  |  |  |  |  |  |  | 5.51 | 1.19 | 1.00 |  | 5.51 | 1.19 | 1.00 |  | 5.51 | 1.20 | 1.00 |
| P-I x Group |  |  |  |  |  |  |  |  |  |  |  |  | 0.30 | 0.08 | 0.96 |  | 0.28 | 0.08 | 0.92 |
| P-II x Group |  |  |  |  |  |  |  |  |  |  |  |  | -0.00 | 0.08 | 0.06 |  | -0.00 | 0.09 | 0.06 |
| **Relative change score** |  |  |  |  |  |  |  |  |  |  |  |  |  |  |  |  |  |  |  |
| Intercept | 7.73 | 1.36 | 1.00 |  | 7.73 | 1.36 | 1.00 |  | 2.44 | 1.65 | 0.50 |  | 2.44 | 1.64 | 0.50 |  | 2.45 | 1.66 | 0.47 |
| P-I | 0.28 | 0.10 | 0.81 |  | 0.31 | 0.09 | 0.92 |  | 0.31 | 0.09 | 0.94 |  | 0.00 | 0.11 | 0.02 |  | 0.00 | 0.12 | 0.03 |
| P-II | -0.00 | 0.10 | 0.05 |  | -0.00 | 0.10 | 0.05 |  | -0.00 | 0.09 | 0.05 |  | -0.00 | 0.12 | 0.03 |  | -0.00 | 0.13 | 0.04 |
| Pre-test score |  |  |  |  | -0.62 | 0.13 | 1.00 |  | -0.62 | 0.12 | 1.00 |  | -0.63 | 0.12 | 1.00 |  |  |  |  |
| Group |  |  |  |  |  |  |  |  | 10.57 | 2.56 | 1.00 |  | 10.57 | 2.56 | 1.00 |  | 10.57 | 2.60 | 1.00 |
| P-I x Group |  |  |  |  |  |  |  |  |  |  |  |  | 0.62 | 0.17 | 0.94 |  | 0.56 | 0.18 | 0.86 |
| P-II x Group |  |  |  |  |  |  |  |  |  |  |  |  | -0.00 | 0.18 | 0.06 |  | 0.00 | 0.19 | 0.06 |
| **Residual score** |  |  |  |  |  |  |  |  |  |  |  |  |  |  |  |  |  |  |  |
| Intercept | -0.00 | 0.00 | 0.00 |  | -0.00 | 0.00 | 0.00 |  | -2.75 | 0.60 | 1.00 |  | -2.75 | 0.59 | 1.00 |  | -2.75 | 0.59 | 1.00 |
| P-I | 0.15 | 0.04 | 0.94 |  | 0.15 | 0.04 | 0.94 |  | 0.15 | 0.04 | 0.96 |  | 0.00 | 0.05 | 0.02 |  | 0.00 | 0.05 | 0.02 |
| P-II | -0.00 | 0.04 | 0.05 |  | -0.00 | 0.04 | 0.06 |  | -0.00 | 0.04 | 0.05 |  | 0.00 | 0.05 | 0.03 |  | 0.00 | 0.05 | 0.04 |
| Pre-test score |  |  |  |  | -0.01 | 0.01 | 0.00 |  | -0.01 | 0.02 | 0.00 |  | -0.01 | 0.02 | 0.00 |  |  |  |  |
| Group |  |  |  |  |  |  |  |  | 5.51 | 1.19 | 1.00 |  | 5.51 | 1.19 | 1.00 |  | 5.50 | 1.19 | 1.00 |
| P-I x Group |  |  |  |  |  |  |  |  |  |  |  |  | 0.30 | 0.08 | 0.96 |  | 0.30 | 0.08 | 0.96 |
| P-II x Group |  |  |  |  |  |  |  |  |  |  |  |  | -0.00 | 0.08 | 0.06 |  | -0.00 | 0.08 | 0.06 |

Table 24: Results of all investigated simulations ordered according to their used model and dependent variable, for a **reliability of .80**, and a sample size of ***n* = 500**

| **Coefficient** | **Model 1** | | |  | **Model 2** | | |  | **Model 3** | | |  | **Model 4** | | |  | **Model 5** | | |
| --- | --- | --- | --- | --- | --- | --- | --- | --- | --- | --- | --- | --- | --- | --- | --- | --- | --- | --- | --- |
|  | ***M*** | ***SE*** | ***P*** |  | ***M*** | ***SE*** | ***P*** |  | ***M*** | ***SE*** | ***P*** |  | ***M*** | ***SE*** | ***P*** |  | ***M*** | ***SE*** | ***P*** |
| **Post-test score** |  |  |  |  |  |  |  |  |  |  |  |  |  |  |  |  |  |  |  |
| Intercept | 53.30 | 0.64 | 1.00 |  | 53.30 | 0.64 | 1.00 |  | 50.53 | 0.74 | 1.00 |  | 50.53 | 0.74 | 1.00 |  | 50.52 | 0.79 | 1.00 |
| P-I | 0.19 | 0.06 | 0.93 |  | 0.15 | 0.04 | 0.97 |  | 0.15 | 0.04 | 0.98 |  | 0.00 | 0.05 | 0.02 |  | 0.00 | 0.06 | 0.02 |
| P-II | -0.00 | 0.05 | 0.05 |  | -0.00 | 0.04 | 0.06 |  | -0.00 | 0.04 | 0.05 |  | -0.00 | 0.05 | 0.02 |  | -0.00 | 0.06 | 0.02 |
| Pre-test score |  |  |  |  | 0.80 | 0.04 | 1.00 |  | 0.80 | 0.04 | 1.00 |  | 0.79 | 0.04 | 1.00 |  |  |  |  |
| Group |  |  |  |  |  |  |  |  | 5.53 | 1.04 | 1.00 |  | 5.53 | 1.04 | 1.00 |  | 5.56 | 1.23 | 1.00 |
| P-I x Group |  |  |  |  |  |  |  |  |  |  |  |  | 0.30 | 0.07 | 0.98 |  | 0.37 | 0.11 | 0.94 |
| P-II x Group |  |  |  |  |  |  |  |  |  |  |  |  | -0.00 | 0.07 | 0.06 |  | -0.00 | 0.10 | 0.05 |
| **Absolute change score** |  |  |  |  |  |  |  |  |  |  |  |  |  |  |  |  |  |  |  |
| Intercept | 3.28 | 0.55 | 1.00 |  | 3.28 | 0.55 | 1.00 |  | 0.51 | 0.66 | 0.22 |  | 0.51 | 0.66 | 0.23 |  | 0.52 | 0.67 | 0.23 |
| P-I | 0.14 | 0.04 | 0.94 |  | 0.15 | 0.04 | 0.97 |  | 0.15 | 0.04 | 0.98 |  | 0.00 | 0.05 | 0.02 |  | 0.00 | 0.05 | 0.02 |
| P-II | -0.00 | 0.04 | 0.06 |  | -0.00 | 0.04 | 0.06 |  | -0.00 | 0.04 | 0.05 |  | -0.00 | 0.05 | 0.02 |  | -0.00 | 0.05 | 0.03 |
| Pre-test score |  |  |  |  | -0.20 | 0.04 | 1.00 |  | -0.20 | 0.04 | 1.00 |  | -0.21 | 0.04 | 1.00 |  |  |  |  |
| Group |  |  |  |  |  |  |  |  | 5.53 | 1.04 | 1.00 |  | 5.53 | 1.04 | 1.00 |  | 5.52 | 1.07 | 1.00 |
| P-I x Group |  |  |  |  |  |  |  |  |  |  |  |  | 0.30 | 0.07 | 0.98 |  | 0.28 | 0.07 | 0.96 |
| P-II x Group |  |  |  |  |  |  |  |  |  |  |  |  | -0.00 | 0.07 | 0.06 |  | -0.00 | 0.08 | 0.06 |
| **Relative change score** |  |  |  |  |  |  |  |  |  |  |  |  |  |  |  |  |  |  |  |
| Intercept | 7.75 | 1.20 | 1.00 |  | 7.75 | 1.20 | 1.00 |  | 2.46 | 1.46 | 0.57 |  | 2.46 | 1.45 | 0.57 |  | 2.47 | 1.49 | 0.55 |
| P-I | 0.28 | 0.09 | 0.89 |  | 0.31 | 0.08 | 0.97 |  | 0.31 | 0.08 | 0.98 |  | 0.00 | 0.10 | 0.02 |  | 0.00 | 0.11 | 0.02 |
| P-II | -0.00 | 0.09 | 0.06 |  | -0.00 | 0.08 | 0.05 |  | -0.01 | 0.08 | 0.05 |  | -0.00 | 0.10 | 0.03 |  | 0.00 | 0.11 | 0.03 |
| Pre-test score |  |  |  |  | -0.61 | 0.11 | 1.00 |  | -0.61 | 0.11 | 1.00 |  | -0.63 | 0.10 | 1.00 |  |  |  |  |
| Group |  |  |  |  |  |  |  |  | 10.59 | 2.23 | 1.00 |  | 10.59 | 2.23 | 1.00 |  | 10.56 | 2.33 | 1.00 |
| P-I x Group |  |  |  |  |  |  |  |  |  |  |  |  | 0.61 | 0.15 | 0.97 |  | 0.55 | 0.16 | 0.92 |
| P-II x Group |  |  |  |  |  |  |  |  |  |  |  |  | -0.01 | 0.16 | 0.05 |  | -0.01 | 0.17 | 0.05 |
| **Residual score** |  |  |  |  |  |  |  |  |  |  |  |  |  |  |  |  |  |  |  |
| Intercept | -0.00 | 0.00 | 0.00 |  | -0.00 | 0.00 | 0.00 |  | -2.76 | 0.52 | 1.00 |  | -2.76 | 0.52 | 1.00 |  | -2.76 | 0.52 | 1.00 |
| P-I | 0.15 | 0.04 | 0.97 |  | 0.15 | 0.04 | 0.97 |  | 0.15 | 0.04 | 0.98 |  | 0.00 | 0.05 | 0.02 |  | 0.00 | 0.05 | 0.02 |
| P-II | -0.00 | 0.04 | 0.06 |  | -0.00 | 0.04 | 0.06 |  | -0.00 | 0.04 | 0.05 |  | -0.00 | 0.05 | 0.02 |  | -0.00 | 0.05 | 0.02 |
| Pre-test score |  |  |  |  | -0.01 | 0.01 | 0.00 |  | -0.01 | 0.01 | 0.00 |  | -0.01 | 0.02 | 0.00 |  |  |  |  |
| Group |  |  |  |  |  |  |  |  | 5.53 | 1.04 | 1.00 |  | 5.53 | 1.04 | 1.00 |  | 5.52 | 1.04 | 1.00 |
| P-I x Group |  |  |  |  |  |  |  |  |  |  |  |  | 0.30 | 0.07 | 0.98 |  | 0.30 | 0.07 | 0.98 |
| P-II x Group |  |  |  |  |  |  |  |  |  |  |  |  | -0.00 | 0.07 | 0.06 |  | -0.00 | 0.07 | 0.06 |

Table 25: Results of all investigated simulations ordered according to their used model and dependent variable, for a **reliability of .90**, and a sample size of ***n* = 50**

| **Coefficient** | **Model 1** | | |  | **Model 2** | | |  | **Model 3** | | |  | **Model 4** | | |  | **Model 5** | | |
| --- | --- | --- | --- | --- | --- | --- | --- | --- | --- | --- | --- | --- | --- | --- | --- | --- | --- | --- | --- |
|  | ***M*** | ***SE*** | ***P*** |  | ***M*** | ***SE*** | ***P*** |  | ***M*** | ***SE*** | ***P*** |  | ***M*** | ***SE*** | ***P*** |  | ***M*** | ***SE*** | ***P*** |
| **Post-test score** |  |  |  |  |  |  |  |  |  |  |  |  |  |  |  |  |  |  |  |
| Intercept | 53.64 | 2.17 | 1.00 |  | 53.64 | 2.17 | 1.00 |  | 50.56 | 2.48 | 1.00 |  | 50.55 | 2.47 | 1.00 |  | 50.58 | 2.76 | 1.00 |
| P-I | 0.20 | 0.18 | 0.21 |  | 0.17 | 0.11 | 0.26 |  | 0.17 | 0.10 | 0.32 |  | 0.00 | 0.14 | 0.03 |  | -0.01 | 0.22 | 0.03 |
| P-II | 0.00 | 0.17 | 0.05 |  | -0.00 | 0.12 | 0.05 |  | -0.01 | 0.11 | 0.06 |  | -0.00 | 0.14 | 0.04 |  | -0.00 | 0.21 | 0.02 |
| Pre-test score |  |  |  |  | 0.90 | 0.15 | 1.00 |  | 0.90 | 0.14 | 1.00 |  | 0.89 | 0.13 | 1.00 |  |  |  |  |
| Group |  |  |  |  |  |  |  |  | 6.16 | 3.38 | 0.71 |  | 6.15 | 3.39 | 0.72 |  | 6.09 | 4.29 | 0.46 |
| P-I x Group |  |  |  |  |  |  |  |  |  |  |  |  | 0.34 | 0.21 | 0.32 |  | 0.43 | 0.36 | 0.24 |
| P-II x Group |  |  |  |  |  |  |  |  |  |  |  |  | -0.01 | 0.22 | 0.05 |  | -0.01 | 0.35 | 0.06 |
| **Absolute change score** |  |  |  |  |  |  |  |  |  |  |  |  |  |  |  |  |  |  |  |
| Intercept | 3.57 | 1.71 | 0.78 |  | 3.57 | 1.71 | 0.79 |  | 0.49 | 2.10 | 0.19 |  | 0.49 | 2.11 | 0.19 |  | 0.48 | 2.11 | 0.18 |
| P-I | 0.17 | 0.11 | 0.26 |  | 0.17 | 0.11 | 0.26 |  | 0.17 | 0.10 | 0.32 |  | 0.00 | 0.14 | 0.03 |  | 0.00 | 0.15 | 0.04 |
| P-II | -0.00 | 0.12 | 0.04 |  | -0.00 | 0.12 | 0.05 |  | -0.01 | 0.11 | 0.06 |  | -0.00 | 0.14 | 0.04 |  | -0.00 | 0.15 | 0.04 |
| Pre-test score |  |  |  |  | -0.10 | 0.15 | 0.21 |  | -0.10 | 0.14 | 0.24 |  | -0.11 | 0.13 | 0.25 |  |  |  |  |
| Group |  |  |  |  |  |  |  |  | 6.16 | 3.38 | 0.71 |  | 6.15 | 3.39 | 0.72 |  | 6.16 | 3.37 | 0.71 |
| P-I x Group |  |  |  |  |  |  |  |  |  |  |  |  | 0.34 | 0.21 | 0.32 |  | 0.34 | 0.21 | 0.31 |
| P-II x Group |  |  |  |  |  |  |  |  |  |  |  |  | -0.01 | 0.22 | 0.05 |  | -0.01 | 0.22 | 0.04 |
| **Relative change score** |  |  |  |  |  |  |  |  |  |  |  |  |  |  |  |  |  |  |  |
| Intercept | 7.96 | 3.73 | 0.80 |  | 7.96 | 3.73 | 0.81 |  | 2.02 | 4.56 | 0.21 |  | 2.01 | 4.59 | 0.22 |  | 1.99 | 4.60 | 0.19 |
| P-I | 0.34 | 0.25 | 0.23 |  | 0.34 | 0.24 | 0.26 |  | 0.35 | 0.22 | 0.29 |  | 0.00 | 0.31 | 0.04 |  | 0.01 | 0.33 | 0.04 |
| P-II | -0.00 | 0.26 | 0.05 |  | -0.00 | 0.25 | 0.06 |  | -0.01 | 0.24 | 0.05 |  | -0.00 | 0.31 | 0.04 |  | -0.00 | 0.32 | 0.03 |
| Pre-test score |  |  |  |  | -0.39 | 0.37 | 0.40 |  | -0.39 | 0.36 | 0.43 |  | -0.41 | 0.35 | 0.44 |  |  |  |  |
| Group |  |  |  |  |  |  |  |  | 11.88 | 7.15 | 0.66 |  | 11.85 | 7.17 | 0.66 |  | 11.91 | 7.21 | 0.64 |
| P-I x Group |  |  |  |  |  |  |  |  |  |  |  |  | 0.71 | 0.45 | 0.31 |  | 0.68 | 0.47 | 0.28 |
| P-II x Group |  |  |  |  |  |  |  |  |  |  |  |  | -0.02 | 0.48 | 0.05 |  | -0.02 | 0.49 | 0.05 |
| **Residual score** |  |  |  |  |  |  |  |  |  |  |  |  |  |  |  |  |  |  |  |
| Intercept | -0.00 | 0.00 | 0.00 |  | -0.00 | 0.00 | 0.00 |  | -3.08 | 1.69 | 0.54 |  | -3.08 | 1.70 | 0.52 |  | -3.03 | 1.67 | 0.51 |
| P-I | 0.16 | 0.11 | 0.26 |  | 0.17 | 0.11 | 0.26 |  | 0.17 | 0.10 | 0.32 |  | 0.00 | 0.14 | 0.03 |  | 0.00 | 0.14 | 0.04 |
| P-II | -0.00 | 0.12 | 0.05 |  | -0.00 | 0.12 | 0.05 |  | -0.01 | 0.11 | 0.06 |  | -0.00 | 0.14 | 0.04 |  | -0.00 | 0.14 | 0.04 |
| Pre-test score |  |  |  |  | -0.01 | 0.03 | 0.00 |  | -0.01 | 0.06 | 0.00 |  | -0.02 | 0.07 | 0.00 |  |  |  |  |
| Group |  |  |  |  |  |  |  |  | 6.16 | 3.38 | 0.71 |  | 6.15 | 3.39 | 0.72 |  | 6.04 | 3.34 | 0.71 |
| P-I x Group |  |  |  |  |  |  |  |  |  |  |  |  | 0.34 | 0.21 | 0.32 |  | 0.33 | 0.21 | 0.31 |
| P-II x Group |  |  |  |  |  |  |  |  |  |  |  |  | -0.01 | 0.22 | 0.05 |  | -0.01 | 0.22 | 0.05 |

Table 26: Results of all investigated simulations ordered according to their used model and dependent variable, for a **reliability of .90**, and a sample size of ***n* = 100**

| **Coefficient** | **Model 1** | | |  | **Model 2** | | |  | **Model 3** | | |  | **Model 4** | | |  | **Model 5** | | |
| --- | --- | --- | --- | --- | --- | --- | --- | --- | --- | --- | --- | --- | --- | --- | --- | --- | --- | --- | --- |
|  | ***M*** | ***SE*** | ***P*** |  | ***M*** | ***SE*** | ***P*** |  | ***M*** | ***SE*** | ***P*** |  | ***M*** | ***SE*** | ***P*** |  | ***M*** | ***SE*** | ***P*** |
| **Post-test score** |  |  |  |  |  |  |  |  |  |  |  |  |  |  |  |  |  |  |  |
| Intercept | 53.51 | 1.55 | 1.00 |  | 53.51 | 1.55 | 1.00 |  | 50.54 | 1.80 | 1.00 |  | 50.53 | 1.81 | 1.00 |  | 50.54 | 2.05 | 1.00 |
| P-I | 0.20 | 0.13 | 0.36 |  | 0.16 | 0.08 | 0.52 |  | 0.16 | 0.07 | 0.61 |  | -0.00 | 0.09 | 0.03 |  | -0.00 | 0.15 | 0.03 |
| P-II | -0.00 | 0.12 | 0.05 |  | -0.01 | 0.08 | 0.05 |  | -0.00 | 0.07 | 0.04 |  | -0.01 | 0.09 | 0.04 |  | -0.00 | 0.15 | 0.03 |
| Pre-test score |  |  |  |  | 0.89 | 0.10 | 1.00 |  | 0.89 | 0.10 | 1.00 |  | 0.88 | 0.09 | 1.00 |  |  |  |  |
| Group |  |  |  |  |  |  |  |  | 5.95 | 2.38 | 0.89 |  | 5.95 | 2.38 | 0.90 |  | 5.93 | 3.04 | 0.67 |
| P-I x Group |  |  |  |  |  |  |  |  |  |  |  |  | 0.33 | 0.14 | 0.64 |  | 0.41 | 0.24 | 0.41 |
| P-II x Group |  |  |  |  |  |  |  |  |  |  |  |  | 0.01 | 0.14 | 0.05 |  | 0.00 | 0.24 | 0.06 |
| **Absolute change score** |  |  |  |  |  |  |  |  |  |  |  |  |  |  |  |  |  |  |  |
| Intercept | 3.49 | 1.24 | 0.94 |  | 3.49 | 1.24 | 0.94 |  | 0.52 | 1.52 | 0.20 |  | 0.51 | 1.52 | 0.20 |  | 0.51 | 1.52 | 0.20 |
| P-I | 0.16 | 0.08 | 0.49 |  | 0.16 | 0.08 | 0.52 |  | 0.16 | 0.07 | 0.61 |  | -0.00 | 0.09 | 0.03 |  | -0.00 | 0.10 | 0.04 |
| P-II | -0.01 | 0.08 | 0.05 |  | -0.01 | 0.08 | 0.05 |  | -0.00 | 0.07 | 0.04 |  | -0.01 | 0.09 | 0.04 |  | -0.01 | 0.10 | 0.04 |
| Pre-test score |  |  |  |  | -0.11 | 0.10 | 0.33 |  | -0.11 | 0.10 | 0.38 |  | -0.12 | 0.09 | 0.41 |  |  |  |  |
| Group |  |  |  |  |  |  |  |  | 5.95 | 2.38 | 0.89 |  | 5.95 | 2.38 | 0.90 |  | 5.96 | 2.38 | 0.90 |
| P-I x Group |  |  |  |  |  |  |  |  |  |  |  |  | 0.33 | 0.14 | 0.64 |  | 0.32 | 0.14 | 0.61 |
| P-II x Group |  |  |  |  |  |  |  |  |  |  |  |  | 0.01 | 0.14 | 0.05 |  | 0.01 | 0.14 | 0.04 |
| **Relative change score** |  |  |  |  |  |  |  |  |  |  |  |  |  |  |  |  |  |  |  |
| Intercept | 7.80 | 2.74 | 0.95 |  | 7.80 | 2.74 | 0.96 |  | 2.09 | 3.52 | 0.25 |  | 2.08 | 3.51 | 0.26 |  | 2.07 | 3.50 | 0.24 |
| P-I | 0.31 | 0.17 | 0.43 |  | 0.33 | 0.16 | 0.48 |  | 0.33 | 0.15 | 0.55 |  | -0.00 | 0.20 | 0.04 |  | -0.00 | 0.23 | 0.04 |
| P-II | -0.01 | 0.18 | 0.04 |  | -0.01 | 0.17 | 0.05 |  | -0.01 | 0.15 | 0.04 |  | -0.01 | 0.22 | 0.03 |  | -0.01 | 0.25 | 0.04 |
| Pre-test score |  |  |  |  | -0.41 | 0.42 | 0.63 |  | -0.41 | 0.42 | 0.67 |  | -0.42 | 0.41 | 0.69 |  |  |  |  |
| Group |  |  |  |  |  |  |  |  | 11.42 | 5.24 | 0.84 |  | 11.42 | 5.24 | 0.85 |  | 11.44 | 5.29 | 0.84 |
| P-I x Group |  |  |  |  |  |  |  |  |  |  |  |  | 0.68 | 0.30 | 0.59 |  | 0.65 | 0.31 | 0.51 |
| P-II x Group |  |  |  |  |  |  |  |  |  |  |  |  | 0.01 | 0.34 | 0.05 |  | 0.01 | 0.35 | 0.04 |
| **Residual score** |  |  |  |  |  |  |  |  |  |  |  |  |  |  |  |  |  |  |  |
| Intercept | 0.00 | 0.00 | 0.00 |  | 0.00 | 0.00 | 0.00 |  | -2.97 | 1.19 | 0.79 |  | -2.98 | 1.19 | 0.80 |  | -2.95 | 1.18 | 0.80 |
| P-I | 0.16 | 0.07 | 0.52 |  | 0.16 | 0.08 | 0.52 |  | 0.16 | 0.07 | 0.61 |  | -0.00 | 0.09 | 0.03 |  | -0.00 | 0.09 | 0.03 |
| P-II | -0.01 | 0.08 | 0.05 |  | -0.01 | 0.08 | 0.05 |  | -0.00 | 0.07 | 0.04 |  | -0.01 | 0.09 | 0.04 |  | -0.01 | 0.09 | 0.04 |
| Pre-test score |  |  |  |  | -0.01 | 0.02 | 0.00 |  | -0.01 | 0.04 | 0.00 |  | -0.01 | 0.04 | 0.00 |  |  |  |  |
| Group |  |  |  |  |  |  |  |  | 5.95 | 2.38 | 0.89 |  | 5.95 | 2.38 | 0.90 |  | 5.89 | 2.36 | 0.90 |
| P-I x Group |  |  |  |  |  |  |  |  |  |  |  |  | 0.33 | 0.14 | 0.64 |  | 0.32 | 0.13 | 0.64 |
| P-II x Group |  |  |  |  |  |  |  |  |  |  |  |  | 0.01 | 0.14 | 0.05 |  | 0.01 | 0.14 | 0.05 |

Table 27: Results of all investigated simulations ordered according to their used model and dependent variable, for a **reliability of .90**, and a sample size of ***n* = 150**

| **Coefficient** | **Model 1** | | |  | **Model 2** | | |  | **Model 3** | | |  | **Model 4** | | |  | **Model 5** | | |
| --- | --- | --- | --- | --- | --- | --- | --- | --- | --- | --- | --- | --- | --- | --- | --- | --- | --- | --- | --- |
|  | ***M*** | ***SE*** | ***P*** |  | ***M*** | ***SE*** | ***P*** |  | ***M*** | ***SE*** | ***P*** |  | ***M*** | ***SE*** | ***P*** |  | ***M*** | ***SE*** | ***P*** |
| **Post-test score** |  |  |  |  |  |  |  |  |  |  |  |  |  |  |  |  |  |  |  |
| Intercept | 53.44 | 1.23 | 1.00 |  | 53.44 | 1.23 | 1.00 |  | 50.49 | 1.41 | 1.00 |  | 50.49 | 1.41 | 1.00 |  | 50.47 | 1.56 | 1.00 |
| P-I | 0.21 | 0.10 | 0.55 |  | 0.16 | 0.06 | 0.75 |  | 0.17 | 0.06 | 0.81 |  | 0.00 | 0.08 | 0.03 |  | 0.01 | 0.12 | 0.03 |
| P-II | -0.01 | 0.10 | 0.05 |  | -0.00 | 0.07 | 0.06 |  | -0.00 | 0.06 | 0.06 |  | 0.00 | 0.08 | 0.04 |  | -0.00 | 0.12 | 0.02 |
| Pre-test score |  |  |  |  | 0.89 | 0.08 | 1.00 |  | 0.89 | 0.08 | 1.00 |  | 0.89 | 0.07 | 1.00 |  |  |  |  |
| Group |  |  |  |  |  |  |  |  | 5.90 | 1.93 | 0.96 |  | 5.89 | 1.93 | 0.96 |  | 5.92 | 2.39 | 0.83 |
| P-I x Group |  |  |  |  |  |  |  |  |  |  |  |  | 0.33 | 0.11 | 0.83 |  | 0.40 | 0.20 | 0.57 |
| P-II x Group |  |  |  |  |  |  |  |  |  |  |  |  | -0.01 | 0.12 | 0.05 |  | -0.01 | 0.19 | 0.06 |
| **Absolute change score** |  |  |  |  |  |  |  |  |  |  |  |  |  |  |  |  |  |  |  |
| Intercept | 3.45 | 0.99 | 0.99 |  | 3.45 | 0.99 | 0.99 |  | 0.51 | 1.19 | 0.21 |  | 0.50 | 1.19 | 0.22 |  | 0.50 | 1.20 | 0.22 |
| P-I | 0.16 | 0.06 | 0.70 |  | 0.16 | 0.06 | 0.75 |  | 0.17 | 0.06 | 0.81 |  | 0.00 | 0.08 | 0.03 |  | 0.00 | 0.08 | 0.04 |
| P-II | -0.00 | 0.07 | 0.06 |  | -0.00 | 0.07 | 0.06 |  | -0.00 | 0.06 | 0.06 |  | 0.00 | 0.08 | 0.04 |  | 0.00 | 0.08 | 0.04 |
| Pre-test score |  |  |  |  | -0.11 | 0.08 | 0.42 |  | -0.11 | 0.08 | 0.46 |  | -0.11 | 0.07 | 0.51 |  |  |  |  |
| Group |  |  |  |  |  |  |  |  | 5.90 | 1.93 | 0.96 |  | 5.89 | 1.93 | 0.96 |  | 5.89 | 1.95 | 0.96 |
| P-I x Group |  |  |  |  |  |  |  |  |  |  |  |  | 0.33 | 0.11 | 0.83 |  | 0.32 | 0.11 | 0.79 |
| P-II x Group |  |  |  |  |  |  |  |  |  |  |  |  | -0.01 | 0.12 | 0.05 |  | -0.01 | 0.12 | 0.05 |
| **Relative change score** |  |  |  |  |  |  |  |  |  |  |  |  |  |  |  |  |  |  |  |
| Intercept | 7.71 | 2.13 | 1.00 |  | 7.71 | 2.13 | 1.00 |  | 2.07 | 2.57 | 0.29 |  | 2.05 | 2.57 | 0.30 |  | 2.06 | 2.59 | 0.28 |
| P-I | 0.32 | 0.13 | 0.65 |  | 0.34 | 0.13 | 0.72 |  | 0.34 | 0.12 | 0.79 |  | 0.01 | 0.17 | 0.02 |  | 0.01 | 0.18 | 0.04 |
| P-II | -0.01 | 0.14 | 0.06 |  | -0.01 | 0.14 | 0.06 |  | -0.00 | 0.13 | 0.06 |  | 0.00 | 0.17 | 0.04 |  | 0.00 | 0.18 | 0.04 |
| Pre-test score |  |  |  |  | -0.40 | 0.20 | 0.75 |  | -0.41 | 0.19 | 0.78 |  | -0.42 | 0.19 | 0.82 |  |  |  |  |
| Group |  |  |  |  |  |  |  |  | 11.30 | 4.09 | 0.93 |  | 11.29 | 4.09 | 0.93 |  | 11.28 | 4.16 | 0.92 |
| P-I x Group |  |  |  |  |  |  |  |  |  |  |  |  | 0.67 | 0.23 | 0.78 |  | 0.64 | 0.24 | 0.69 |
| P-II x Group |  |  |  |  |  |  |  |  |  |  |  |  | -0.01 | 0.26 | 0.06 |  | -0.01 | 0.27 | 0.06 |
| **Residual score** |  |  |  |  |  |  |  |  |  |  |  |  |  |  |  |  |  |  |  |
| Intercept | 0.00 | 0.00 | 0.00 |  | 0.00 | 0.00 | 0.00 |  | -2.95 | 0.96 | 0.91 |  | -2.95 | 0.95 | 0.92 |  | -2.93 | 0.95 | 0.92 |
| P-I | 0.16 | 0.06 | 0.74 |  | 0.16 | 0.06 | 0.75 |  | 0.17 | 0.06 | 0.81 |  | 0.00 | 0.08 | 0.03 |  | 0.00 | 0.08 | 0.03 |
| P-II | -0.00 | 0.07 | 0.06 |  | -0.00 | 0.07 | 0.06 |  | -0.00 | 0.06 | 0.06 |  | 0.00 | 0.08 | 0.04 |  | 0.00 | 0.08 | 0.03 |
| Pre-test score |  |  |  |  | -0.01 | 0.02 | 0.00 |  | -0.01 | 0.03 | 0.00 |  | -0.02 | 0.03 | 0.00 |  |  |  |  |
| Group |  |  |  |  |  |  |  |  | 5.90 | 1.93 | 0.96 |  | 5.89 | 1.93 | 0.96 |  | 5.85 | 1.92 | 0.96 |
| P-I x Group |  |  |  |  |  |  |  |  |  |  |  |  | 0.33 | 0.11 | 0.83 |  | 0.33 | 0.11 | 0.82 |
| P-II x Group |  |  |  |  |  |  |  |  |  |  |  |  | -0.01 | 0.12 | 0.05 |  | -0.01 | 0.12 | 0.05 |

Table 28: Results of all investigated simulations ordered according to their used model and dependent variable, for a **reliability of .90**, and a sample size of ***n* = 200**

| **Coefficient** | **Model 1** | | |  | **Model 2** | | |  | **Model 3** | | |  | **Model 4** | | |  | **Model 5** | | |
| --- | --- | --- | --- | --- | --- | --- | --- | --- | --- | --- | --- | --- | --- | --- | --- | --- | --- | --- | --- |
|  | ***M*** | ***SE*** | ***P*** |  | ***M*** | ***SE*** | ***P*** |  | ***M*** | ***SE*** | ***P*** |  | ***M*** | ***SE*** | ***P*** |  | ***M*** | ***SE*** | ***P*** |
| **Post-test score** |  |  |  |  |  |  |  |  |  |  |  |  |  |  |  |  |  |  |  |
| Intercept | 53.46 | 1.03 | 1.00 |  | 53.46 | 1.03 | 1.00 |  | 50.55 | 1.24 | 1.00 |  | 50.55 | 1.24 | 1.00 |  | 50.56 | 1.40 | 1.00 |
| P-I | 0.21 | 0.09 | 0.66 |  | 0.16 | 0.05 | 0.85 |  | 0.17 | 0.05 | 0.92 |  | 0.00 | 0.06 | 0.03 |  | -0.00 | 0.10 | 0.02 |
| P-II | -0.00 | 0.09 | 0.06 |  | -0.00 | 0.06 | 0.05 |  | -0.00 | 0.05 | 0.05 |  | 0.00 | 0.06 | 0.02 |  | -0.00 | 0.10 | 0.03 |
| Pre-test score |  |  |  |  | 0.90 | 0.07 | 1.00 |  | 0.90 | 0.06 | 1.00 |  | 0.89 | 0.06 | 1.00 |  |  |  |  |
| Group |  |  |  |  |  |  |  |  | 5.82 | 1.79 | 0.98 |  | 5.83 | 1.79 | 0.98 |  | 5.81 | 2.21 | 0.88 |
| P-I x Group |  |  |  |  |  |  |  |  |  |  |  |  | 0.33 | 0.10 | 0.94 |  | 0.42 | 0.17 | 0.73 |
| P-II x Group |  |  |  |  |  |  |  |  |  |  |  |  | -0.00 | 0.10 | 0.05 |  | -0.00 | 0.17 | 0.06 |
| **Absolute change score** |  |  |  |  |  |  |  |  |  |  |  |  |  |  |  |  |  |  |  |
| Intercept | 3.48 | 0.85 | 1.00 |  | 3.48 | 0.85 | 1.00 |  | 0.57 | 1.07 | 0.26 |  | 0.57 | 1.07 | 0.27 |  | 0.57 | 1.07 | 0.26 |
| P-I | 0.16 | 0.05 | 0.83 |  | 0.16 | 0.05 | 0.85 |  | 0.17 | 0.05 | 0.92 |  | 0.00 | 0.06 | 0.03 |  | 0.00 | 0.07 | 0.04 |
| P-II | -0.00 | 0.06 | 0.04 |  | -0.00 | 0.06 | 0.05 |  | -0.00 | 0.05 | 0.05 |  | 0.00 | 0.06 | 0.02 |  | 0.00 | 0.07 | 0.03 |
| Pre-test score |  |  |  |  | -0.10 | 0.07 | 0.49 |  | -0.10 | 0.06 | 0.54 |  | -0.11 | 0.06 | 0.59 |  |  |  |  |
| Group |  |  |  |  |  |  |  |  | 5.82 | 1.79 | 0.98 |  | 5.83 | 1.79 | 0.98 |  | 5.83 | 1.79 | 0.99 |
| P-I x Group |  |  |  |  |  |  |  |  |  |  |  |  | 0.33 | 0.10 | 0.94 |  | 0.32 | 0.10 | 0.92 |
| P-II x Group |  |  |  |  |  |  |  |  |  |  |  |  | -0.00 | 0.10 | 0.05 |  | -0.00 | 0.10 | 0.05 |
| **Relative change score** |  |  |  |  |  |  |  |  |  |  |  |  |  |  |  |  |  |  |  |
| Intercept | 7.77 | 1.82 | 1.00 |  | 7.77 | 1.82 | 1.00 |  | 2.23 | 2.30 | 0.38 |  | 2.23 | 2.29 | 0.40 |  | 2.22 | 2.29 | 0.37 |
| P-I | 0.32 | 0.11 | 0.78 |  | 0.34 | 0.11 | 0.83 |  | 0.34 | 0.10 | 0.90 |  | 0.00 | 0.14 | 0.03 |  | 0.00 | 0.15 | 0.04 |
| P-II | -0.00 | 0.12 | 0.04 |  | -0.00 | 0.12 | 0.05 |  | -0.00 | 0.11 | 0.05 |  | 0.00 | 0.13 | 0.02 |  | 0.00 | 0.14 | 0.03 |
| Pre-test score |  |  |  |  | -0.40 | 0.17 | 0.84 |  | -0.40 | 0.16 | 0.87 |  | -0.42 | 0.16 | 0.90 |  |  |  |  |
| Group |  |  |  |  |  |  |  |  | 11.08 | 3.82 | 0.96 |  | 11.08 | 3.81 | 0.96 |  | 11.10 | 3.87 | 0.96 |
| P-I x Group |  |  |  |  |  |  |  |  |  |  |  |  | 0.68 | 0.21 | 0.91 |  | 0.65 | 0.21 | 0.85 |
| P-II x Group |  |  |  |  |  |  |  |  |  |  |  |  | -0.01 | 0.22 | 0.06 |  | -0.01 | 0.23 | 0.05 |
| **Residual score** |  |  |  |  |  |  |  |  |  |  |  |  |  |  |  |  |  |  |  |
| Intercept | -0.00 | 0.00 | 0.00 |  | 0.00 | 0.00 | 0.00 |  | -2.91 | 0.90 | 0.95 |  | -2.91 | 0.89 | 0.95 |  | -2.90 | 0.89 | 0.95 |
| P-I | 0.16 | 0.05 | 0.85 |  | 0.16 | 0.05 | 0.85 |  | 0.17 | 0.05 | 0.92 |  | 0.00 | 0.06 | 0.03 |  | 0.00 | 0.06 | 0.03 |
| P-II | -0.00 | 0.06 | 0.05 |  | -0.00 | 0.06 | 0.05 |  | -0.00 | 0.05 | 0.05 |  | 0.00 | 0.06 | 0.02 |  | 0.00 | 0.06 | 0.02 |
| Pre-test score |  |  |  |  | -0.01 | 0.01 | 0.00 |  | -0.01 | 0.03 | 0.00 |  | -0.02 | 0.03 | 0.00 |  |  |  |  |
| Group |  |  |  |  |  |  |  |  | 5.82 | 1.79 | 0.98 |  | 5.83 | 1.79 | 0.98 |  | 5.79 | 1.78 | 0.99 |
| P-I x Group |  |  |  |  |  |  |  |  |  |  |  |  | 0.33 | 0.10 | 0.94 |  | 0.33 | 0.10 | 0.94 |
| P-II x Group |  |  |  |  |  |  |  |  |  |  |  |  | -0.00 | 0.10 | 0.05 |  | -0.00 | 0.10 | 0.05 |

Table 29: Results of all investigated simulations ordered according to their used model and dependent variable, for a **reliability of .90**, and a sample size of ***n* = 250**

| **Coefficient** | **Model 1** | | |  | **Model 2** | | |  | **Model 3** | | |  | **Model 4** | | |  | **Model 5** | | |
| --- | --- | --- | --- | --- | --- | --- | --- | --- | --- | --- | --- | --- | --- | --- | --- | --- | --- | --- | --- |
|  | ***M*** | ***SE*** | ***P*** |  | ***M*** | ***SE*** | ***P*** |  | ***M*** | ***SE*** | ***P*** |  | ***M*** | ***SE*** | ***P*** |  | ***M*** | ***SE*** | ***P*** |
| **Post-test score** |  |  |  |  |  |  |  |  |  |  |  |  |  |  |  |  |  |  |  |
| Intercept | 53.45 | 0.92 | 1.00 |  | 53.45 | 0.92 | 1.00 |  | 50.55 | 1.07 | 1.00 |  | 50.54 | 1.07 | 1.00 |  | 50.56 | 1.22 | 1.00 |
| P-I | 0.21 | 0.08 | 0.78 |  | 0.16 | 0.05 | 0.91 |  | 0.16 | 0.04 | 0.96 |  | -0.00 | 0.06 | 0.04 |  | -0.00 | 0.10 | 0.04 |
| P-II | -0.00 | 0.08 | 0.05 |  | 0.00 | 0.05 | 0.06 |  | 0.00 | 0.05 | 0.06 |  | 0.00 | 0.06 | 0.03 |  | 0.00 | 0.09 | 0.03 |
| Pre-test score |  |  |  |  | 0.90 | 0.06 | 1.00 |  | 0.90 | 0.06 | 1.00 |  | 0.89 | 0.06 | 1.00 |  |  |  |  |
| Group |  |  |  |  |  |  |  |  | 5.81 | 1.55 | 0.99 |  | 5.81 | 1.55 | 0.99 |  | 5.77 | 1.92 | 0.95 |
| P-I x Group |  |  |  |  |  |  |  |  |  |  |  |  | 0.33 | 0.08 | 0.97 |  | 0.41 | 0.15 | 0.80 |
| P-II x Group |  |  |  |  |  |  |  |  |  |  |  |  | -0.00 | 0.09 | 0.04 |  | -0.00 | 0.14 | 0.05 |
| **Absolute change score** |  |  |  |  |  |  |  |  |  |  |  |  |  |  |  |  |  |  |  |
| Intercept | 3.43 | 0.76 | 1.00 |  | 3.43 | 0.76 | 1.00 |  | 0.53 | 0.95 | 0.24 |  | 0.52 | 0.95 | 0.26 |  | 0.52 | 0.95 | 0.26 |
| P-I | 0.16 | 0.05 | 0.90 |  | 0.16 | 0.05 | 0.91 |  | 0.16 | 0.04 | 0.96 |  | -0.00 | 0.06 | 0.04 |  | -0.00 | 0.06 | 0.04 |
| P-II | 0.00 | 0.05 | 0.05 |  | 0.00 | 0.05 | 0.06 |  | 0.00 | 0.05 | 0.06 |  | 0.00 | 0.06 | 0.03 |  | 0.00 | 0.06 | 0.04 |
| Pre-test score |  |  |  |  | -0.10 | 0.06 | 0.56 |  | -0.10 | 0.06 | 0.59 |  | -0.11 | 0.06 | 0.66 |  |  |  |  |
| Group |  |  |  |  |  |  |  |  | 5.81 | 1.55 | 0.99 |  | 5.81 | 1.55 | 0.99 |  | 5.82 | 1.55 | 1.00 |
| P-I x Group |  |  |  |  |  |  |  |  |  |  |  |  | 0.33 | 0.08 | 0.97 |  | 0.32 | 0.08 | 0.95 |
| P-II x Group |  |  |  |  |  |  |  |  |  |  |  |  | -0.00 | 0.09 | 0.04 |  | -0.00 | 0.09 | 0.04 |
| **Relative change score** |  |  |  |  |  |  |  |  |  |  |  |  |  |  |  |  |  |  |  |
| Intercept | 7.65 | 1.63 | 1.00 |  | 7.65 | 1.63 | 1.00 |  | 2.11 | 2.08 | 0.41 |  | 2.10 | 2.08 | 0.41 |  | 2.08 | 2.08 | 0.39 |
| P-I | 0.31 | 0.11 | 0.83 |  | 0.33 | 0.10 | 0.89 |  | 0.33 | 0.09 | 0.94 |  | -0.00 | 0.13 | 0.04 |  | -0.00 | 0.14 | 0.04 |
| P-II | 0.00 | 0.11 | 0.04 |  | 0.00 | 0.10 | 0.05 |  | 0.00 | 0.10 | 0.05 |  | 0.00 | 0.13 | 0.04 |  | 0.00 | 0.13 | 0.04 |
| Pre-test score |  |  |  |  | -0.40 | 0.16 | 0.89 |  | -0.40 | 0.15 | 0.91 |  | -0.42 | 0.15 | 0.94 |  |  |  |  |
| Group |  |  |  |  |  |  |  |  | 11.08 | 3.33 | 0.99 |  | 11.08 | 3.33 | 0.99 |  | 11.10 | 3.36 | 0.99 |
| P-I x Group |  |  |  |  |  |  |  |  |  |  |  |  | 0.67 | 0.18 | 0.95 |  | 0.63 | 0.19 | 0.89 |
| P-II x Group |  |  |  |  |  |  |  |  |  |  |  |  | -0.00 | 0.19 | 0.04 |  | -0.00 | 0.20 | 0.04 |
| **Residual score** |  |  |  |  |  |  |  |  |  |  |  |  |  |  |  |  |  |  |  |
| Intercept | -0.00 | 0.00 | 0.00 |  | -0.00 | 0.00 | 0.00 |  | -2.90 | 0.78 | 0.99 |  | -2.91 | 0.77 | 0.99 |  | -2.90 | 0.77 | 0.99 |
| P-I | 0.16 | 0.05 | 0.91 |  | 0.16 | 0.05 | 0.91 |  | 0.16 | 0.04 | 0.96 |  | -0.00 | 0.06 | 0.04 |  | -0.00 | 0.06 | 0.04 |
| P-II | 0.00 | 0.05 | 0.05 |  | 0.00 | 0.05 | 0.06 |  | 0.00 | 0.05 | 0.06 |  | 0.00 | 0.06 | 0.03 |  | 0.00 | 0.06 | 0.03 |
| Pre-test score |  |  |  |  | -0.01 | 0.01 | 0.00 |  | -0.01 | 0.02 | 0.00 |  | -0.02 | 0.03 | 0.00 |  |  |  |  |
| Group |  |  |  |  |  |  |  |  | 5.81 | 1.55 | 0.99 |  | 5.81 | 1.55 | 0.99 |  | 5.79 | 1.54 | 1.00 |
| P-I x Group |  |  |  |  |  |  |  |  |  |  |  |  | 0.33 | 0.08 | 0.97 |  | 0.32 | 0.08 | 0.97 |
| P-II x Group |  |  |  |  |  |  |  |  |  |  |  |  | -0.00 | 0.09 | 0.04 |  | -0.00 | 0.09 | 0.04 |

Table 30: Results of all investigated simulations ordered according to their used model and dependent variable, for a **reliability of .90**, and a sample size of ***n* = 300**

| **Coefficient** | **Model 1** | | |  | **Model 2** | | |  | **Model 3** | | |  | **Model 4** | | |  | **Model 5** | | |
| --- | --- | --- | --- | --- | --- | --- | --- | --- | --- | --- | --- | --- | --- | --- | --- | --- | --- | --- | --- |
|  | ***M*** | ***SE*** | ***P*** |  | ***M*** | ***SE*** | ***P*** |  | ***M*** | ***SE*** | ***P*** |  | ***M*** | ***SE*** | ***P*** |  | ***M*** | ***SE*** | ***P*** |
| **Post-test score** |  |  |  |  |  |  |  |  |  |  |  |  |  |  |  |  |  |  |  |
| Intercept | 53.49 | 0.91 | 1.00 |  | 53.49 | 0.91 | 1.00 |  | 50.53 | 1.02 | 1.00 |  | 50.54 | 1.02 | 1.00 |  | 50.54 | 1.16 | 1.00 |
| P-I | 0.21 | 0.07 | 0.85 |  | 0.16 | 0.04 | 0.96 |  | 0.16 | 0.04 | 0.99 |  | 0.00 | 0.05 | 0.03 |  | -0.00 | 0.08 | 0.02 |
| P-II | -0.00 | 0.07 | 0.05 |  | 0.00 | 0.05 | 0.05 |  | 0.00 | 0.04 | 0.05 |  | 0.00 | 0.05 | 0.02 |  | -0.00 | 0.08 | 0.03 |
| Pre-test score |  |  |  |  | 0.90 | 0.05 | 1.00 |  | 0.90 | 0.05 | 1.00 |  | 0.89 | 0.05 | 1.00 |  |  |  |  |
| Group |  |  |  |  |  |  |  |  | 5.92 | 1.43 | 1.00 |  | 5.92 | 1.43 | 1.00 |  | 5.90 | 1.79 | 0.97 |
| P-I x Group |  |  |  |  |  |  |  |  |  |  |  |  | 0.33 | 0.08 | 0.98 |  | 0.42 | 0.14 | 0.88 |
| P-II x Group |  |  |  |  |  |  |  |  |  |  |  |  | -0.00 | 0.08 | 0.06 |  | -0.00 | 0.13 | 0.05 |
| **Absolute change score** |  |  |  |  |  |  |  |  |  |  |  |  |  |  |  |  |  |  |  |
| Intercept | 3.45 | 0.71 | 1.00 |  | 3.45 | 0.71 | 1.00 |  | 0.49 | 0.88 | 0.27 |  | 0.50 | 0.88 | 0.27 |  | 0.50 | 0.88 | 0.28 |
| P-I | 0.16 | 0.04 | 0.95 |  | 0.16 | 0.04 | 0.96 |  | 0.16 | 0.04 | 0.99 |  | 0.00 | 0.05 | 0.03 |  | 0.00 | 0.05 | 0.04 |
| P-II | 0.00 | 0.05 | 0.05 |  | 0.00 | 0.05 | 0.05 |  | 0.00 | 0.04 | 0.05 |  | 0.00 | 0.05 | 0.02 |  | 0.00 | 0.05 | 0.03 |
| Pre-test score |  |  |  |  | -0.10 | 0.05 | 0.62 |  | -0.10 | 0.05 | 0.66 |  | -0.11 | 0.05 | 0.74 |  |  |  |  |
| Group |  |  |  |  |  |  |  |  | 5.92 | 1.43 | 1.00 |  | 5.92 | 1.43 | 1.00 |  | 5.92 | 1.43 | 1.00 |
| P-I x Group |  |  |  |  |  |  |  |  |  |  |  |  | 0.33 | 0.08 | 0.98 |  | 0.32 | 0.08 | 0.98 |
| P-II x Group |  |  |  |  |  |  |  |  |  |  |  |  | -0.00 | 0.08 | 0.06 |  | -0.00 | 0.08 | 0.05 |
| **Relative change score** |  |  |  |  |  |  |  |  |  |  |  |  |  |  |  |  |  |  |  |
| Intercept | 7.67 | 1.52 | 1.00 |  | 7.67 | 1.52 | 1.00 |  | 2.03 | 1.90 | 0.42 |  | 2.04 | 1.90 | 0.44 |  | 2.04 | 1.91 | 0.41 |
| P-I | 0.32 | 0.09 | 0.92 |  | 0.33 | 0.09 | 0.94 |  | 0.34 | 0.09 | 0.97 |  | 0.00 | 0.11 | 0.03 |  | 0.00 | 0.12 | 0.04 |
| P-II | 0.00 | 0.10 | 0.06 |  | 0.00 | 0.10 | 0.06 |  | 0.00 | 0.09 | 0.06 |  | 0.00 | 0.11 | 0.03 |  | 0.00 | 0.12 | 0.03 |
| Pre-test score |  |  |  |  | -0.40 | 0.14 | 0.93 |  | -0.40 | 0.13 | 0.94 |  | -0.41 | 0.13 | 0.97 |  |  |  |  |
| Group |  |  |  |  |  |  |  |  | 11.27 | 3.03 | 1.00 |  | 11.27 | 3.04 | 1.00 |  | 11.27 | 3.07 | 0.99 |
| P-I x Group |  |  |  |  |  |  |  |  |  |  |  |  | 0.67 | 0.16 | 0.99 |  | 0.63 | 0.17 | 0.96 |
| P-II x Group |  |  |  |  |  |  |  |  |  |  |  |  | 0.00 | 0.18 | 0.05 |  | 0.00 | 0.18 | 0.05 |
| **Residual score** |  |  |  |  |  |  |  |  |  |  |  |  |  |  |  |  |  |  |  |
| Intercept | 0.00 | 0.00 | 0.00 |  | 0.00 | 0.00 | 0.00 |  | -2.96 | 0.71 | 1.00 |  | -2.96 | 0.71 | 1.00 |  | -2.95 | 0.71 | 1.00 |
| P-I | 0.16 | 0.04 | 0.95 |  | 0.16 | 0.04 | 0.96 |  | 0.16 | 0.04 | 0.99 |  | 0.00 | 0.05 | 0.03 |  | 0.00 | 0.05 | 0.03 |
| P-II | 0.00 | 0.04 | 0.05 |  | 0.00 | 0.05 | 0.05 |  | 0.00 | 0.04 | 0.05 |  | 0.00 | 0.05 | 0.02 |  | 0.00 | 0.05 | 0.03 |
| Pre-test score |  |  |  |  | -0.01 | 0.01 | 0.00 |  | -0.01 | 0.02 | 0.00 |  | -0.02 | 0.02 | 0.01 |  |  |  |  |
| Group |  |  |  |  |  |  |  |  | 5.92 | 1.43 | 1.00 |  | 5.92 | 1.43 | 1.00 |  | 5.90 | 1.42 | 1.00 |
| P-I x Group |  |  |  |  |  |  |  |  |  |  |  |  | 0.33 | 0.08 | 0.98 |  | 0.33 | 0.08 | 0.99 |
| P-II x Group |  |  |  |  |  |  |  |  |  |  |  |  | -0.00 | 0.08 | 0.06 |  | -0.00 | 0.08 | 0.05 |

Table 31: Results of all investigated simulations ordered according to their used model and dependent variable, for a **reliability of .90**, and a sample size of ***n* = 400**

| **Coefficient** | **Model 1** | | |  | **Model 2** | | |  | **Model 3** | | |  | **Model 4** | | |  | **Model 5** | | |
| --- | --- | --- | --- | --- | --- | --- | --- | --- | --- | --- | --- | --- | --- | --- | --- | --- | --- | --- | --- |
|  | ***M*** | ***SE*** | ***P*** |  | ***M*** | ***SE*** | ***P*** |  | ***M*** | ***SE*** | ***P*** |  | ***M*** | ***SE*** | ***P*** |  | ***M*** | ***SE*** | ***P*** |
| **Post-test score** |  |  |  |  |  |  |  |  |  |  |  |  |  |  |  |  |  |  |  |
| Intercept | 53.49 | 0.75 | 1.00 |  | 53.49 | 0.75 | 1.00 |  | 50.54 | 0.87 | 1.00 |  | 50.53 | 0.86 | 1.00 |  | 50.54 | 0.98 | 1.00 |
| P-I | 0.21 | 0.06 | 0.92 |  | 0.16 | 0.04 | 0.99 |  | 0.16 | 0.03 | 1.00 |  | 0.00 | 0.04 | 0.03 |  | -0.00 | 0.07 | 0.03 |
| P-II | 0.00 | 0.06 | 0.07 |  | 0.00 | 0.04 | 0.04 |  | 0.00 | 0.04 | 0.05 |  | 0.00 | 0.05 | 0.05 |  | 0.00 | 0.07 | 0.04 |
| Pre-test score |  |  |  |  | 0.90 | 0.05 | 1.00 |  | 0.90 | 0.04 | 1.00 |  | 0.89 | 0.04 | 1.00 |  |  |  |  |
| Group |  |  |  |  |  |  |  |  | 5.91 | 1.22 | 1.00 |  | 5.91 | 1.22 | 1.00 |  | 5.90 | 1.54 | 0.99 |
| P-I x Group |  |  |  |  |  |  |  |  |  |  |  |  | 0.33 | 0.07 | 1.00 |  | 0.42 | 0.12 | 0.94 |
| P-II x Group |  |  |  |  |  |  |  |  |  |  |  |  | 0.00 | 0.07 | 0.05 |  | -0.00 | 0.11 | 0.05 |
| **Absolute change score** |  |  |  |  |  |  |  |  |  |  |  |  |  |  |  |  |  |  |  |
| Intercept | 3.49 | 0.61 | 1.00 |  | 3.49 | 0.61 | 1.00 |  | 0.53 | 0.75 | 0.29 |  | 0.53 | 0.74 | 0.31 |  | 0.53 | 0.74 | 0.30 |
| P-I | 0.16 | 0.04 | 0.99 |  | 0.16 | 0.04 | 0.99 |  | 0.16 | 0.03 | 1.00 |  | 0.00 | 0.04 | 0.03 |  | 0.00 | 0.05 | 0.04 |
| P-II | 0.00 | 0.04 | 0.05 |  | 0.00 | 0.04 | 0.04 |  | 0.00 | 0.04 | 0.05 |  | 0.00 | 0.05 | 0.05 |  | 0.00 | 0.05 | 0.05 |
| Pre-test score |  |  |  |  | -0.10 | 0.05 | 0.70 |  | -0.10 | 0.04 | 0.75 |  | -0.11 | 0.04 | 0.83 |  |  |  |  |
| Group |  |  |  |  |  |  |  |  | 5.91 | 1.22 | 1.00 |  | 5.91 | 1.22 | 1.00 |  | 5.92 | 1.23 | 1.00 |
| P-I x Group |  |  |  |  |  |  |  |  |  |  |  |  | 0.33 | 0.07 | 1.00 |  | 0.32 | 0.07 | 0.99 |
| P-II x Group |  |  |  |  |  |  |  |  |  |  |  |  | 0.00 | 0.07 | 0.05 |  | 0.00 | 0.07 | 0.05 |
| **Relative change score** |  |  |  |  |  |  |  |  |  |  |  |  |  |  |  |  |  |  |  |
| Intercept | 7.74 | 1.30 | 1.00 |  | 7.74 | 1.30 | 1.00 |  | 2.09 | 1.64 | 0.49 |  | 2.09 | 1.62 | 0.50 |  | 2.09 | 1.63 | 0.50 |
| P-I | 0.32 | 0.08 | 0.97 |  | 0.33 | 0.08 | 0.98 |  | 0.34 | 0.07 | 0.99 |  | 0.00 | 0.09 | 0.03 |  | 0.00 | 0.10 | 0.04 |
| P-II | 0.00 | 0.08 | 0.05 |  | 0.00 | 0.08 | 0.05 |  | 0.00 | 0.08 | 0.05 |  | 0.00 | 0.10 | 0.04 |  | 0.00 | 0.11 | 0.05 |
| Pre-test score |  |  |  |  | -0.39 | 0.12 | 0.98 |  | -0.39 | 0.11 | 0.98 |  | -0.41 | 0.11 | 0.99 |  |  |  |  |
| Group |  |  |  |  |  |  |  |  | 11.29 | 2.60 | 1.00 |  | 11.29 | 2.60 | 1.00 |  | 11.31 | 2.64 | 1.00 |
| P-I x Group |  |  |  |  |  |  |  |  |  |  |  |  | 0.67 | 0.14 | 1.00 |  | 0.63 | 0.15 | 0.98 |
| P-II x Group |  |  |  |  |  |  |  |  |  |  |  |  | 0.00 | 0.15 | 0.05 |  | 0.00 | 0.16 | 0.05 |
| **Residual score** |  |  |  |  |  |  |  |  |  |  |  |  |  |  |  |  |  |  |  |
| Intercept | -0.00 | 0.00 | 0.00 |  | -0.00 | 0.00 | 0.00 |  | -2.96 | 0.61 | 1.00 |  | -2.96 | 0.60 | 1.00 |  | -2.95 | 0.60 | 1.00 |
| P-I | 0.16 | 0.04 | 0.99 |  | 0.16 | 0.04 | 0.99 |  | 0.16 | 0.03 | 1.00 |  | 0.00 | 0.04 | 0.03 |  | 0.00 | 0.04 | 0.03 |
| P-II | 0.00 | 0.04 | 0.05 |  | 0.00 | 0.04 | 0.04 |  | 0.00 | 0.04 | 0.05 |  | 0.00 | 0.05 | 0.05 |  | 0.00 | 0.05 | 0.05 |
| Pre-test score |  |  |  |  | -0.01 | 0.01 | 0.00 |  | -0.01 | 0.02 | 0.00 |  | -0.02 | 0.02 | 0.01 |  |  |  |  |
| Group |  |  |  |  |  |  |  |  | 5.91 | 1.22 | 1.00 |  | 5.91 | 1.22 | 1.00 |  | 5.90 | 1.22 | 1.00 |
| P-I x Group |  |  |  |  |  |  |  |  |  |  |  |  | 0.33 | 0.07 | 1.00 |  | 0.33 | 0.07 | 1.00 |
| P-II x Group |  |  |  |  |  |  |  |  |  |  |  |  | 0.00 | 0.07 | 0.05 |  | 0.00 | 0.07 | 0.05 |

Table 32: Results of all investigated simulations ordered according to their used model and dependent variable, for a **reliability of .90**, and a sample size of ***n* = 500**

| **Coefficient** | **Model 1** | | |  | **Model 2** | | |  | **Model 3** | | |  | **Model 4** | | |  | **Model 5** | | |
| --- | --- | --- | --- | --- | --- | --- | --- | --- | --- | --- | --- | --- | --- | --- | --- | --- | --- | --- | --- |
|  | ***M*** | ***SE*** | ***P*** |  | ***M*** | ***SE*** | ***P*** |  | ***M*** | ***SE*** | ***P*** |  | ***M*** | ***SE*** | ***P*** |  | ***M*** | ***SE*** | ***P*** |
| **Post-test score** |  |  |  |  |  |  |  |  |  |  |  |  |  |  |  |  |  |  |  |
| Intercept | 53.50 | 0.69 | 1.00 |  | 53.50 | 0.69 | 1.00 |  | 50.56 | 0.80 | 1.00 |  | 50.55 | 0.79 | 1.00 |  | 50.55 | 0.89 | 1.00 |
| P-I | 0.21 | 0.06 | 0.96 |  | 0.16 | 0.03 | 1.00 |  | 0.16 | 0.03 | 1.00 |  | -0.00 | 0.04 | 0.03 |  | 0.00 | 0.06 | 0.02 |
| P-II | -0.00 | 0.05 | 0.06 |  | -0.00 | 0.03 | 0.05 |  | -0.00 | 0.03 | 0.05 |  | -0.00 | 0.04 | 0.04 |  | -0.00 | 0.06 | 0.03 |
| Pre-test score |  |  |  |  | 0.90 | 0.04 | 1.00 |  | 0.90 | 0.04 | 1.00 |  | 0.89 | 0.04 | 1.00 |  |  |  |  |
| Group |  |  |  |  |  |  |  |  | 5.88 | 1.10 | 1.00 |  | 5.88 | 1.10 | 1.00 |  | 5.89 | 1.37 | 1.00 |
| P-I x Group |  |  |  |  |  |  |  |  |  |  |  |  | 0.33 | 0.06 | 1.00 |  | 0.41 | 0.10 | 0.98 |
| P-II x Group |  |  |  |  |  |  |  |  |  |  |  |  | 0.00 | 0.06 | 0.04 |  | 0.00 | 0.10 | 0.04 |
| **Absolute change score** |  |  |  |  |  |  |  |  |  |  |  |  |  |  |  |  |  |  |  |
| Intercept | 3.46 | 0.54 | 1.00 |  | 3.46 | 0.54 | 1.00 |  | 0.52 | 0.68 | 0.33 |  | 0.51 | 0.68 | 0.34 |  | 0.51 | 0.68 | 0.34 |
| P-I | 0.16 | 0.03 | 1.00 |  | 0.16 | 0.03 | 1.00 |  | 0.16 | 0.03 | 1.00 |  | -0.00 | 0.04 | 0.03 |  | -0.00 | 0.04 | 0.04 |
| P-II | -0.00 | 0.03 | 0.05 |  | -0.00 | 0.03 | 0.05 |  | -0.00 | 0.03 | 0.05 |  | -0.00 | 0.04 | 0.04 |  | -0.00 | 0.04 | 0.04 |
| Pre-test score |  |  |  |  | -0.10 | 0.04 | 0.78 |  | -0.10 | 0.04 | 0.82 |  | -0.11 | 0.04 | 0.88 |  |  |  |  |
| Group |  |  |  |  |  |  |  |  | 5.88 | 1.10 | 1.00 |  | 5.88 | 1.10 | 1.00 |  | 5.88 | 1.11 | 1.00 |
| P-I x Group |  |  |  |  |  |  |  |  |  |  |  |  | 0.33 | 0.06 | 1.00 |  | 0.32 | 0.06 | 1.00 |
| P-II x Group |  |  |  |  |  |  |  |  |  |  |  |  | 0.00 | 0.06 | 0.04 |  | 0.00 | 0.06 | 0.04 |
| **Relative change score** |  |  |  |  |  |  |  |  |  |  |  |  |  |  |  |  |  |  |  |
| Intercept | 7.67 | 1.17 | 1.00 |  | 7.67 | 1.17 | 1.00 |  | 2.06 | 1.49 | 0.53 |  | 2.05 | 1.48 | 0.54 |  | 2.06 | 1.48 | 0.53 |
| P-I | 0.32 | 0.07 | 0.99 |  | 0.34 | 0.07 | 1.00 |  | 0.33 | 0.07 | 1.00 |  | -0.00 | 0.09 | 0.03 |  | -0.00 | 0.09 | 0.04 |
| P-II | -0.00 | 0.07 | 0.06 |  | -0.00 | 0.07 | 0.05 |  | -0.00 | 0.07 | 0.05 |  | -0.00 | 0.09 | 0.03 |  | -0.00 | 0.09 | 0.04 |
| Pre-test score |  |  |  |  | -0.39 | 0.11 | 0.99 |  | -0.40 | 0.11 | 1.00 |  | -0.41 | 0.10 | 1.00 |  |  |  |  |
| Group |  |  |  |  |  |  |  |  | 11.21 | 2.33 | 1.00 |  | 11.21 | 2.33 | 1.00 |  | 11.21 | 2.35 | 1.00 |
| P-I x Group |  |  |  |  |  |  |  |  |  |  |  |  | 0.67 | 0.12 | 1.00 |  | 0.64 | 0.13 | 1.00 |
| P-II x Group |  |  |  |  |  |  |  |  |  |  |  |  | 0.00 | 0.13 | 0.05 |  | 0.00 | 0.13 | 0.05 |
| **Residual score** |  |  |  |  |  |  |  |  |  |  |  |  |  |  |  |  |  |  |  |
| Intercept | 0.00 | 0.00 | 0.00 |  | 0.00 | 0.00 | 0.00 |  | -2.94 | 0.55 | 1.00 |  | -2.94 | 0.55 | 1.00 |  | -2.94 | 0.55 | 1.00 |
| P-I | 0.16 | 0.03 | 1.00 |  | 0.16 | 0.03 | 1.00 |  | 0.16 | 0.03 | 1.00 |  | -0.00 | 0.04 | 0.03 |  | -0.00 | 0.04 | 0.04 |
| P-II | -0.00 | 0.03 | 0.05 |  | -0.00 | 0.03 | 0.05 |  | -0.00 | 0.03 | 0.05 |  | -0.00 | 0.04 | 0.04 |  | -0.00 | 0.04 | 0.04 |
| Pre-test score |  |  |  |  | -0.01 | 0.01 | 0.00 |  | -0.01 | 0.02 | 0.00 |  | -0.02 | 0.02 | 0.01 |  |  |  |  |
| Group |  |  |  |  |  |  |  |  | 5.88 | 1.10 | 1.00 |  | 5.88 | 1.10 | 1.00 |  | 5.86 | 1.10 | 1.00 |
| P-I x Group |  |  |  |  |  |  |  |  |  |  |  |  | 0.33 | 0.06 | 1.00 |  | 0.33 | 0.06 | 1.00 |
| P-II x Group |  |  |  |  |  |  |  |  |  |  |  |  | 0.00 | 0.06 | 0.04 |  | 0.00 | 0.06 | 0.04 |


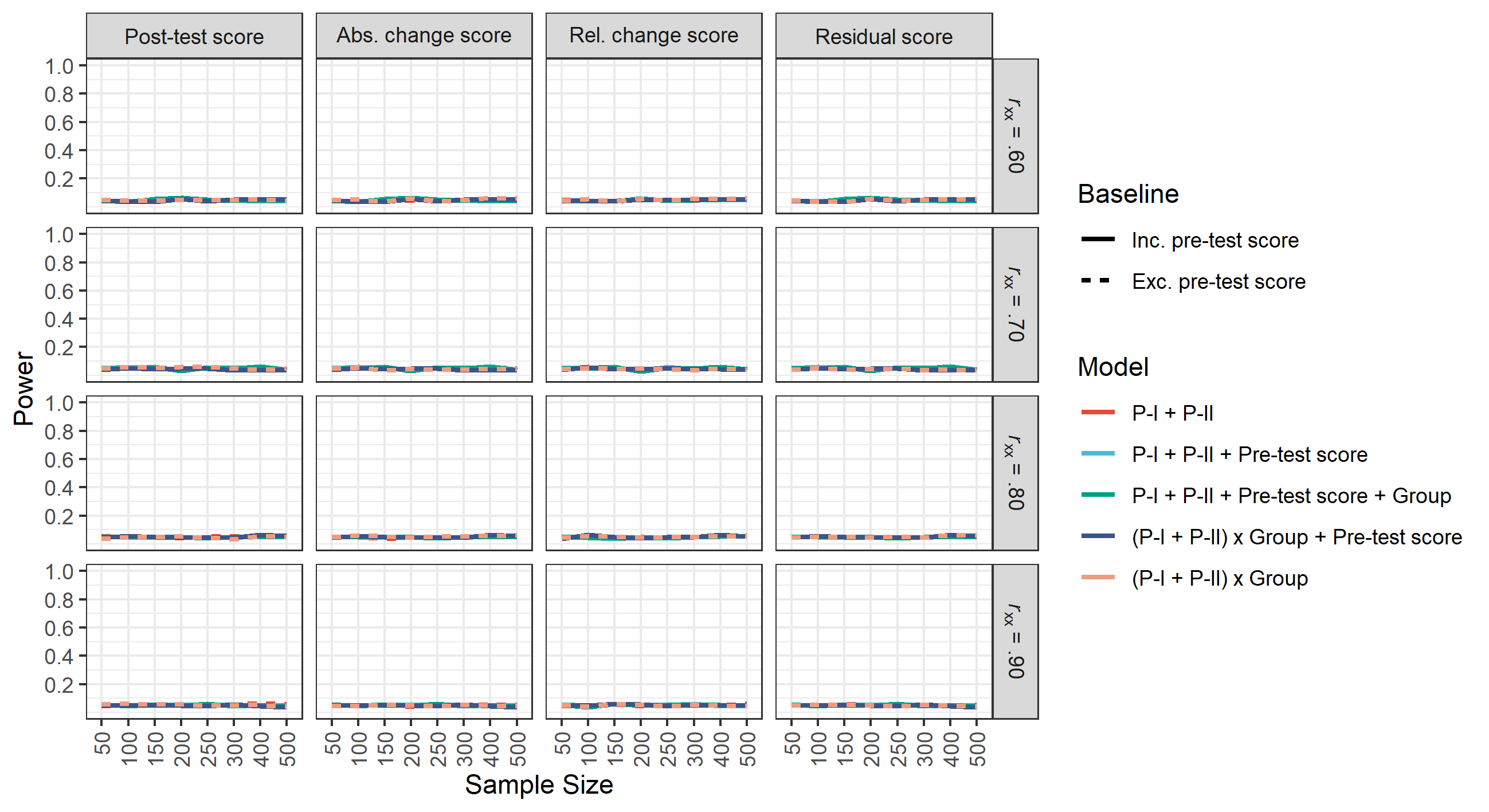


Figure S1: Overview of the power for the P-II or P-II x Group regression coefficient in dependence of the dependent variable, regression model, sample size and reliability.

The probability of obtaining a significant result is constantly at 5 %, indicating that regression model, dependent variable, sample size and reliability do not have an impact on the false discovery rate.


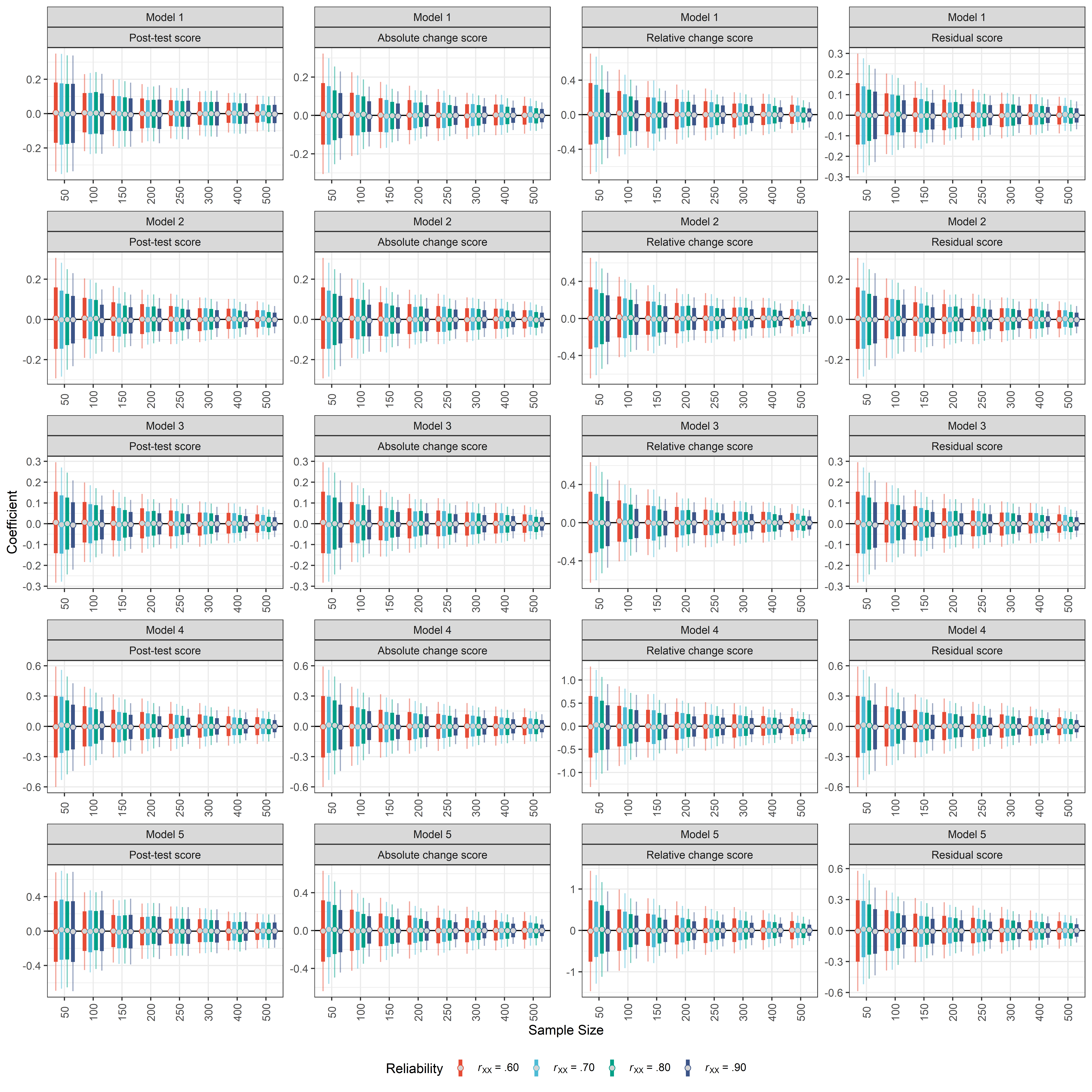


Figure S2: Overview of the regression coefficients of P-II or P-II x Group as a function of the dependent variable, the regression model, the sample size and the reliability.

The different regression models that were tested are displayed in the rows (Model 1 to 5) and the different dependent variables are displayed in the columns. In each subplot, the x-axis indicates the sample size and the y-axis the value of the regression coefficient for the predictor P-II or the P-II x Group interaction, depending on whether the respective model comprised the interaction term or not. For each sample size, the reliability is colour-coded. The dot indicates the mean of the regression coefficient distribution generated by simulating the data. The thick line covers the interval of the mean plus/minus one standard error and the thin line represents the 95% confidence interval.

*Note.* Red colour indicates a reliability of .60; blue colour indicates a reliability of .70; green colour indicates a reliability of .80; purple colour indicates a reliability of .90. Model 1: P-I + P-II; Model 2: P-I + P-II + Pre-test score; Model 3: P-I + P-II + Pre-test score + Group; Model 4: (P-I + P-II) x Group + Pre-test score; Model 5: (P-I + P-II) x Group.


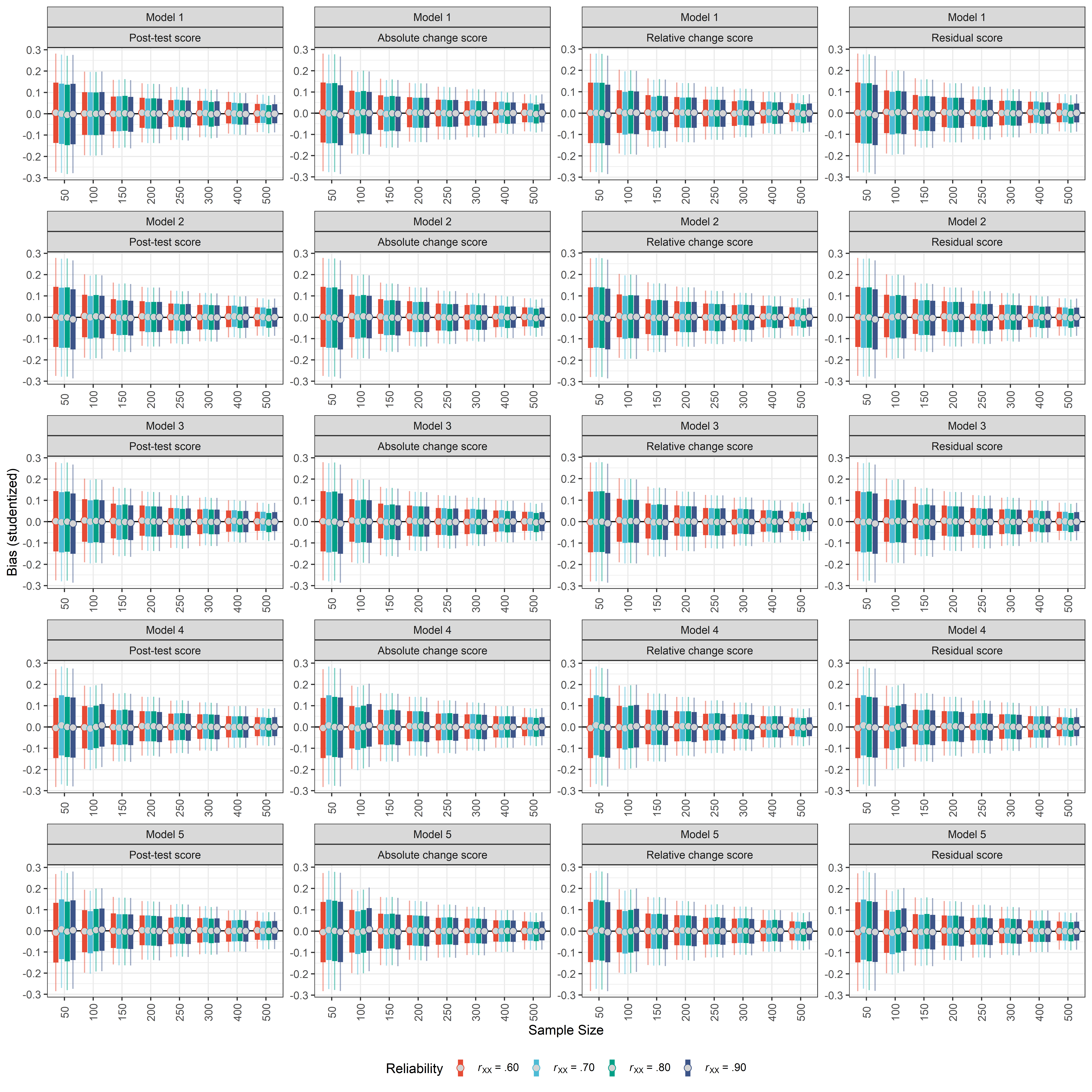


Figure S3: Overview of the studentized bias of the regression coefficients of P-II or P-II x Group as a function of the dependent variable, the regression model, the sample size and the reliability.

The different regression models that were tested are displayed in the rows (Model 1 to 5) and the different dependent variables are displayed in the columns. In each subplot, the x-axis indicates the sample size and the y-axis the studentized bias for the predictor P-II or the P-II x Group interaction, depending on whether the respective model comprised the interaction term or not. For each sample size, the reliability is colour-coded. The dot indicates the mean of the bias distribution. The thick line covers the interval of the mean plus/minus one standard error and the thin line represents the 95% confidence interval. A bias of zero would indicate that the observed regression coefficient is identical to the true regression coefficient.

*Note.* Red colour indicates a reliability of .60; blue colour indicates a reliability of .70; green colour indicates a reliability of .80; purple colour indicates a reliability of .90. Model 1: P-I + P-II; Model 2: P-I + P-II + Pre-test score; Model 3: P-I + P-II + Pre-test score + Group; Model 4: (P-I + P-II) x Group + Pre-test score; Model 5: (P-I + P-II) x Group.
